# Distinct Site Motifs Activate O_2_ and H_2_ on Supported Au Nanoparticles in Liquid Water

**DOI:** 10.1021/acscatal.3c05072

**Published:** 2024-02-15

**Authors:** Jason S. Adams, Haoyu Chen, Tomas Ricciardulli, Sucharita Vijayaraghavan, Abinaya Sampath, David W. Flaherty

**Affiliations:** † Department of Chemical and Biomolecular Engineering, University of Illinois at Urbana-Champaign, Champaign, Illinois 61801, United States; ‡ School of Chemical and Biomolecular Engineering, Georgia Institute of Technology, Atlanta, Georgia 30332, United States

**Keywords:** catalysis, water chemistry, surface science, metals, mechanisms of reactions

## Abstract

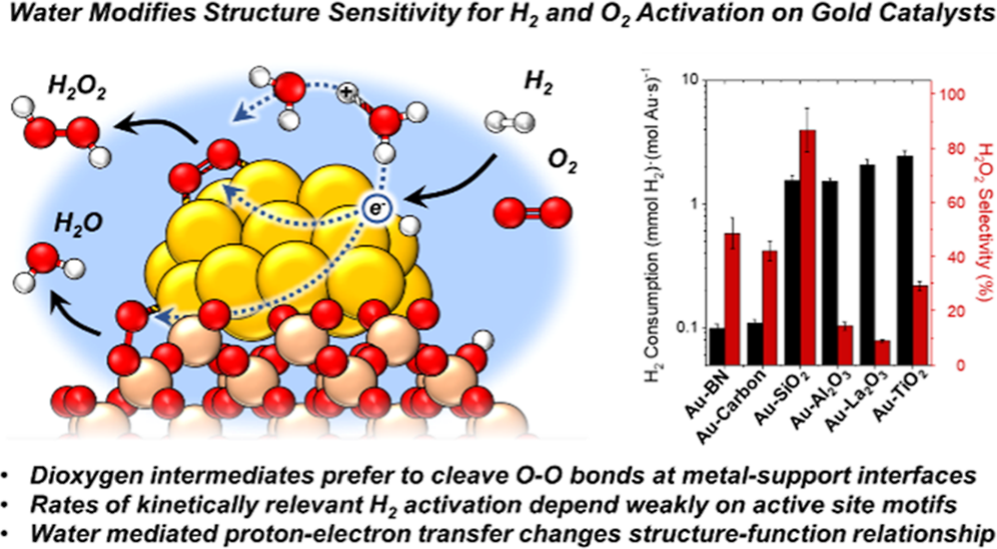

Au nanoparticles catalyze the activation and conversion of small molecules with rates and kinetic barriers that depend on the dimensions of the nanoparticle, composition of the support, and presence of catalytically culpable water molecules that solvate these interfaces. Here, molecular interpretations of steady-state rate measurements, kinetic isotope effects, and structural characterizations reveal how the interface of Au nanoparticles, liquid water, and metal oxide supports mediate the kinetically relevant activation of H_2_ and sequential reduction of O_2_-derived intermediates during the formation of H_2_O_2_ and H_2_O. Rates of H_2_ consumption are 10–100 fold greater on Au nanoparticles supported on metal oxides (e.g., titania) compared to more inert and hydrophobic materials (carbon, boron nitride). Similarly, Au nanoparticles on reducible and Lewis acidic supports (e.g., lanthana) bind dioxygen intermediates more strongly and present lower barriers (<22 kJ mol^–1^) for O–O bond dissociation than inert interfaces formed with silica (>70 kJ mol^–1^). Selectivities for H_2_O_2_ formation increase significantly as the diameters of the Au nanoparticles increase because differences in nanoparticle size change the relative fractions of exposed sites that exist at Au–support interfaces. In contrast, site-normalized rates and barriers for H_2_ activation depend weakly on the size of Au nanoparticles and the associated differences in active site motifs. These findings suggest that H_2_O aids the activation of H_2_ at sites present across all surface Au atoms when nanoparticles are solvated by water. However, molecular O_2_ preferentially binds and dissociates at Au–support interfaces, leading to greater structure sensitivity for barriers of O–O dissociation across different support identities and sizes of Au nanoparticles. These insights differ from prior knowledge from studies of gas-phase reactions of H_2_ and O_2_ upon Au nanoparticle catalysts within dilute vapor pressures of water (10^–4^ to 0.1 kPa H_2_O), in which catalysis occurs at the perimeter of the Au–support interface. In contrast, contacting Au catalysts with liquid water (55.5 M H_2_O) expands catalysis to all surface Au atoms and enables appreciable H_2_O_2_ formation.

## 1 Introduction

Supported Au nanoparticles catalyze reactions of molecules bound to the sites of the metal, support, and their interfaces,^1−4^ enabling many industrially relevant reactions (e.g., oxidation of CO^5−26^ and alcohols,^27,28^ hydrogenation of heteroatoms,^29−33^ hydrogenolysis of allylic carbonates,^34^ Suzuki–Miyaura coupling,^35−37^ and hydrochlorination of alkynes^38^). Haruta demonstrated that supported Au nanoparticles show exceptional rates of CO oxidation,^5,38^ particularly at low temperatures and in the presence of moisture.^6−11,13−17,19,27,28^ In contrast, unsupported Au powders and Au nanoparticles of similar diameters require much greater temperatures to achieve equivalent rates of CO oxidation,^5,39−43^ implying Au–support interfaces lower barriers for kinetically relevant oxidation steps. Indeed, decorating Au(111) with metal oxide materials leads to similar conclusions,^17,44−47^ suggesting that functions at the Au–support interface enable new paths of reacting intermediates sensitive to water molecules.

Generally, the support material can provide acidic or basic functionalities, change the oxidation state of metal atoms, and present oxygen vacancies, among other effects.^1,2,48^ These interfaces also influence the morphology of metal nanoparticles (e.g., size, orientation, and faceting) and transfer charge between the metal, support, and reactive intermediates.^48−53^ These effects change reaction barriers and facilitate paths not observed over unsupported metal nanoparticles or extended surfaces.^7−12,54^ For example, dioxygen binds strongly at the interface of Au nanoparticles and TiO_2_, which form Au–O–O–Ti species at oxygen vacancy sites, assist O–O dissociation paths, and enable facile oxidation of organic substrates.^21−23^ Moreover, spectroscopic evidence shows that different Au–support interfaces (e.g., Au–TiO_2_, Au–Al_2_O_3_) stabilize different fractions of oxygen species (e.g., O*, O_2_*, OH*, and so forth) compared roughened Au foil.^55^ Thus, the Au–support interface enables the catalysis of critical small molecules (e.g., H_2_, O_2_, CO, olefins, and so forth); however, the precise mechanism and site requirements of how these interfaces react with these species remain controversial.^1,2,6^

Mounting evidence suggests Au catalysts interact with water molecules by facilitating proton–electron transfer (PET) reactions that alter barriers of activating O–O, H–H, and O–H bonds relative to anhydrous conditions.^56−58^ For example, trace quantities of water vapor (10^–4^ to 0.1 kPa H_2_O) promote the activation of dioxygen on supported Au nanoparticles, increasing rates for CO oxidation by 1–2 orders of magnitude.^7−12^ Indeed, kinetic isotope effect measurements and density functional theory (DFT) calculations suggest that H_2_O and OH* species cocatalyze the reduction of O_2_* to OOH*, which transforms CO* to CO_2_ at Au–support interfaces.^7−16^ Related PET reactions also occur during reactions of H_2_ and O_2_. For example, infrared spectra of H_2_ dosed over Au–TiO_2_ shows that electrons transfer to the conduction band of Au–TiO_2_ and protons “spillover” to the support as hydroxyl groups.^29,31,54,59−62^ Here, the addition of water simultaneously increases rates of O_2_ activation^6−11,13−17,19,27,28^ but inhibits H_2_ oxidation reactions at the Au–TiO_2_ interface^29,30^ by slowing rates of electron transfer. Regardless, these protons and electrons inevitably react with adsorbed O_2_, forming a litany of species (e.g., O_2_*, OOH*, O*, OH*) that form either H_2_O_2_ or H_2_O.^29,30,59^ Thus, modest vapor pressures (10^–4^ to 0.1 kPa H_2_O) ubiquitously impact rates of small molecules conversion upon supported Au nanoparticles in the gas phase.^7−12^ However, past investigations do not explain the emergence of distinct structure–function relationships for thermochemical reactions of H_2_ and O_2_ on Au catalysts completely enveloped by liquid water (55.5 M H_2_O).

Liquid water greatly alters the electronic structure and resulting catalysis of Au surfaces compared to gas-phase conditions. For example, water lowers the work function of Au by ∼2.84–3.21 eV, consistent with an increase in the electrochemical potential of electrons in the metal relative to the vacuum.^63^ Moreover, the exchange of protons and electrons with reacting species at metal–liquid interfaces leads to the emergence of spontaneous electric fields,^64,65^ which influence the free energy of binding and reacting polar and charged species at solid–liquid interfaces.^66^ Such concepts are better understood in purely electrochemical systems. For example, Au catalysts readily convert O_2_ to H_2_O_2_ or H_2_O under reducing electrochemical potentials within acidic or alkaline electrolyte.^67−69^ However, the postulation of heterolytic paths in thermal catalysis^7,11,27,29,30^ and direct comparisons of such systems to electrochemical reactions on Au-based catalysts have garnered considerable attention recently.^70−72^ For example, Davis and co-workers demonstrated that aqueous-phase OH^–^ and surface-bound OH* cocatalyze alcohol oxidation (e.g., ethanol, glycerol) by forming alkoxide species over Au surfaces while simultaneously transferring protons and electrons to O_2_.^27,28^ We have drawn similar conclusions investigating the mechanisms of H_2_O_2_ formation from H_2_ and O_2_ over Au-based alloys.^73,74^ However, the role of Au–support interactions at solid–liquid interfaces remains underexplored for heterolytic reactions of H_2_ and O_2_ in liquid water.

Here, we describe the role of the Au–support interface during reactions among H_2_ and O_2_ over supported Au nanoparticles within liquid water. Kinetic analysis of H_2_O_2_ and H_2_O formation rates combined with isotopic measurements suggest that supported Au nanoparticles share a common PET mechanism involving kinetically relevant H_2_ activation mediated by H_2_O molecules. Au nanoparticles supported upon metal oxide materials (e.g., La_2_O_3_) show greater rates of H_2_ consumption than more refractory and hydrophobic classes of materials (e.g., BN, carbon), suggesting that proton acceptors (e.g., M–OH, H_2_O) assist in the activation of H–H bonds at the metal–liquid–support interface. Still, site-normalized rates and barriers of H_2_ consumption show a weak dependence on the mean diameter of Au nanoparticles, indicating that H_2_ can activate across all surface Au atoms with the assistance of liquid water molecules that solvate these interfaces. By comparison, the activation of O–O bonds is far more structure sensitive. Interfaces with reducible or Lewis acidic metal oxides favor the formation of H_2_O over H_2_O_2_, which indicates that oxygen vacancies and moieties that enable direct binding of oxygen between Au and the metal atoms of the support control the free energy of dissociating O–O bonds. Consequently, H_2_O_2_ selectivities increase with the size of Au nanoparticles as the fraction of sites at the Au–support interface decreases relative to the total number of metallic surface Au atoms further from these interfaces. Thus, the understanding developed here provides multiple strategies to manipulate the relative rates of H_2_ and O_2_ activation, which may guide the design of new Au catalysts and alloys for other thermal and electrochemical reduction and oxidation reactions.

## 2 Experimental Methods

### 2.1 Catalyst Preparation

#### 2.1.1 Synthesis of TiO_2_- and SiO_2_-Supported Au Catalysts by Strong Electrostatic Adsorption

Catalytic nanoparticles were formed upon anatase TiO_2_ nanoparticles (US Research Nanomaterials Inc., US3838) and mesoporous SiO_2_ (Sigma-Aldrich, Davisil 646) by strong electrostatic adsorption (SEA) using techniques reported elsewhere.^75−77^ Briefly, the TiO_2_ support was placed within a quartz tube furnace and heated at a rate of 5 K min^–1^ to a temperature of 873 K and held for 4 h under a flowing mixture (100 cm^3^ min^–1^) dry air (21 kPa O_2_, 80 kPa N_2_; Airgas, UHP 99.999%) with the intent of removing residual organic ligands present on the material. By comparison, the SiO_2_ support was used as received. Cationic Au ethylenediamine complexes were synthesized by combining HAuCl_4_ (15.6 mM; Sigma-Aldrich, >49 wt % Au) and ethylenediamine (113 mM; Sigma-Aldrich, >99%) in 1 L of deionized (DI) water (>17.8 MΩ cm resistivity) with the intent to exchange chloride ligands with ethylenediamine on the Au center. The resulting solution initially forms a dark brown precipitate that evolves into a clear orange solution once the ligand exchange reaches completion. Separately, DI water (2.5 L) and 1 L of concentrated NH_4_OH (Macron, 28–30% NH_4_OH) were combined in a beaker. Either SiO_2_ or TiO_2_ (85 g) was added to the basic solution with the intent to deprotonate the hydroxylated surface of the support. The suspension was stirred continuously for 10 min, after which the solution of Au–ethylene diamine (1 L) was added while stirring for an additional 10 min. The mixture was then stirred intermittently every 10 min for 1 h and left overnight to provide time for cationic Au species to adsorb to the anionic surface of the support. The solution was then decanted from the solids until a slurry of catalyst remained. Analogous procedures were used to prepare samples with different weight loadings of Au on the support. However, the mass of the Au precursor and support material was adjusted while maintaining the ratio of HAuCl_4_ to ethylenediamine.

Further treatment of these materials differed with the identity of the support. The Au–SiO_2_ solids were filtered and washed with DI water (∼40 cm^3^ g^–1^) to remove residual ions from the material, and these materials were then vacuum-filtered overnight at ambient temperature. The Au–TiO_2_ solids were similarly washed (∼40 cm^3^ g^–1^) and centrifuged to recover a gel, which was dried under vacuum at ambient temperature (∼298 K) for 1 week. The recovered solids (i.e., Au–SiO_2_ or Au–TiO_2_) were then placed within a quartz tube furnace and heated at a rate of 5 K min^–1^ to a maximum temperature (573–1073 K) and held for 4 h under a flowing mixture (300 cm^3^ min^–1^) of He (67 kPa; Airgas, UHP 99.999%) and dry air (7 kPa O_2_, 26 kPa N_2_; Airgas, UHP 99.999%). The range of maximum oxidation temperatures was chosen to produce a series of materials with different mean diameters of Au nanoparticles on each support. Au–TiO_2_ samples prepared at calcination temperatures of 573, 773, 823, 873, 973, and 1073 K are referred to as Au–TiO_2_–O573, Au–TiO_2_–O773, Au–TiO_2_–O823, Au–TiO_2_–O873, Au–TiO_2_–O973, and Au–TiO_2_–O1073, respectively. Similarly, Au–SiO_2_ samples prepared at calcination temperatures of 573, 673, and 1073 K are referred to as Au–SiO_2_–O573, Au–SiO_2_–O673, and Au–SiO_2_–O1073, respectively.

#### 2.1.2 Synthesis of TiO_2_-, Al_2_O_3_-, SiO_2_-, and La_2_O_3_-Supported Au Catalysts by Deposition Precipitation

Deposition precipitation (DP) was used to create supported Au nanoparticles with smaller mean diameters than those formed by SEA of Au-ethylenediamine (vide supra). Briefly, catalytic nanoparticles were formed upon anatase TiO_2_ nanoparticles (US Research Nanomaterials Inc., US3838), γ-Al_2_O_3_ (Catalox, HP 14/150 Alumina), SiO_2_ (Sigma-Aldrich, Davisil 646), and La_2_O_3_ (Sigma-Aldrich, L4000) by deposition precipitation using techniques reported elsewhere.^76,78^ The TiO_2_ support was placed within a quartz tube furnace and heated at a rate of 5 K min^–1^ to a temperature of 873 K and held for 4 h under a flowing mixture (100 cm^3^ min^–1^) dry air (21 kPa O_2_, 80 kPa N_2_; Airgas, UHP 99.999%). The Al_2_O_3_, SiO_2_, and La_2_O_3_ were used as received.

First, the Al_2_O_3_, TiO_2_, and La_2_O_3_ support materials (5 g) were dispersed into 500 mL of DI water and combined with 50 mL of aqueous HAuCl_4_ (15.3 mM) in DI water. In contrast, the SiO_2_ support material (5 g) was added to a 500 mL aqueous solution of NH_4_NO_3_ (62.5 mM) and HAuCl4 (0.35 mM) in DI water. In either case, the resulting suspension was mixed overnight with the intent to allow Au complexes to fully adsorb to the support. The solution was then titrated dropwise with aqueous NH_4_OH (175 mM) until reaching a pH of 10 for the purpose of converting adsorbed HAuCl_4_ into Au(OH)_3_. The solution was then mixed for an additional 1 h and decanted from the solids until a slurry of catalyst remained. The final Au–TiO_2_, Au–Al_2_O_3_, and Au–La_2_O_3_ solids were then washed and centrifuged to extract a paste, which was dried in a convection oven at 323 K overnight in ambient air. The dried solids (Au–TiO_2_, Au–Al_2_O_3_, and Au–La_2_O_3_) were then transferred to quartz boats and heated at a rate of 5 K min^–1^ to 473 K and held for 4 h within a flowing mixture (200 cm^3^ min^–1^) of dilute H_2_ (20 kPa H_2_, 81 kPa He; both Airgas, UHP 99.999%) within a quartz tube placed in a three-zone furnace.

#### 2.1.3 Synthesis of Carbon and BN-Supported Au Catalysts by Incipient Wetness Impregnation

Incipient wetness impregnation (IWI) was used to create supported Au nanoparticles on carbon and boron nitride (BN) with similarly small Au nanoparticles achieved in Section 2.1.2 (vide supra), because these materials do not readily facilitate precipitation deposition. Catalytic nanoparticles were formed upon nonfunctionalized carbon (Cabot Corporation, Vulcan XC-72) and BN (Sigma-Aldrich, 25475) by incipient wetness impregnation techniques, in which the support materials were used as received.^75,79^ Here, a 5 mL solution of HAuCl_4_ (91.4 mM) in DI water was prepared and added dropwise to the support material (3 g) until incipiently wet. The materials were washed with a diluted solution of NH_4_OH (175 mM) and dried in a convection oven at 323 K overnight in ambient air. The dried solids (i.e., Au-BN and Au-carbon) were then heated at a rate of 5 K min^–1^ to 473 K and held for 4 h under a flowing mixture within a flowing mixture (200 cm^3^ min^–1^) of dilute H_2_ (20 kPa H_2_, 81 kPa He; both Airgas, UHP 99.999%) within a quartz tube placed in a three-zone furnace.

#### 2.1.4 Synthesis of Supported Au Catalysts by Deposition of Colloidal Nanoparticles

Colloidal Au nanoparticles were synthesized and then deposited upon anatase TiO_2_ nanoparticles (US Research Nanomaterials Inc., US3838), γ-Al_2_O_3_ (Catalox, HP 14/150 Alumina), and La_2_O_3_ (Sigma-Aldrich, L4000) to enable comparisons of supported Au nanoparticles formed by other methods (vide supra). The TiO_2_ support was placed within a quartz tube furnace and heated at a rate of 5 K min^–1^ to a temperature of 873 K and held for 4 h under a flowing mixture (100 cm^3^ min^–1^) dry air (21 kPa O_2_, 80 kPa N_2_; Airgas, UHP 99.999%). Other supports were used as received. First, a solution of colloidal Au nanoparticles was prepared using the Turkevich citrate method.^80^ Here, a 900 mL solution of trisodium citrate (6.7 mM Na_3_C_6_H_5_O_7_; Sigma-Aldrich, >99.0%) was prepared with DI H_2_O in a flask. The solution was heated in an oil bath at ∼398 K to achieve a rolling boil while stirring with a Teflon-coated magnetic stir bar. A 20 mL solution of HAuCl_4_ (15.2 mM) was prepared in a separate vial of DI H_2_O and added to the boiling solution. After 30 s of mixing, the corresponding support material (2 g) was added to the solution. The slurry was heated and stirred continuously until the liquid completely evaporated to deposit the colloids onto the support material. The resulting precursor materials were then placed in quartz boats and heated at a rate of 5 K min^–1^ to 737 K and held for 4 h within a flowing mixture (100 cm^3^ min^–1^) of dry air (21 kPa O_2_, 80 kPa N_2_; Airgas, UHP 99.999%) in a quartz tube furnace. This treatment was intended to burn off any remaining citrate ligands present on the catalytic material and graft the nanoparticles to the support. Notably, this method deposits a greater quantity of Na and Cl ions onto the supports than the other methods above; however, all materials are washed with over 40 L of DI H_2_O in situ before catalytic measurements (vide infra).

#### 2.1.5 Synthesis of Alloyed PdAu_*x*_ Nanoparticles by Electroless Depositions of Pd(NO_3_)_2_

Pd was deposited onto supported Au nanoparticles by electroless deposition of Pd(NO_3_)_2_ (Sigma-Aldrich, ∼40% Pd) to compare rates and selectivities of PdAu_*x*_ alloys to the parent Au nanoparticle catalysts. First, the Au–TiO_2_ material (2 g) was stirred in DI water (100 cm^3^) for 10 min in a round-bottom flask. Next, the slurry was blanketed with a flowing mixture of dilute H_2_ (1 kPa H_2_, 100 kPa He, 100 cm^3^ min^–1^; Airgas, UHP 99.999%) and constantly stirred. Then, an aqueous solution of ∼50 mL Pd(NO_3_)_2_ (∼0.05 mM Pd(NO_3_)_2_) was added to the vessel in a single injection, and the mixture was stirred for 3 h. The intent of this final step is to allow Pd^2+^ to reduce by reaction with hydrogen atoms upon the Au nanoparticle surfaces to deposit metallic Pd upon Au nanoparticles and form the alloy. The atomic ratios of Au and Pd were selected to synthesize a material with a bulk composition of PdAu_100_ on TiO_2_. Following electroless deposition, the supernatant was decanted, and the remaining slurry was centrifuged before drying it in a convection oven at 323 K overnight. The dried catalyst was then transferred to quartz boats and heated at a rate of 5 K min^–1^ to 573 K and held for 1 h under a flowing stream (300 cm^3^ min^–1^) of dilute air (7 kPa O_2_, 26 kPa N_2_, 67 kPa He; Airgas, UHP 99.999%) within a quartz tube located in a three-zone furnace. This calcination was intended to distribute surface Pd atoms homogeneously on the Au, as discussed elsewhere.^75^

### 2.2 Characterization of Catalytic Materials

#### 2.2.1 Characterization of Au Nanoparticle Size by TEM, XRD, and UV–Vis Spectroscopy

**Figure 1:**
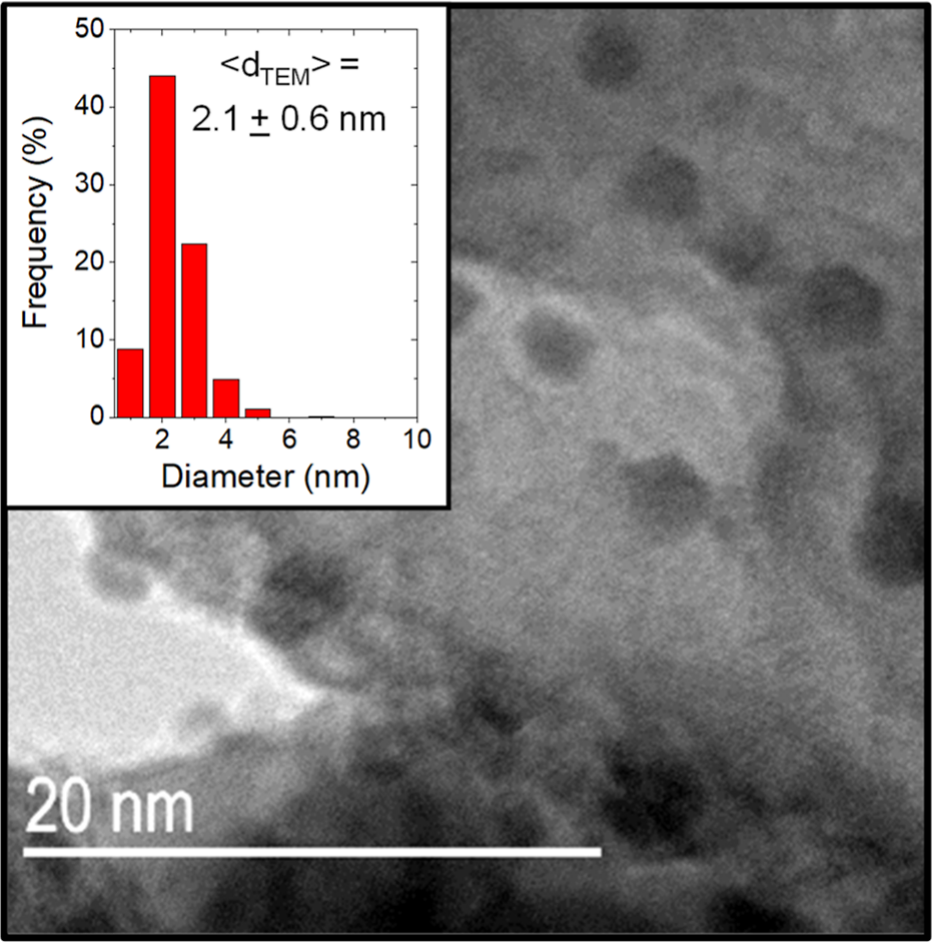
Representative TEM image of ∼3 wt % Au nanoparticles (2–3 nm) supported on calcined anatase TiO_2_ with an inset histogram of the particle size distribution. Figure S1 shows corresponding images and histograms of particle diameters for the other Au-based catalysts prepared by reductive and oxidative treatments between 473 and 1073 K.

The numerical average diameter (⟨*d*_TEM,N_⟩) of supported Au nanoparticles was calculated from the mean diameter of particle size distributions obtained by bright-field transmission electron microscopy (TEM; JEOL, 2010 LaB_6_) determined by measurement of at least 100 nanoparticles but more commonly 200–2000 nanoparticles on each material. Each sample was prepared by grinding the catalyst into a fine powder (<200 mesh), which was dispersed in ethanol (Decon Laboratories, >99.9%) and dripped onto a Cu holey-carbon TEM grid (200 mesh, Ted Pella Inc.). The surface area normalized average diameter (⟨*d*_TEM,S_⟩) for each catalyst was calculated using eq 1.
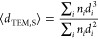
1where *n*_*i*_ is the number of nanoparticles with the diameter *d*_*i*_. Figure 1 shows a representative TEM image of 2 nm Au nanoparticles present on Au–TiO_2_ formed by deposition precipitation and includes an inset histogram of the particle size distribution.

The size of Au nanoparticles was also investigated by diffuse reflectance UV–vis spectroscopy (DRUV–vis) using a diffuse reflection probe (Avantes, solarization-resistant fibers) coupled to a fiber-optic spectrometer (Avantes, AvaFast 2048) with a compact deuterium-halogen light source (Avantes, AvaLight-DHc). Figure S2a,b shows ex situ DRUV–vis spectra of Au–TiO_2_ and Au–SiO_2_ materials, respectively, with nanoparticle diameters ranging between 2 and 25 nm. These extinction spectra show features with a peak absorbance wavelength (λ_max_), related to the surface plasmon resonance frequency of Au nanoparticles, increasing from ∼545 to ∼630 nm for nanoparticle diameters between 2 and 25 nm, respectively (Table 1). Such features are broader on materials treated at the greatest temperatures (>573 K), agreeing with TEM measurements showing increased polydispersity as the particle size increases on these catalysts. In addition, Figure S2c presents photographs that reveal the catalyst color evolves from pink to purple to blue as the values of ⟨*d*_TEM,S_⟩ increase from 2 to 25 nm, consistent with shifts in λ_max_ measured by DRUV–vis.

**Table 1:** Preparation Methods and Characterization of Supported Au Nanoparticles, Including Mean Nanoparticle Diameters from TEM (⟨*d*_TEM,S_⟩ and ⟨*d*_TEM,N_⟩), XRD (⟨*d*_XRD_⟩), and DRUV–Vis Using the Peak Absorbance Wavelength (λ_max_)^a^

preparation of Au material^b^	thermal treatment^c^	⟨*d*_TEM,S_⟩ (nm)^d^	⟨*d*_TEM,N_⟩ (nm)^e^	⟨*d*_XRD_⟩ (nm)^f^^g^	peak absorbance wavelength (nm)
Au–TiO_2_–R473	H_2_ at 473 K 4 h	2.1 ± 0.6	1.9 ± 0.6	6.1	545
Au–TiO_2_–O573	O_2_ at 573 K 4 h	5.0 ± 1.2	4.8 ± 1.2	9.9	565
Au–TiO_2_–O773	O_2_ at 773 K 4 h	7.4 ± 2.0	7.4 ± 2.0	9.9	563
Au–TiO_2_–O823	O_2_ at 823 K 4 h	10.6 ± 2.4	9.9 ± 2.4	10.4	575
Au–TiO_2_–O873	O_2_ at 873 K 4 h	14.5 ± 2.7	13.9 ± 2.7	12.3	592
Au–TiO_2_–O973	O_2_ at 973 K 4 h	18.5 ± 5.2	15.4 ± 5.2	17.6	610
Au–TiO_2_–O1073	O_2_ at 1073 K 4 h	24.7 ± 7.5	19.1 ± 7.5	22.9	633
Au–SiO_2_–R473	H_2_ at 473 K 4 h	2.4 ± 0.7	2.1 ± 0.7	n.d.	520
Au–SiO_2_–O573	O_2_ at 573 K 4 h	5.6 ± 2.2	3.3 ± 2.2	8.7	523
Au–SiO_2_–O673	O_2_ at 673 K 4 h	15.3 ± 3.5	13.3 ± 3.5	13.5	504
Au–SiO_2_–O1073	O_2_ at 1073 K 4 h	26.0 ± 7.0	21.4 ± 7.0	22.8	504

a Reported materials were prepared by strong electrostatic adsorption and deposition precipitation methods, followed by different temperature treatments. Further details of characterization are presented in Table S1.

b Nomenclature for samples of Au nanoparticles supported on TiO_2_ or SiO_2_, prepared by strong electrostatic adsorption and calcined at distinct temperatures.

c Thermal treatments were conducted at 20 kPa H_2_ and 81 kPa He for reductive treatments (R) and at 7 kPa O_2_, 26 kPa N_2_, and 67 kPa He for oxidative treatments.

d Surface-area average normalized mean nanoparticle diameters, calculated by eq 1.

e Nominal mean nanoparticle diameters (⟨*d*_TEM,N_⟩ = ∑_*i*_*d*_*i*_/∑_*i*_*n*_*i*_).

f Average crystallite size of Au nanoparticles using the Scherrer equation of Au(111) facets.

g Very small Au nanoparticles supported on silica were not detected (n.d.).

X-ray diffraction (XRD) patterns of supported Au nanoparticles were obtained using powder samples within a diffractometer (Bruker D8 Advance) with a Cu Kα radiation source (λ = 0.15418 nm) under ambient conditions.

Measurements were conducted between the range of 2θ = 20–80° with a 0.01° resolution and a scan rate of ∼1.5° min^–1^. The resulting diffractograms were compared to reference spectra from the powder diffraction file database and analyzed using the Scherrer equation using shape factors for cuboctahedral nanoparticles.^81^ These diffractograms show an increase in Au(200) crystallinity on materials with increasing calcination temperatures (Figures S3 and S4). Moreover, fits of the Scherrer equation (eq S1.1) on these diffractograms show that the increase in Au crystallite size with oxidation temperature agrees with the increase in nanoparticle diameter shown by TEM and UV–vis.^81^ These XRD measurements also show that the crystal structure of anatase TiO_2_ remains mostly unchanged from 573 to 1073 K (Figure S3a); however, greater temperatures can lead to the formation of the rutile phase (Figure S5).

Table 1 reports the average particle size of Au nanoparticles supported on TiO_2_ and SiO_2_, as characterized by TEM, XRD, and DRUV–vis spectroscopy. Table S1 reports the metal content of these samples, determined by energy dispersive X-ray fluorescence (EDXRF; Shimadzu, EDX-7000) and inductively coupled plasma optical emission spectroscopy (ICP; PerkinElmer, Optima 2000DV) measurements (Section S1). Figure S1 also reports TEM images and histograms for all other samples of supported Au nanoparticles used in this study.

Table 1 also shows that Au nanoparticles increase in size (⟨*d*_TEM,S_⟩ = 2–25 nm) and polydispersity after oxidizing the precursor at greater temperatures (573–1073 K). Samples prepared by deposition precipitation, however, showed smaller and more monodisperse particle size distributions (⟨*d*_TEM,S_⟩ = 2–3 nm; Table S1).

#### 2.2.2 Infrared Spectroscopy of CO Adsorbed to Au Surfaces

Infrared spectra of CO adsorbed to Au–TiO_2_ provide one method to estimate the relative fractions of interfacial Au atoms versus metallic facets of Au that exist far from the interface.^21−23^ Samples were prepared by grinding catalysts (∼60 mg) into a fine powder and pelletizing the powder into a mesh disc (McMaster-Carr, 40 × 40, 25 mm diameter, stainless steel) with a hydraulic press (Carver, model C). These discs were placed within two stainless steel retaining rings and loaded into a custom transmission IR cell with CaF_2_ windows, as described previously.^75,82,83^ The cell was sealed with graphite ferrules (Chromalytic Technology Pty. Ltd.) within a stainless-steel retaining ring on the cell exterior. Here, gaseous CO, He, and O_2_ were introduced to the system by digital mass flow controllers (Alicat, MC Series) mounted on a gas manifold in the flow path of the cell.

Initially, Au samples were heated from ambient at 5 K min^–1^ to 573 K under flowing (40 cm^3^ min^–1^) mixtures of oxygen (20 kPa O_2_, 81 kPa He) and held at 573 K for 1 h with the intent to remove water or adventitious carbon and water from the catalyst surface. The samples were cooled overnight to 303 K in pure He (101 kPa He, 40 cm^3^ min^–1^) before collecting background spectra. Afterward, spectra were collected until the spectra ceased changing. Then, a stream of dilute carbon monoxide (1 kPa CO, 100 kPa He) was introduced from a calibrated gas mixture (Airgas, 1 vol % CO, balance He), and spectra were collected until the sample reached a steady state. Next, the pressure of CO was decreased by adding pure He (Airgas, 99.999%) to attain a range of lower CO partial pressures (0.005–1 kPa CO), and steady-state spectra were obtained at each condition. Finally, samples were returned to the initial composition of CO (1 kPa) to measure differences in the final and initial steady-state spectra at these conditions.

#### 2.2.3 Point of Zero Charge of Catalytic Support Materials

The points of zero charge (PZC) of catalytic support materials were measured using a pH sensor (Oakton, CON 450) submerged in a slurry of solids (∼500 mg) and DI H_2_O. A solution of DI H_2_O was sparged with He (101 kPa He; Airgas 99.999%) to remove dissolved CO_2_ from the atmosphere. This solution was added to a separate beaker containing a given support material in increments of 2.5 cm^3^ while measuring the pH. The slurry transitioned from a paste-like consistency and approached a well-dispersed mixture as the total volume of solution (40 cm^3^) was transferred. The functional dependence of pH with solid mass was extrapolated to the point of incipient wetness using the pore volume of each material (reported by the manufacturer) to find the PZC. Table 2 reports these PZC values on the untreated BN, carbon, SiO_2_, Al_2_O_3_, La_2_O_3_, and TiO_2_, determined by analyzing Figure S6 with techniques reported elsewhere.^84,85^

**Table 2:** Points of Zero Charge of Support Materials from the Dependence of pH as a Function of Solid Content within a Slurry of Support and DI H_2_O

material	point of zero charge (pH)
TiO_2_	3.5
SiO_2_	5.4
La_2_O_3_	8.9
Al_2_O_3_	6.7
carbon	4.2
BN	7.4

### 2.3 Steady-State Reaction Rate Measurements

All steady-state rates of H_2_O_2_ (*r*_H_2_O_2__) and H_2_O (*r*_H_2_O_) formation were measured in a continuous-flow trickle-bed reactor (TBR; 48 cm length, 1 cm inner diameter) housed within a stainless-steel cooling jacket (Figure S7).^75,82^ The reactor was loaded with 0.15–2 g of catalyst (pelletized to 80–120 mesh), which was supported by plugs of glass wool (∼10 mg) and borosilicate glass rods (8 mm diameter). These rods were secured between silver-coated fritted VCR gaskets (Swagelok, SS-4-VCR-2-60M), which were also used to seal the reactor. The temperature was controlled across the reactor by flowing aqueous ethylene glycol (50% volume; Fisher Scientific E178, 99.8%) through the cooling jacket from a recirculating temperature bath (Cole-Parmer Polystat). The temperature within the reactor was monitored using a K-type thermocouple attached to a cooling jacket and in contact with the wall surrounding the catalyst bed. H_2_ and O_2_ compositions in the reactor were controlled by flowing certified gas mixtures (25% H_2_/N_2_, 99.9% D_2,_ 5% O_2_/N_2_, Airgas, 99.999%) through digital mass-flow controllers (Bronkhorst, F-211CV). Warning: pressurized mixtures of H_2_ and O_2_ are explosive when both components simultaneously exceed a mole fraction of 0.05. Before contacting the catalyst, the gaseous reactant stream was contacted and mixed with flowing DI water (>17.8 MΩ cm) that served as the solvent and was delivered by a high-performance liquid chromatography pump (SSI, LS class). The pressure of the resultant gas–liquid mixture was maintained by a back-pressure regulator (Equilibar, LF) and controlled by an electronic pressure regulator (Equilibar, GP1). The upstream pressure of the reactor was monitored by a digital pressure transducer (Omega, PXM409-USBH).

Sampling and analysis of the liquid and gaseous effluent streams were automated and operated continuously. The reactor effluent entered a gas–liquid separator (GLS) machined from polycarbonate and polyvinyl chloride. The gas stream flowed from the GLS to a gas chromatograph (Agilent, 7890B) equipped with a capillary column (Vici, Molecular Sieve 5 Å, 30 m × 0.53 mm × 20 μm) and a thermal conductivity detector that used Ar gas (Airgas, 99.999%) as a reference. Gas chromatograms of catalytic measurements were compared to reference chromatograms using equivalent reaction conditions within a bypass reactor loaded with an equivalent mass of support material, which did not contain catalytic nanoparticles. Comparisons to these control measurements enabled quantification of the conversion of reagents, and together with known molar flow rates, and the calculation of rates of H_2_ (−*r*_H_2__) and O_2_ (−*r*_O_2__) consumption for a given catalyst. An electronically actuated valve (ALSCO Inc., LEV025PL) drained the liquid fraction from the GLS at 10 min intervals. The liquid effluent flowed into an electronic two-position valve (Vici Valco, 10 port EPC10W), which injected ∼1 cm^3^ of the effluent and ∼1 cm^3^ of a colorimetric titrant (12 mM neocuproine [Sigma-Aldrich, >99%], 8.3 mM CuSO_4_ [Fisher Scientific, >98.6%], 25/75 (v/v) ethanol/deionized water mixture [Decon Laboratories, >99.9%]) into test tubes held in an automated fraction collector (Biorad, 2110). Each tube was analyzed by a UV–vis spectrophotometer (Spectronic, 20 Genesys) at a wavelength of 454 nm to measure the H_2_O_2_ concentration using a corresponding calibration curve to determine rates of H_2_O_2_ formation. Measurements were also conducted at high space velocities (35 mL min^–1^ DI H_2_O; 110 SCCM gas) and typically low temperatures (278 K) to suppress secondary reactions that decompose H_2_O_2_, such that measured rates of H_2_O_2_ formation mostly reflect its primary formation pathway. Such conditions were also demonstrated to avoid external mass transfer limitations in prior work.^86^

Product formation rates were determined by normalizing the measured rates by the total molar metal content of the materials. In comparison, turnover rates were estimated by dividing measured rates by estimates for the number of active sites. Independent calculations for turnover rates considered the number of active sites to equal the number of Au atoms at perimeter sites of Au nanoparticles, the Au atoms at corners of faceted Au nanoparticles, or the total number of Au atoms present at surfaces, based upon the particle diameter histograms measured by TEM (vide supra).^87,88^ Reported H_2_O_2_ selectivities were calculated by dividing the formation rate of H_2_O_2_ formation by the rate of H_2_ consumption to determine the fraction of H_2_ gas that forms the desired H_2_O_2_ product (*r*_H_2_O_2__/–*r*_H_2__). For pressure dependence and activation enthalpy measurements, reported rates were corrected by accounting for the rate of deactivation, which assumes these changes arise from an exponential reduction in the number of active sites over time.^89^ These corrections were determined by returning to the initial condition and quantifying the extent of deactivation over time. Generally, most materials showed little deactivation except for Au–SiO_2_ materials, which still showed similar selectivities of H_2_O_2_ formation before and after the measurement (vide infra).

All materials are subjected to multiple activation enthalpy measurements (278–308 K) until they reach a consistent initial and final selectivity to H_2_O_2_ formation to ensure a consistent catalytic state before measurements. These pretreatments also wash all catalytic materials with over 40 L or DI H_2_O, ensuring minimal residual ions are present on materials before measurements. Finally, all samples satisfy the Madon-Boudart criterion, enabling physically meaningful comparisons of catalytic rates and stability in the absence of mass transfer constraints (Section S1).^90−92^

### 2.4 Rate Measurements Using Semibatch Reactors

Transient concentration profiles were measured in round-bottom three-neck flasks (Wilmad Lab Glass, 100 cm^3^) in a semibatch configuration (Figure S8).^82^ The top neck of each flask was connected to a condenser, which was chilled to 273 K with a 20/80 (v/v) mixture of ethylene glycol (>99.8%, Fisher Scientific E178) and DI water that flowed from a refrigerated recirculating bath (Neslab ENDOCAL). The reactant gas mixture (99.999% H_2_ and 5% O_2_/N_2_, Airgas) was supplied to the three-neck flasks using digital mass-flow controllers (Alicat, MC series) connected to custom-built gas dispersion tubes (GDT). Each GDT was placed into 80 cm^3^ of a solvent such that the GDT frits (40–60 μm) were completely submerged. The solvents used in these measurements include DI water, deuterated water (D_2_O; Cambridge Isotopes, 99.9 atom % D), methanol (CH_3_OH; Fisher Chemicals, HPLC grade), acetonitrile (CH_3_CN; Fisher Chemicals, HPLC grade), and acetone (C_3_H_6_O; Fisher Chemicals, HPLC grade). The gas outlet from each flask was vented through a rotameter (Omega, FLDA) that was vented into a fume hood. Comparisons of the inlet and outlet flow rates were ensured to be equal, so no leaks were present. All experiments were conducted at 293 K at ambient pressure, and the solution media was stirred at 1500 rpm with magnetic stir bars to avoid external mass transfer limitations.^82^ Each solvent solution was saturated with gas for 10 min before the catalyst was injected into the flask (100 mg). Aliquots (∼1 cm^3^) were extracted every 5–10 min during the first hour of the measurement and then every hour for 3 h. These samples were titrated with a CuSO_4_-neocuproine solution to determine concentrations of H_2_O_2_ (composition described in Section 2.3). The resulting concentration profiles were then fitted to determine apparent rate constants for the formation of H_2_O_2_ using methods reported elsewhere.^82^

## 3 Results and Discussion

### 3.1 Effect of Au Nanoparticle Size and Support Identity on Rates and Selectivities toward H_2_O_2_ Formation

**Scheme 1:**
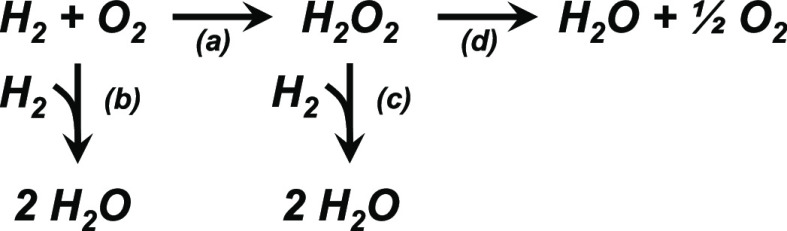
Reactions of H_2_ and O_2_ Over Supported Au Nanoparticles Lead to the Primary Formation of (a) H_2_O_2_ and (b) H_2_O, as Well as the Secondary (c) Hydrogenation of H_2_O_2_ to H_2_O and (d) the Disproportionation of H_2_O_2_ to H_2_O and O_2_

Scheme 1 shows pathways for reactions involving H_2_ and O_2_ that form H_2_O_2_ and H_2_O by primary reactions and additional H_2_O by secondary decomposition reactions that consume H_2_O_2_. Rates of H_2_ and O_2_ consumption are immeasurable on support materials without Au nanoparticles, indicating the supports are catalytically inert by themselves. By comparison, Figure S9 shows that a physical mixture of the support material and colloidal Au nanoparticles present reaction rates 100 times lower than similar-sized Au nanoparticles synthesized by DP, SEA, or IWI methods (Section S2). Calcination of colloidal materials at 773 K should oxidatively remove any remaining citrate ligands before catalysis. Consequently, the low reactivity of colloidal materials may result from poor adhesion of Au nanoparticles to the support rather than site-blocking from residual capping agents. Thus, activation of H_2_ and O_2_ likely requires Au nanoparticles covalently bound to the support material to form a catalytically active Au-support interface.

**Figure 2:**
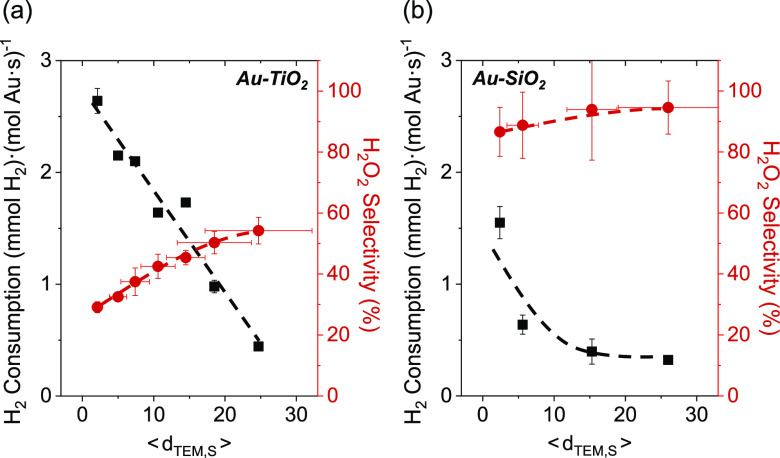
Steady-state rates of H_2_ consumption and H_2_O_2_ selectivities as a function of the surface-area normalized Au nanoparticle diameter on (a) Au–TiO_2_ and (b) Au–SiO_2_ materials (200 kPa H_2_, 60 kPa O_2_, 278 K). Dashed lines represent trends and are intended to guide the eyes.

Figure 2 shows rates of H_2_ consumption and selectivities of H_2_O_2_ formation depend strongly on the mean diameter of Au nanoparticles supported on TiO_2_ and SiO_2_ (200 kPa H_2_, 60 kPa O_2_, 278 K; catalysts synthesized by SEA and DP methods). Rates of H_2_ consumption (normalized by total moles of Au content) decrease as the size of Au nanoparticles increases on both materials, which demonstrates that the number of active sites for H_2_ activation decreased or the average rate of H_2_ activation per active site is lower for larger Au nanoparticles.^13,93^ Rates of H_2_ consumption on TiO_2_-supported Au nanoparticles are greater than those of SiO_2_-supported Au nanoparticles with similar diameters, and these disparities increase as the average diameter of the nanoparticles decreases (Figure 2). Comparisons of rates normalized by estimates for the numbers of surface, perimeter, or corner Au atoms present (vide infra) suggest that all surface Au atoms contribute to H_2_ activation and consumption at these conditions (i.e., samples fully immersed in liquid water). Therefore, all surface Au atoms may catalyze reactions of H_2_ by water-assisted mechanisms, but chemical functions at the Au–support interface also play a crucial role in activating H–H bonds.

Comparisons between Au nanoparticles of similar mean diameters show that Au–TiO_2_ gives lower H_2_O_2_ selectivities (Figure 2a, ∼30–55%) than Au–SiO_2_ (Figure 2b, ∼85–95%), indicating the Au–TiO_2_ interface facilitates the rupture of O–O bonds within surface dioxygen species more readily than sites present on Au–SiO_2_. Moreover, H_2_O_2_ selectivities increase with the mean diameter of Au nanoparticles for both Au–SiO_2_ and Au–TiO_2_ catalysts. These trends suggest that catalytic sites at or near the Au–support interface offer greater rates of O–O bond rupture per exposed Au atom than Au atoms far from this interface (e.g., Au terrace sites). Such conclusions concur with past reports that distinct Au–support interfaces strongly affect the adsorption and activation of small molecules (e.g., O_2_, CO, H_2_, etc.),^7,11,12,29,30^ mirroring comparisons between rates of CO oxidation on Au–TiO_2_ and Au–SiO_2_.^10^ Thus, the Au–support interface is critical to O–O bond activation steps.

**Figure 3:**
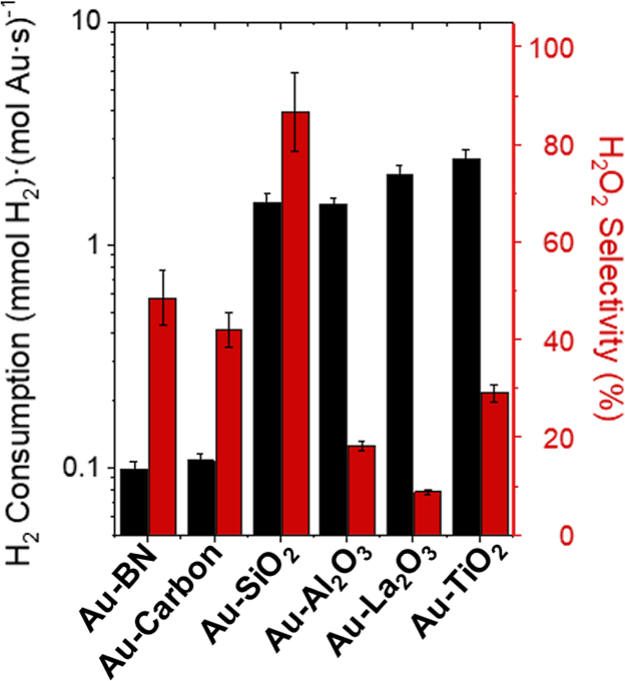
Steady-state rates of H_2_ consumption (black) and H_2_O_2_ selectivities (red) on 2–3 nm Au nanoparticles supported on BN, carbon, SiO_2_, Al_2_O_3_, La_2_O_3_, and TiO_2_ (200 kPa H_2_, 60 kPa O_2_, 278 K).

Figure 3 shows rates of H_2_ consumption are greater by one to 2 orders of magnitude when 2–3 nm Au nanoparticles are supported on metal oxides (e.g., SiO_2_, Al_2_O_3_, La_2_O_3_, TiO_2_) in comparison to Au upon refractory (e.g., BN, carbon) supports (200 kPa H_2_, 60 kPa O_2_, 278 K; catalysts synthesized by DP and IWI methods). These observations suggest oxygen functionalities (or other nucleophilic or Brønsted basic moieties)^98−101^ near Au nanoparticles may assist in activating H–H bonds,^29,30,94−97^ while the more refractory and hydrophobic interfaces of carbon and BN weakly activate H_2_.

Although PET pathways likely mediate the elementary steps described here, the interfacial pH of the support (related to Brønsted acidity) weakly influences the kinetics of activating O–O bonds. Rates of H_2_ consumption do not correlate with differences in the point of zero charge (Figure S10a), which indicates the concentration of protons at the surface weakly affects H–H bond activation. Support materials with greater points of zero charge (La_2_O_3_ > BN > Al_2_O_3_ > SiO_2_ > carbon > TiO_2_; Table 2) show somewhat lower selectivities of H_2_O_2_ formation (Figure S10b), consistent with greater rates of H_2_O_2_ decomposition on Au and PdAu_*x*_ nanoparticles supported on materials with greater isoelectric points.^79^ Thus, Brønsted acid sites may weakly suppress O–O bond dissociation. However, differences in rates and selectivities of H_2_O_2_ formation may depend more on differences in the reducibility, Lewis acidity, or other chemical properties that affect the free energy of binding and activating H_2_ and O_2_ at Au–support interfaces.

Together, these observations show the reactions of H_2_ and O_2_ depend on multiple factors. First, Au atoms must be present and covalently bound to the support. Second, all surface Au atoms can activate H–H bonds but require assistance from a nearby proton acceptor (e.g., H_2_O, OH group), which may differ in quantity and proton affinity depending on the identity of the support. Third, Au atoms at the Au–support interface preferentially activate O–O bonds but are sensitive to the chemical functionality of the support (e.g., reducibility, Brønsted basicity, Lewis acidity, and so forth).

### 3.2 Rate Measurements and Mechanistic Interpretation

**Figure 4:**
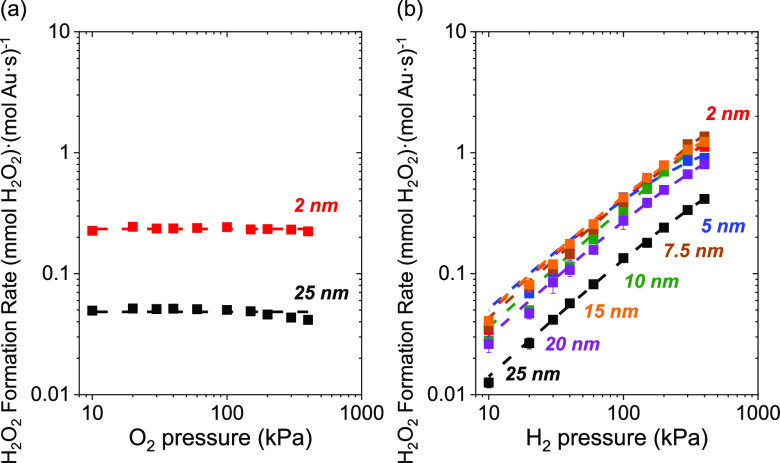
Steady-state rates of H_2_O_2_ formation on 2 nm (red), 5 nm (blue), 7.5 nm (brown), 10 nm (green), 15 nm (orange), 20 nm (purple), and 25 nm (black) Au nanoparticles supported on TiO_2_ as a function of the pressure of (a) O_2_ (10–400 kPa H_2_, 60 kPa O_2_) and (b) H_2_ (10–400 kPa O_2_, 60 kPa H_2_) at 278 K. Dashed lines fitted to eq 7.

**Figure 5:**
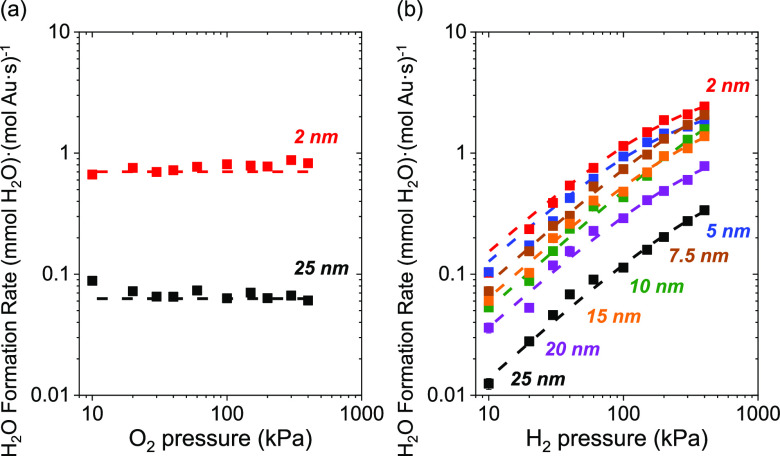
Steady-state rates of H_2_O formation on 2 nm (red), 5 nm (blue), 7.5 nm (brown), 10 nm (green), 15 nm (orange), 20 nm (purple), and 25 nm (black) Au nanoparticles supported on TiO_2_ as functions (a) pressure of O_2_ (60 kPa H_2_), and (b) pressure of H_2_ (60 kPa O_2_) at 278 K. Dashed lines fitted to eq 8.

Next, we investigated mechanisms for reactions among H_2_ and O_2_ on Au nanoparticles on diverse support materials. Steady-state rates of H_2_O_2_ (Figure 4) and H_2_O (Figure 5) formation depend on the pressures of H_2_ and O_2_ in ways consistent with reactions involving kinetically relevant H_2_ activation and subsequent O_2_ reduction steps over Au nanoparticles supported on TiO_2_. Figures 4a and 5a show that rates of H_2_O_2_ and H_2_O formation do not depend on the pressure of O_2_ (10–400 kPa O_2_, 60 kPa H_2_, 278 K), which indicates that oxygen-derived intermediates saturate active sites on Au–TiO_2_. Figures 4b and 5b show H_2_O_2_ and H_2_O formation rates increase linearly with the pressure of H_2_ (10–400 kPa H_2_, 60 kPa O_2_, 278 K), signifying that H_2_ or H_2_-derived species participate in a kinetically relevant and mostly irreversible process. These trends hold for Au nanoparticles of all diameters (⟨*d*_TEM,S_⟩ = 2–25 nm) on TiO_2_ and for 2–3 nm Au nanoparticles on all other supports examined (SiO_2_, Al_2_O_3_, La_2_O_3_, BN, and carbon; Figure S11), suggesting a consistent mechanism across all supported Au catalysts.

Figure S12 shows that H_2_O_2_ selectivities do not depend on the pressure of O_2_ (10–400 kPa O_2_, 60 kPa H_2_, 278 K) but increase slightly as H_2_ pressures increase (10–400 kPa H_2_, 60 kPa O_2_, 278 K) on supported Au nanoparticles. Thus, increasing coverages of H_2_-derived species may stabilize intermediates with O–O bonds (e.g., O_2_*, OOH*, and H_2_O_2_*), as reported on Pd and other noble metals.^75,82,98^ Product formation rates increase linearly with the pressure of H_2_ over Au nanoparticles (Figures 4b and 5b) across a broader range of pressures (10–400 kPa H_2_, 60 kPa O_2_) than observed for Pd- and Pt-based nanoparticles (10–150 kPa H_2_, 60 kPa O_2_).^33,75,82,98−100^ Thus, the coverage of H_2_-derived species remains low on Au nanoparticle catalysts compared to Pd- and Pt-based materials.^33,34,93,100^

**Figure 6:**
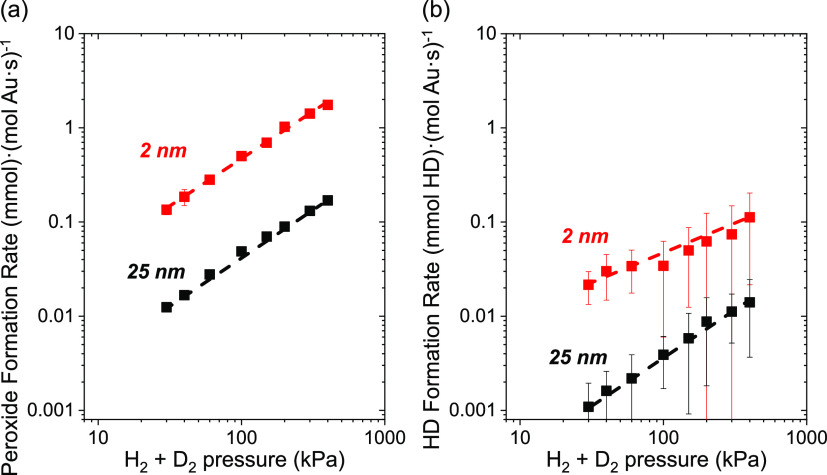
Steady-state formation rates of (a) hydrogen peroxides (i.e., sum of H_2_O_2_, HDO_2_, and D_2_O_2_), and (b) HD on 2 nm (red) and 25 nm (black) Au nanoparticles supported on TiO_2_ as functions of the combined pressure of H_2_ and D_2_ (10–200 kPa H_2_, 10–200 kPa D_2_, 60 kPa O_2_; equimolar H_2_ to D_2_) at 278 K. Dashed lines intended to guide the eyes.

Figure 6 shows that formation rates of peroxides (i.e., H_2_O_2_, HDO_2_, and D_2_O_2;_Figure 6a) and HD (Figure 6b) increase in proportion with the combined pressure of H_2_ and D_2_ (10–200 kPa H_2_, 10–200 kPa D_2_, 60 kPa O_2_, 278 K; equimolar H_2_ and D_2_). Moreover, the total formation rates of peroxides exceed HD formation rates by more than 10-fold. Thus, the formation of O_2_ reduction products likely involves the kinetically relevant activation of H_2_ and D_2_ upon sites surrounded by high coverages of O_2_-derived intermediates. This interpretation also agrees with rates of oxygen reduction that exhibit a normal primary kinetic isotope effect (*k*_H_2__/*k*_D_2__ ∼ 1.5 for both peroxide and water formation, Table 3) when D_2_ replaces H_2_ as the reductant (200 kPa H_2_ or D_2_, 60 kPa O_2_, 298 K, Figure S13). These observations demonstrate that H_2_ and D_2_ activation occurs in a mostly irreversible manner. The H/D atoms produced react immediately to form intermediates that reduce O_2_-derived surface species on Au nanoparticles at rates 20–30 times faster than rates for their recombination to H_2_/HD. Furthermore, the similar kinetic isotope effects for primary formation rates of peroxides and water suggest these pathways share common elementary steps or possess similar transition states for their kinetically relevant steps. Consequently, we conclude that hydrogen activation primarily limits the reduction of O_2_.

**Table 3:** Effects of Isotopic Substitution on Rate Constants of H_2_O_2_ and H_2_O Formation on 2 and 25 nm Au Nanoparticles Supported on TiO_2_ (200 kPa H_2_ or 200 kPa D_2_, 60 kPa O_2_, 278 K)

reaction	Au nanoparticles	
	2 nm	25 nm
H_2_ + O_2_ → H_2_O_2_	1.5 ± 0.1	1.6 ± 0.1
H_2_ + 1/2O_2_ → H_2_O	1.5 ± 0.3	1.5 ± 0.3

**Figure 7:**
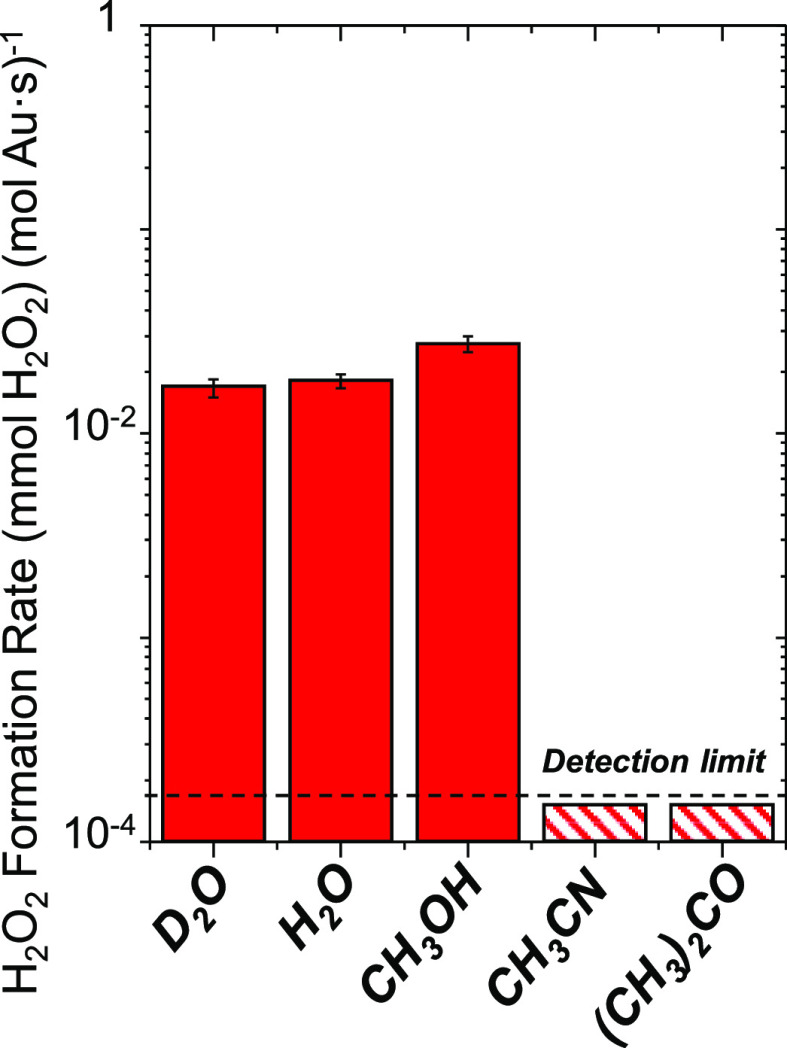
H_2_O_2_ formation rates of reactions of H_2_ and O_2_ in a semibatch reactor using methanol, water, acetonitrile, and acetone as solvents (4.8 kPa H_2_, 4.8 kPa O_2_, 80 cm^3^ solvent, 100 mg of 2 nm Au–TiO_2_, 298 K). The dashed line shows the detection limit of the reactor. Figure S14 shows the transient concentration profiles fitted for these data.

Figure 7 shows rates of H_2_O_2_ formation over 2 nm Au–TiO_2_ in protic solvents (water, heavy water, methanol) and aprotic solvents (acetonitrile, acetone) within a semibatch reactor (4.8 kPa H_2_, 4.8 kPa O_2_, 298 K). H_2_O_2_ forms at rates at least 100-fold greater in protic solvents than aprotic solvents. These findings suggest H_2_O_2_ forms on Au nanoparticles by proton transfer reactions, as shown previously for Pd, Pt, and bimetallic nanoparticles.^75,82,86,90,101−103^ The rates of peroxide formation are marginally greater in H_2_O versus D_2_O solvents (*r*_H_2_O_2__^H_2_O^/*r*_H_2_O_2__^D_2_O^ = 1.1 ± 0.3), indicating that proton transfer steps weakly affect the kinetically relevant step of peroxide formation. These observations agree with analogous isotopic studies of electrochemical oxygen reduction on polycrystalline Au and other noble metals, which suggest electron transfer steps limit oxygen reduction rates.^75,104,105^ In comparison, the formation of water from the secondary hydrogenation of H_2_O_2_ (H_2_ + H_2_O_2_ → 2H_2_O) exhibits significant differences between rates in water and heavy water (*r*_H_2_O_2__^H_2_O^/*r*_H_2_O_2__^D_2_O^ = 3.4 ± 0.4; Figure S14), indicating proton transfer to peroxide presents a greater degree of rate control than proton transfer to dioxygen.

These kinetic observations (Figures 4–7 and Table 3) suggest that H_2_O_2_ and H_2_O formation involve kinetically relevant activation of H_2_ and coupled proton transfer reactions to O_2_-derived surface intermediates that saturate active sites.

**Scheme 2:**
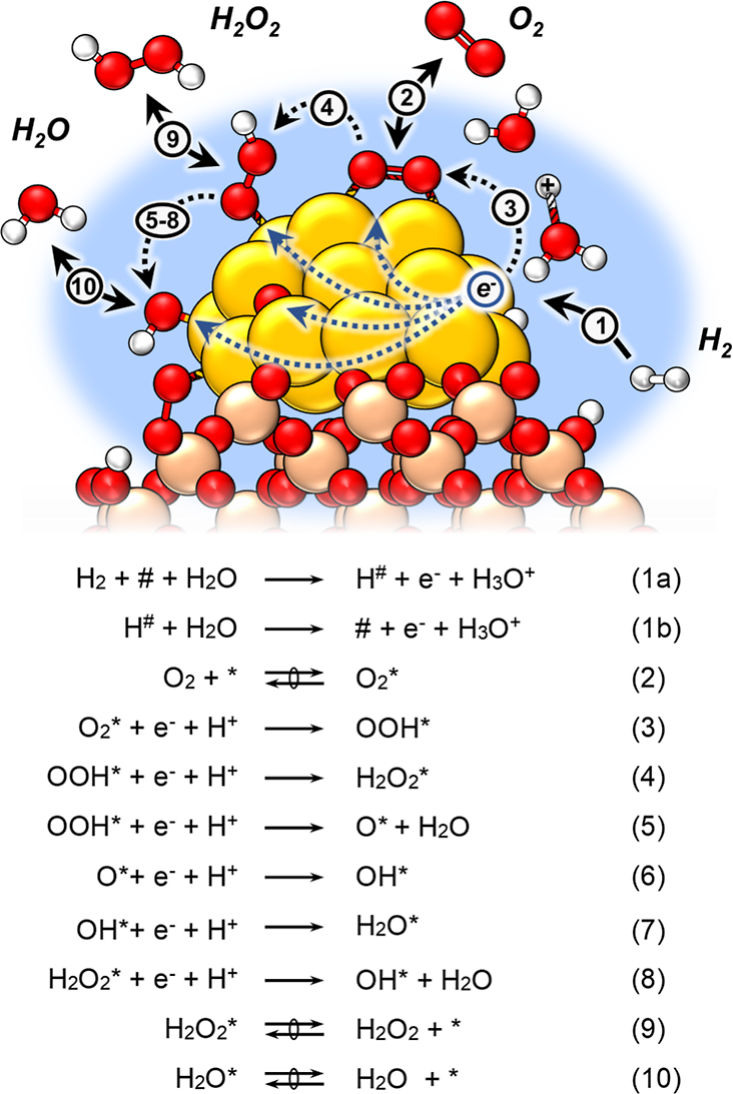
Proposed Elementary Steps for H_2_O_2_ and H_2_O Formation during Reactions of H_2_ and O_2_ on Supported Au Nanoparticles, Which Couple Heterolytic (i.e., Electrochemical) Hydrogen Oxidation and Oxygen Reduction Reactions Sites that bind hydrogen and oxygen-derived species are denoted as # and *, respectively. The symbols 
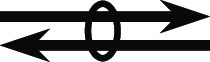
 and → indicate that elementary steps are quasi-equilibrated or mostly irreversible, respectively. Note that this scheme depicts proton-electron transfer steps mediated by water molecules on Au surface; however, hydroxyl groups on the support may facilitate equivalent steps with oxygen species at the Au–support interface.

Scheme 2 shows a sequence of elementary steps consistent with the observed dependence of H_2_O_2_ and H_2_O formation rates on the pressures of H_2_ and O_2_, isotopic measurements, and the need for a protic solvent to mediate O_2_ reduction. This scheme invokes sites that bind hydrogen as # and sites that bind oxygen species as *.^67−69,75,105−107^ This two-site model accounts for the noncompetitive adsorption of H_2_- and O_2_-derived species and invokes heterolytic H_2_ oxidation steps,^29,30^ yielding analytical rate expressions for H_2_O_2_ and H_2_O formation.^29,31,60−62,108^

The kinetically relevant dissociation of H_2_ (step 1a) proceeds with the assistance of water molecules to generate chemisorbed H^#^ atoms, which then oxidize (step 1b) to form hydronium ions and electrons. These steps occur mostly irreversibly (Figure 6) and resemble the electrochemical Heyrovsky and Volmer reactions.^75,109^ The resulting protons (H^+^) may react with water in the solution to form H_3_O^+^ or with hydroxyl groups (M–OH) on the support to form M–OH_2_^+^.^110^ Simultaneously, quasi-equilibrated adsorption of O_2_ (step 2) forms O_2_*,^106^ which reduces to form OOH* through PET steps (step 3).^29,30,75,82,104^ Prior DFT calculations suggest that these O_2_ reduction steps occur irreversibly since they present low barriers and occur exothermically.^29,30^ Still, the hydrogen bonding of liquid water stabilizes O–O bonds and lowers barriers of proton transfer steps to O_2_-derived species bound to metal surfaces relative to the gas phase.^82^ Subsequently, the resulting OOH* reduces to H_2_O_2_* (step 4) or dissociates into H_2_O and O* (step 5) that reduce further and form OH* (steps 6 and 8).^75,111^ The dissociation of dioxygen intermediates (steps 5 and 8) is highly exothermic, and prior isotope labeling studies using mixtures of ^18^O_2_ and ^16^O_2_ suggest that O–O bonds do not reform once cleaved.^75,112^ This scheme assumes that dioxygen species directly form water upon dissociation (e.g., OOH* + e^–^ + H^+^ → O* + H_2_O). Ultimately, the O* and OH* species reduce to water (H_2_O*) by step 7. Bound H_2_O_2_* and H_2_O* then undergo quasi-equilibrated desorption into the solution (steps 9 and 10) to yield H_2_O_2_ and H_2_O. The crucial implications of this mechanism are described below, and the derivation appears with details from Section S3.

Scheme 2 and the dependence of rates on reactant pressures (Figures 4–6) suggest that kinetically relevant hydrogen consumption (−*r*_H_2__) limits the total rate of oxygen reduction (−*r*_O_2__) on all supported Au nanoparticle catalysts examined. Thus, the reaction rate depends mainly on water-assisted activation of H_2_ (step 1a) during the formation of H_2_O_2_ and H_2_O, as shown by
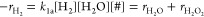
2where *k*_*x*_ is the rate constant of step *x*, and the total rate is a function of the number of unoccupied sites that bind H_2_ ([#]) and the activities of H_2_ and H_2_O ([H_2_] and [H_2_O], respectively). Here, the intrinsic rate constants *k*_*x*_ reflect the weighted reactivity of all active sites. Note that M–OH functions at the Au–support interface can also activate H–H bonds instead of H_2_O, as suggested elsewhere.^29,30^Equation 2 then relates to the rate of electron transfer to O_2_* species, given by
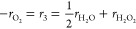
3where −*r*_O_2__ depends on step 3 and determines the H_2_O_2_ and H_2_O formation rates (*r*_H_2_O_2__ and *r*_H_2_O_). Rearrangement of eq 3 yields the product formation rates
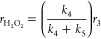
4and
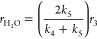
5where eqs 4 and 5 suggest that the average number of electrons transferred during oxygen reduction reflects the ratio of the rates of OOH* hydrogenation (*k*_4_) to dissociation (*k*_5_), which determines the primary selectivity of H_2_O_2_.

Expansion of *r*_3_ in terms of its relevant surface intermediates gives
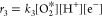
6where the rate of oxygen reduction depends on the activity of electrons ([e^–^]) generated from hydrogen activation and the number of adsorbed dioxygen intermediates ([O_2_*]). These species then react with protons ([H^+^]) transferred either from hydronium ions (H_3_O^+^) or protonated hydroxyl species on the support (M–OH_2_^+^). eq 6, therefore, suggests that Au nanoparticles should show changes in the electrochemical potential of its electrons (related to [e^–^]) that depend on the rates of kinetically relevant oxygen reduction and hydrogen activation steps, explored in detail elsewhere.^70,74,75^

Application of the pseudosteady-state hypothesis yields coverages for each reactive species, leading to expressions
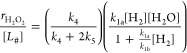
7and
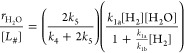
8where the product formation rates depend on the total number of sites that bind H_2_ ([*L*_#_]) and multiple rate constants and reactant activities. Specifically, eqs 7 and 8 indicate that H_2_O_2_ and H_2_O turnover rates depend on the rate of hydrogen consumption (shown by eq 2) multiplied by their selectivities. Here, the total H_2_O_2_ selectivity reflects the average H_2_O_2_ selectivity (*k*_4_/(*k*_4_ + 2*k*_5_)) of all catalytic active sites that perform oxygen reduction.

Equations 7 and 8 predict that H_2_O_2_ and H_2_O turnover rates should increase in proportion with [H_2_] and approach constant values as the H^#^-species saturate binding sites at the greatest pressures. This conclusion agrees with Figures 4 and 5 where rates increase linearly with H_2_ pressures, which suggests H^#^ species exist at low fractional coverages across this range of conditions (10–400 kPa H_2_, 60 kPa O_2_, 278 K). The low coverages of H^#^ on Au surfaces agree with the kinetic relevance of H_2_ dissociation, rapid consumption of H^#^ by heterolytic oxidation, and weak binding of H atoms to supported Au catalysts.^33,99,100^ Such expressions suggest rates do not depend on the pressure of O_2_, consistent with Figures 4 and 5 (10–400 kPa O_2_, 60 kPa H_2_, 278 K).

Therefore, eqs 7 and 8 take the form
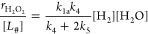
9and
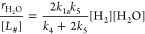
10where turnover rates depend directly on the pressure of H_2_ at the conditions used in this study. Indeed, eqs 9 and 10 also explain the similar H_2_/D_2_ kinetic isotope effects (Table 3) for H_2_O_2_ and H_2_O formation since the isotopic label should only affect the value of *k*_1a_.

This mechanism resembles that proposed by Chandler et al.^29,30^ We observe a similar importance of chemical functions at the Au–support interface during O–O bond activation, proportional dependencies on H_2_ pressure (3–20 kPa H_2_, 10 kPa O_2_, 333 K), and a strong influence of H_2_O on catalysis.^29,31^ However, the condensed water leads to the significant formation of H_2_O_2_, suggesting high concentrations of H_2_O molecules stabilize the formation of dioxygen species on metallic Au far from the Au–support interface and also stabilize O–O bonds better than under dry or humid conditions. By comparison, work by Chandler et al. suggests that H_2_O_2_ dissociation is inevitable and is the primary pathway of H_2_O formation under dry conditions. Furthermore, our findings suggest that water molecules enable the activation of H_2_ across all surface Au atoms (vide infra). In contrast, prior studies strictly attribute the perimeter of the Au–support interface as the active site for all catalysis of H_2_ and O_2_.

### 3.3 Rate Ratios of H_2_O_2_ and H_2_O Formation Correlate with the Fraction of Perimeter Sites at the Au–Support Interface

**Figure 8:**
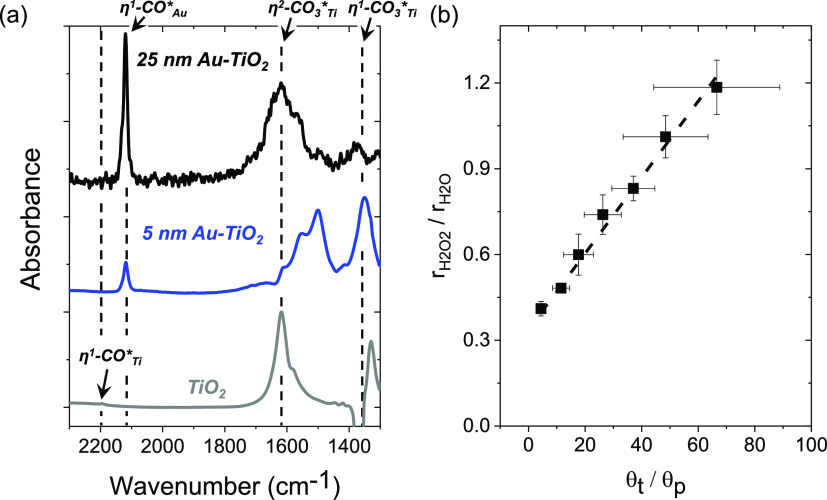
(a) Ex situ infrared spectra of CO adsorbed upon surfaces of TiO_2_ and TiO_2_-supported Au nanoparticles (3 wt % Au, 0.01 kPa CO, 101 kPa He, 303 K) following an oxidative treatment (20 kPa O_2_, 573 K). (b) Ratios of H_2_O_2_ and H_2_O formation as a function of the ration of surface and perimeter (θ_t_/θ_p_) sites at varying Au nanoparticle diameters (200 kPa H_2_, 60 kPa O_2_, 278 K).

Infrared spectra of adsorbed CO were used to probe the quantity and form of site motifs on TiO_2_-supported Au nanoparticles and how these sites change with the size of Au nanoparticles. Figure 8A shows steady-state infrared spectra of CO on TiO_2_ and 5 and 25 nm Au nanoparticles supported on TiO_2_ (0.01 kPa CO, 303 K) obtained following in situ oxidative pretreatments (Section 2.2.2) and saturation at higher pressures of CO (100 kPa). These spectra show vibrational features consistent with CO adsorbed linearly to Au atoms within Au nanoparticles (η^1^-CO_Au_*; ν(C–O) = 2119 cm^–1^) and a weak peak corresponding to CO bound to Ti^4+^ cations of TiO_2_ (η^1^-CO_Ti_*; ν(C–O) = 2194 cm^–1^).^113^

Additionally, multiple forms of carbonate species appear (monodentate, η^1^-CO_3_*_Ti_ [ν_s_(C–O) = 1300–1420 cm^–1^]; bidentate, η^2^-CO_3_*_Ti_ [ν_a_(C–O) = 1550–1610 cm^–1^])^113−118^ along with features that signify the presence of water molecules (δ(H–O–H) = 1600–1630 cm^–1^; ν(O–H) = 3460–3630 cm^–1^)^113,116^ and surface hydroxyl groups on TiO_2_ (ν(O–H) = 3630–3720 cm^–1^).^113,116^ The peak area of linearly adsorbed CO on TiO_2_ (η^1^-CO*_Ti_) is negligible compared to CO bound linearly to Au atoms (η^1^-CO*_Au_), which appears prominently upon samples possessing Au nanoparticles (Section S4).

Prior studies demonstrate that carbonate species bind strongly and inhibit CO oxidation catalysis, which has been attributed to competitive adsorption of carbonate at interfacial sites present at the perimeter of Au nanoparticles.^119,120^ Thus, we hypothesize that the ratio of the peak area of η^1^-CO_Au_* (*A*_CO–Au_) to the area for to η^1^-CO_3_*_Ti_ and η^2^-CO_3_*_Ti_ features (*A*_CO_3__) scales in proportion to the ratio of the number of metallic Au sites to the number of sites at the interface between Au and TiO_2_. This ratio of features is approximately five times greater for 25 nm Au–TiO_2_ in comparison to 5 nm Au–TiO_2_, which agrees with expectations that the fraction of exposed Au atoms that bind CO grows in comparison to the fraction sites that reside at the interface between the support and Au nanoparticles as mean nanoparticle diameters increase. This conclusion agrees with geometric arguments for cuboctahedra Au nanoparticles using correlations established by Ribeiro (eq 11)^87^ and modeled by Van Hardeveld and Hartog,^88^ as shown below.
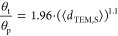
11

This analysis suggests that the fraction of Au atoms that exist at the surface of nanoparticles (θ_t_) increase relative to the fraction of Au atoms that reside at the exposed perimeter of nanoparticles (θ_p_) as the mean diameter increases, which correlates with integrated peak areas of adsorbed CO (*A*_CO–Au_/*A*_CO_3__ ∝ θ_t_/θ_p_) on different-sized nanoparticles (Figures S15 and S16). Moreover, Figure 8b shows that rate ratios of H_2_O_2_ and H_2_O formation increase with this ratio of surface Au atoms to perimeter atoms (θ_t_/θ_p_). This correlation (*R*^2^ = 0.991*a*) agrees with the hypothesis that perimeter sites at the Au–TiO_2_ interface preferentially form H_2_O, but the remaining surface Au atoms favor H_2_O_2_ formation.

### 3.4 Activation Barriers for H_2_O_2_ and H_2_O Formation Depend Differently on Active Site Motifs

The interpretation of measured ratios of product formation rates (eqs 9 and 10) depends strongly upon the fraction of Au atoms present at interfacial sites, demonstrating that the position and coordination of Au atoms within active sites impact the rate constants for OOH* conversion to H_2_O_2_ (*k*_4_) and H_2_O (*k*_5_) differently. These differences suggest that the stabilization of the associated transition states responds strongly to the proximity of the TiO_2_ surface.

Product formation rates were measured as functions of temperature to quantify the apparent activation enthalpies for H_2_O_2_ (Δ*H*_H_2_O_2__^‡^) and H_2_O (Δ*H*_H_2_O_^‡^) formation on each catalyst. Measurements were performed at equivalent conditions to facilitate meaningful comparisons across all catalysts (200 kPa H_2_, 60 kPa O_2_, 278–308 K), which corresponds to a kinetic regime where rates increase in proportion to [H_2_] and do not vary with [O_2_]. Representative time on stream measurement used for calculating activation enthalpies are reported in Figure S17.

**Figure 9:**
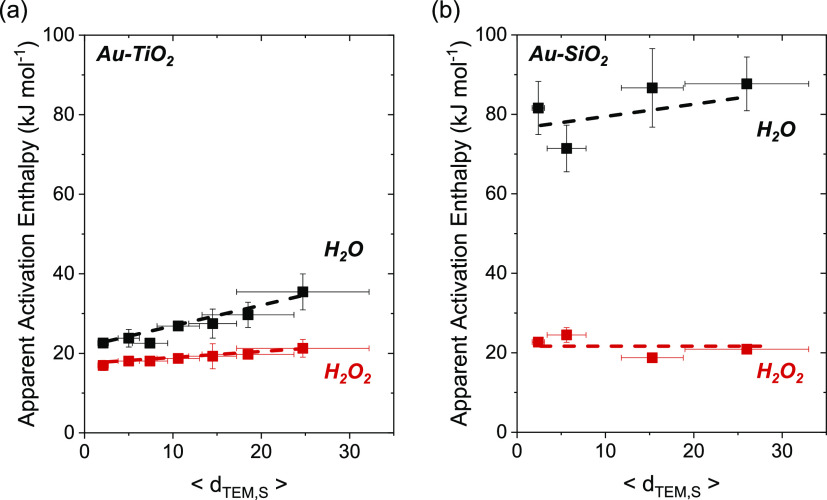
Apparent activation enthalpies of H_2_O_2_ (Δ*H*_H_2_O_2__^‡^, red ■) and H_2_O (Δ*H*_H_2_O_^‡^, ■) formation as functions of mean Au nanoparticle diameter on (a) Au–TiO_2_, and (b) Au–SiO_2_ materials (200 kPa H_2_, 60 kPa O_2_, 278–308 K). Figures S18 and S19 show corresponding steady-state rate measurements as a function of temperature. Dashed lines intended to guide the eyes.

Figure 9a,b show that values of Δ*H*_H_2_O_^‡^ increase with the mean diameters of Au nanoparticles (2–25 nm) on both TiO_2_ and SiO_2_, whereas Δ*H*_H_2_O_2__^‡^ varies only slightly. Both Au–SiO_2_ and Au–TiO_2_ present relatively low Δ*H*_H_2_O_2__^‡^ that span a narrow range (16–22 kJ mol^–1^). Values for Δ*H*_H_2_O_^‡^ reflect intrinsic barriers of cleaving O–O bonds within OOH* species, which occur with greater barriers upon Au–SiO_2_ (72–85 kJ mol^–1^) than on Au–TiO_2_ (22–35 kJ mol^–1^). These observations agree with the results in Figure 2 and show that large Au nanoparticles and Au nanoparticles on SiO_2_ more selectively form H_2_O_2_ than small nanoparticles or those with a significant fraction of Au atoms present at interfaces with TiO_2_. The origins of these differences must arise from distinct site requirements for transition states that activate H_2_ and cleave H–H bonds and those that either reduce OOH* to H_2_O_2_ or break O–O bonds in OOH* to form H_2_O (e.g., steps 1a, 4, and 5).

These distinct site requirements appear most clearly by simultaneously examining activation enthalpies for H_2_ activation (Δ*H*_H_2__^‡^, determined from measurements of −*r*_H_2__) and the differences between barriers to form H_2_O_2_ and H_2_O (ΔΔ*H*^‡^ = Δ*H*_H_2_O_^‡^ – Δ*H*_H_2_O_2__^‡^). Applying Eyring theory to eq 2 expands the rate of hydrogen consumption as
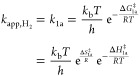
12where the apparent rate constant of H_2_ activation (*k*_app,H_2__) depends on the Boltzmann constant (*k*_b_), Planck constant (*h*), and activation Gibbs free energy (Δ*G*_1a_^‡^), entropy (Δ*S*_1a_^‡^), and enthalpy (Δ*H*_1a_^‡^ = Δ*H*_H_2__^‡^) for H_2_ consumption. The value of Δ*H*_H_2__^‡^ appears directly in expressions for the rates of product formation and the terms Δ*H*_H_2_O_2__^‡^ and Δ*H*_H_2_O_^‡^ (derived in Section S5), because H_2_ activation represents the kinetically relevant step in the sequence of steps that form both products. While the molecular interpretation of absolute values for Δ*H*_H_2_O_2__^‡^ and Δ*H*_H_2_O_^‡^ remain elusive, analysis of the ratios of H_2_O_2_ and H_2_O formation rates provides molecularly interpretable quantities. Combining these terms with the structures proposed by transition state theory gives the relationship.
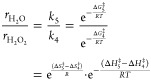
13where the differences between the intrinsic activation free energies of steps 4 (Δ*G*_4_^‡^) and 5 (Δ*G*_5_^‡^) represent the differences between apparent barriers of H_2_O_2_ and H_2_O formation (e.g., ΔΔ*H*^‡^ = Δ*H*_H_2_O_^‡^ – Δ*H*_H_2_O_2__^‡^ = Δ*H*_5_^‡^ – Δ*H*_4_^‡^) and their entropic contributions (Δ*S*_5_^‡^,Δ*S*_4_^‡^). These analyses allow comparisons of Δ*H*_H_2__^‡^and ΔΔ*H*^‡^ across catalysts with different Au nanoparticle diameters and support identities, providing molecular insight into how distinct structural motifs impact the transition states of H–H and O–O bond rupture during the formation of H_2_O_2_ or H_2_O.

Values of Δ*H*_H_2__^‡^ increase from 21 to 29 kJ mol^–1^ on Au–TiO_2_ as mean Au nanoparticle diameters increase from 2 to 25 nm (Figure S20), while Δ*H*_H_2__^‡^ remains equal to ∼35 kJ mol^–1^ on all Au–SiO_2_ catalysts. Note that these values are consistent with or slightly lower than barriers of H_2_ activation reported in the gas phase (∼25 to ∼45 kJ mol^–1^) on Au–TiO_2_ materials.^30,93^ In comparison, ΔΔ*H*^‡^ values span a much greater range of values and increase from 6 to 14 kJ mol^–1^ on Au–TiO_2_ and 59 to 66 kJ mol^–1^ on Au–SiO_2_ across the same range of Au nanoparticle diameters (2–25 nm). These observations suggest that intrinsic barriers to activate H–H bonds is less sensitive to its active site than the activation of O–O bonds. Additionally, these trends indicate metallic Au atoms that exist furthest from reducible or Lewis acidic oxide supports present the greatest barriers for activating O–O bonds and slightly higher barriers for activating H_2_, leading to greater apparent barriers for all pathways on the largest Au nanoparticles and most refractory supports (e.g., SiO_2_).

Interfaces between Au atoms and TiO_2_ present significantly lower barriers for dissociating dioxygen surface intermediates and modestly lower barriers for activating H_2_. Consequently, the smallest Au nanoparticles upon TiO_2_ give the greatest rates for H_2_ consumption and H_2_O formation among all Au–TiO_2_ and Au–SiO_2_ catalysts examined. These conclusions agree with computational studies that showed Au–TiO_2_ interfaces (e.g., Au–TiO_2_) bind dioxygen more exothermically than metallic Au surfaces (Δ*E*_ads_^Au–TiO_2_^ = −0.82–1.01 eV, Δ*E*_ads_^Au^ = −0.55 eV) and present lower barriers of dissociating O–O bonds (*E*_a_^A–TiO_2_^ = 0.42–0.6 eV, *E*_a_^Au^ = 1.59 eV).^21−23^ The poor activation of O_2_ on metallic Au surfaces is consistent with its largely occupied d-states that disfavor the binding of most adsorbates.^100^ However, Yates and Neurock suggested that the Au–TiO_2_ interface enables the adsorption of O_2_ in a di-σ configuration.^21^ Moreover, Au atoms transfer electron density to Ti atoms at these interfaces, strengthening the binding of O_2_ at these sites.^22^ These differences in electronic structure may favor the back-donation of electrons from Au to the π* antibonding orbitals of O–O bonds,^90^ consistent with lower barriers of O–O bond dissociation at Au–support interfaces.

These insights agree with the large barriers for O–O bond dissociation and high H_2_O_2_ selectivities (>90%) during electrochemical ORR on polycrystalline Au foils, which do not possess interfacial sites.^67,69^ Thus, metallic Au and Au–SiO_2_ interfaces favor transition states of oxygen reduction that stabilize O–O bonds more effectively than Au–TiO_2_.

**Figure 10:**
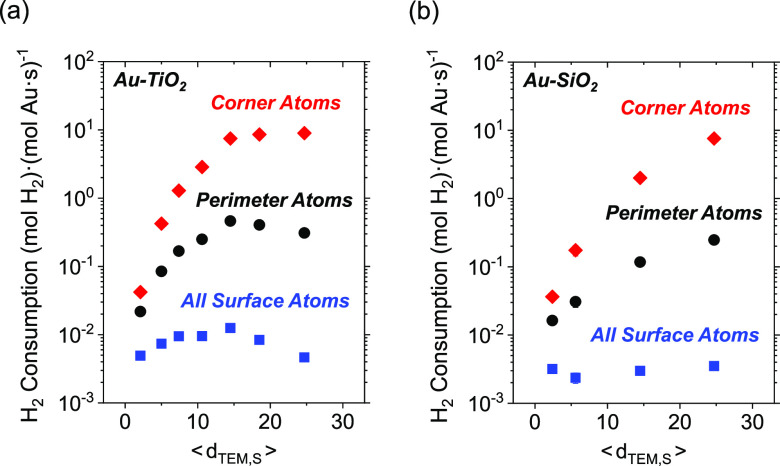
Steady-state rates of H_2_ consumption as a function of the surface-area normalized Au nanoparticle diameter on (a) Au–TiO_2_ and (b) Au–SiO_2_ materials (200 kPa H_2_, 60 kPa O_2_, 278 K). Rates are normalized by the total number of surface atoms (blue ■), perimeter atoms (●), and corner atoms (red ◆), estimated using correlations reported by Ribeiro and co-workers.^87,88^

We sought to elucidate the active site motifs that favor H_2_ activation and the formation of H_2_O_2_ or H_2_O by comparing activation barriers and quantifiable structural descriptors for the supported Au catalysts. Estimates for turnover rates for H_2_ consumption (−*r*_H_2__ = *r*_H_2_O_ + *r*_H_2_O_2__) were calculated by normalizing measured rates (Figure 2) by approximated numbers for surface, perimeter, or corner atoms, which were determined from the mean Au nanoparticle diameters and using correlations established by Ribeiro^87^ and modeled by Van Hardeveld and Hartog^88^ for a cuboctahedra geometry. Figure 10 shows that H_2_ consumption turnover rates calculated using the total number of surface Au atoms remain mostly constant across Au nanoparticles of all diameters on SiO_2_ and vary only slightly among Au–TiO_2_. In contrast, turnover rates normalized by the number of perimeter or corner sites vary by two to 3 orders of magnitude with changes in the mean diameter of Au nanoparticles.

Taken together, the small span of Δ*H*_H_2__^‡^ values (21–35 kJ mol^–1^) across Au–TiO_2_ and Au–SiO_2_ catalysts (Figure S20) and H_2_ consumption rates that vary by a factor of 3 or less (when normalized by estimates for the total number of exposed Au atoms; Figure 10) indicate that H_2_ activation weakly senses differences between the different forms of active sites formed with Au atoms when the reaction proceeds by heterolytic processes within liquid water. In comparison, the wide span of ΔΔ*H*^‡^ values (6–66 kJ mol^–1^) across the same Au–SiO_2_ and Au–TiO_2_ materials demonstrate that dissociative reactions of dioxygen surface intermediates (e.g., OOH*) strongly depend on proximity to the Au–support interface (Figure S20). Thus, sites at the Au–support interface preferentially form H_2_O, while sites far from the interface yield H_2_O_2_. These findings expand the understanding of the distinct site requirements of reactions of H_2_ and O_2_, which previously attributed all of the catalysis to sites at the perimeter of Au nanoparticles.^29,30^ Comparisons to prior studies suggest that solvation of Au nanoparticles by liquid water suppresses the structure sensitivity of H_2_ activation by enabling H_2_ dissociation across the entire catalyst surface.

**Figure 11:**
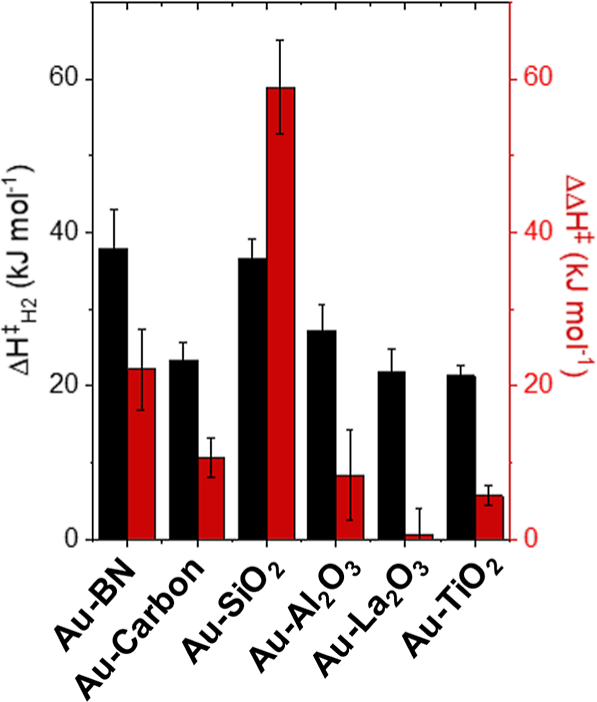
Apparent activation enthalpies of hydrogen consumption (Δ*H*_H_2__^‡^; black) and differences between activation enthalpies of H_2_O_2_ and H_2_O formation (ΔΔ*H*^‡^; red) on 2–3 nm Au nanoparticles supported on BN, carbon, SiO_2_, Al_2_O_3_, La_2_O_3_, and TiO_2_ (200 kPa H_2_, 60 kPa O_2_, 278 K). Figure S23 shows associated steady-state rate measurements versus temperature.

The generality of these interpretations was tested by comparing rates and kinetic barriers for H_2_ consumption, H_2_O_2_ formation, and H_2_O production upon 2–3 nm Au nanoparticles supported on six distinct supports. Figure 11 shows values of Δ*H*_H_2__^‡^ and ΔΔ*H*^‡^ obtained from Au nanoparticles supported on BN, carbon, SiO_2_, Al_2_O_3_, La_2_O_3_, and TiO_2_, all measured within a consistent kinetic regime (vide supra; Figure S11). Moreover, materials with the greatest values of ΔΔ*H*^‡^ present greater barriers for primary H_2_O_2_ decomposition (i.e., Δ*H*_8_^‡^), as determined from reactions among H_2_O_2_ and H_2_ (200 kPa H_2_, 0.2 mM H_2_O_2_, 278–308 K; Figure S22). Thus, catalysts capable of dissociating O–O bonds sourced from O_2_ are similarly effective at dissociating H_2_O_2_.

Generally, metal oxide supports show lower values of Δ*H*_H_2__^‡^ compared to BN, while carbon presents barriers similar to those measured upon metal oxides. We hypothesize that nucleophilic or Brønsted basic functions may aid the activation of H–H bonds by accepting a proton at the Au–support interface, as suggested elsewhere.^29,30^ Still, the presence of water molecules extends the range of catalysis to all surface Au atoms by facilitating these proton transfer steps (vide supra). Thus, the lower rates of H_2_ activation on carbon and BN may result from their high level of hydrophobicity, which may lower the concentration of interfacial water molecules that can solvate Au surfaces and disrupt proton transfer steps.

The dissociation of O–O bonds show a much stronger dependence upon the chemical function and identity of the support material. Reducible (e.g., TiO_2_, La_2_O_3_)^121^ and Lewis acidic (e.g., TiO_2_, La_2_O_3_, Al_2_O_3_)^122,123^ metal oxides show lower values for ΔΔ*H*^‡^. Thus, we hypothesize that the presence of oxygen vacancies or Lewis acidic metal centers of these Au–support interfaces more readily enable the formation of strongly bound superoxide species (e.g., Au–O–O–M) or related species, which may present lower barriers of O–O dissociation than on more refractory materials (vide supra). By comparison, the Au–SiO_2_ interface is the most refractory of these catalysts and may bind O_2_ so weakly such that the majority of H_2_ and O_2_ catalysis occurs upon the metallic surfaces of Au nanoparticles rather than the perimeter sites of Au–SiO_2_ (Figure 9). Finally, the refractory and hydrophobic materials (e.g., BN, Carbon) show intermediate values of ΔΔ*H*^‡^, which may suggest that the unfavorable interaction of water near these supports may lower the interfacial concentration of water molecules and destabilize O–O bonds relative to hydrophilic and refractory supports like silica. Furthermore, these properties and the identity of the support significantly impact rates and selectivities of H_2_O_2_ formation using dilute PdAu_*x*_ nanoparticle catalysts (Figures S24–S28).

## 4 Conclusions

The interface between Au nanoparticles and supports gives rise to active site motifs that facilitate the reduction and dissociation of dioxygen species; however, the activation of H_2_ only weakly senses the presence of these interfaces in liquid water. This knowledge expands the understanding from prior work studying reactions of H_2_ and O_2_ over supported Au nanoparticles within dry or dilute vapor pressures of water (10^–4^ to 0.1 kPa H_2_O). These reports showed that sites at the perimeter of Au–support interfaces catalyze heterolytic reactions that activate H–H and O–O bonds, but water molecules introduce distinct proton-transfer reactions at these interfaces that accelerate O_2_ activation and inhibit H_2_ activation. However, we demonstrate that condensed water (55.5 M H_2_O) further modifies the structure sensitivity of this reaction network by stabilizing O–O bonds, allowing the formation of H_2_O_2_, and enabling all surface Au atoms to catalyze heterolytic reactions with H_2_ at the metal–liquid–support interface. These insights and the accompanying understanding of manipulating relative rates of H_2_ and O_2_ activation carry broad significance due to the ubiquity of these reactants (H_2_, O_2_, and H_2_O) in reactions using Au catalysts (e.g., hydrogenation, partial oxidations, electrocatalysis).

Kinetic analyses of H_2_ and O_2_ activation combined with isotopic measurements show that Au catalysts follow a similar mechanism. Specifically, proton acceptors (e.g., H_2_O, M–OH) cocatalyze the kinetically relevant activation of H–H bonds at the interface of Au nanoparticles, liquid water, and the support. Consequently, rates of H_2_ consumption are 1–2 orders of magnitude greater on Au–MO_X_ interfaces than on refractory and hydrophobic interfaces (e.g., carbon, BN). Yet, comparisons of barriers and rates normalized by the total number of sites at the surface, perimeter, and corner sites of Au nanoparticles show that H–H activation is mostly insensitive to the mean diameter of Au nanoparticles, indicating that all surface atoms may heterolytically activate H–H bonds with liquid water molecules. However, O–O bond dissociation is much more structure-sensitive. More reducible or Lewis acidic metal oxides (e.g., Au–LaO_2_) favor the dissociation of O–O bonds compared to more inert supports like Au–SiO_2_, which stabilize O–O bonds. Moreover, increasing the size of Au nanoparticles decreases the fraction of Au atoms at the metal–support interface versus metallic Au atoms far from the support, decreasing the proportion of sites that cleave O–O bonds.

This understanding provides multiple strategies to manipulate the activation of H_2_- and O_2_-derived species at metal–liquid–support interfaces. These findings may guide the design of catalytic materials for thermochemical redox reactions within liquid water (e.g., alcohol oxidation, HCOOH decomposition, and so forth). Similarly, these principles provide structure–function relationships to influence heterolytic steps relevant to electrochemical reactions in aqueous electrolyte. Furthermore, future work should further elucidate how the interfaces of water, metal nanoparticles, and metal oxides affect heterolytic paths during the reduction and oxidation of small molecules and organic substrates.

## Supporting Information Available

Additional Characterization of materials by TEM, ICP-OES, EDXRF, and point of zero charge analysis; process flow diagrams of reactors, additional pressure dependence data, data for kinetic isotope measurements, discussion of rate derivations, isotherms from FTIR spectra of adsorbed CO, additional discussion of Eyring theory and relevant Arrhenius plots, and rate data normalized by different site counts (PDF)
