# Supplementary material for: Distinct Site Motifs Activate O2 and H2 on Supported Au Nanoparticles in Liquid Water
Source: ACS Catal. 2024 Feb 15;14(5):3248–65. doi: 10.1021/acscatal.3c05072 (PMC10913054; doi:10.1021/acscatal.3c05072)
Supplement: Supplementary file 1 — cs3c05072_si_001.pdf [file cs3c05072_si_001.pdf]

Supporting Information

Distinct Site Motifs Activate O_2 and H_2 on Supported Au Nanoparticles in Liquid Water

Jason S. Adams,[1,†] Haoyu Chen,[1,2] Tomas Ricciardulli,[1,†] Sucharita Vijayaraghavan,[1,2] Abinaya Sampath,[1,†] David W. Flaherty[1,2*]

[1]Department of Chemical and Biomolecular Engineering, University of Illinois at Urbana-Champaign, IL, 61801, USA
[2]School of Chemical and Biomolecular Engineering, Georgia Institute of Technology, GA, 30332, USA

*To whom correspondence should be addressed. Email: dflaherty3@gatech.edu

† - Present address differs from listed affiliation.

Section S1: Supplemental Material Characterization and Experimental Methods

Table S1. Characterization of supported Au nanoparticle diameters from TEM histograms ($d_{TEM,S}$ and $d_{TEM,N}$) shown in Figure S1 and Au metal content from elemental analysis calculated by ICP and EDXRF. Reported materials were prepared by strong electrostatic adsorption (SEA), deposition precipitation (DP), and incipient wetness impregnation (IWI) methods followed by temperature treatments, detailed in Section 2.1 of the main text.

Preparation of material	$d_{TEM,S}$ (nm)	$d_{TEM,N}$ (nm)	ICP Au metal content (%)	EDXRF Au metal content (%)
Au/SiO_2 SEA (O_2 at 573 K 4 h)	5.6 ± 2.2	3.3 ± 2.2	0.79	1.30
Au/SiO_2 SEA (O_2 at 673 K 4 h)	15.3 ± 3.5	13.3 ± 3.5	6.09	11.88
Au/SiO_2 SEA (O_2 at 1073 K 4 h)	26.0 ± 7.0	21.4 ± 7.0	6.12	11.55
Au/SiO_2 DP (H_2 at 473 K 4 h)	2.4 ± 0.7	2.1 ± 0.7	0.46	0.82
Au/TiO_2 SEA (O_2 at 573 K 4 h)	5.0 ± 1.2	4.8 ± 1.2	2.06	2.76
Au/TiO_2 SEA (O_2 at 773 K 4 h)	7.4 ± 2.0	7.4 ± 2.0	2.05	2.76
Au/TiO_2 SEA (O_2 at 823 K 4 h)	10.6 ± 2.4	9.9 ± 2.4	2.09	2.79
Au/TiO_2 SEA (O_2 at 873 K 4 h)	14.5 ± 2.7	13.9 ± 2.7	2.09	2.77
Au/TiO_2 SEA (O_2 at 973 K 4 h)	18.5 ± 5.2	15.4 ± 5.2	2.06	2.74
Au/TiO_2 SEA (O_2 at 1073 K 4 h)	24.7 ± 7.5	19.1 ± 7.5	2.08	2.73
Au/TiO_2 DP (H_2 at 473 K 4 h)	2.1 ± 0.6	1.9 ± 0.6	2.47	3.35
Au/La_2O_3 DP (H_2 at 473 K 4 h)	3.0 ± 0.7	2.8 ± 0.7	2.30	3.62
Au/Al_2O_3 DP (H_2 at 473 K 4 h)	2.5 ± 0.6	2.2 ± 0.6	2.87	5.20
Au/Carbon IWI (H_2 at 473 K 4 h)	2.2 ± 0.8	1.6 ± 0.8	2.81	5.62
Au/BN IWI (H_2 at 473 K 4 h)	2.2 ± 0.5	1.9 ± 0.6	3.60	4.56

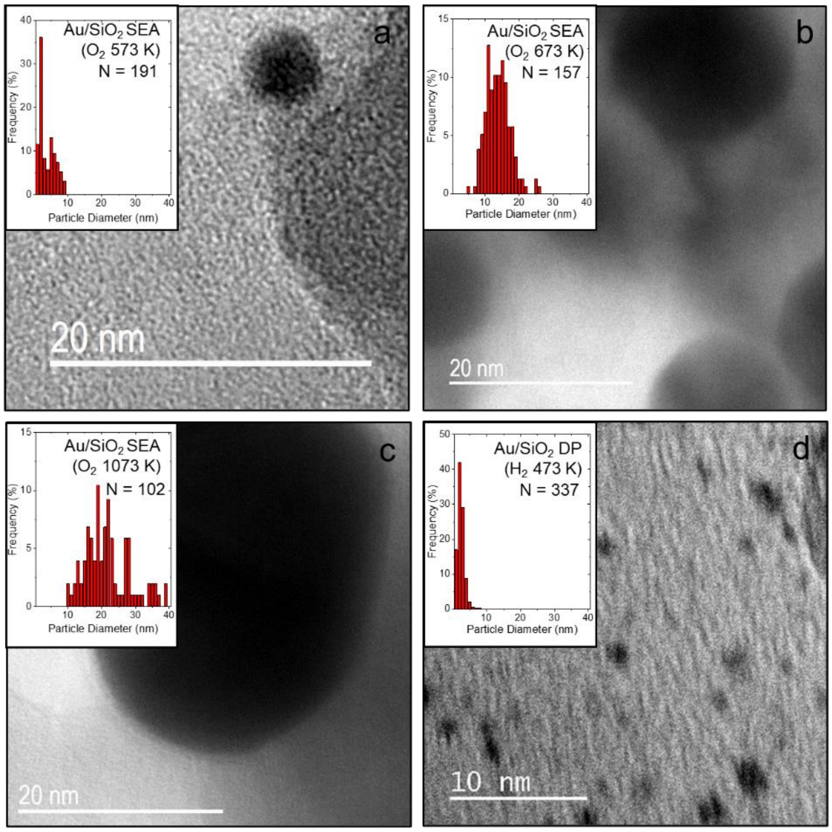

Figure S1.i. Representative TEM image of Au-SiO_2 materials prepared by strong electrostatic adsorption (SEA) and oxidized at (a) 573 K, (b) 673 K, and (c) 1073K or by precipitation (DP) and reduced at 473 K. Images of Au nanoparticles contain an inset histogram of the particle size distribution. More than 100 particles were measured to calculate the values of $d_{TEM,S}$ and $d_{TEM,N}$, as reported in Table S1 as the surface area-averaged and average diameters, respectively.

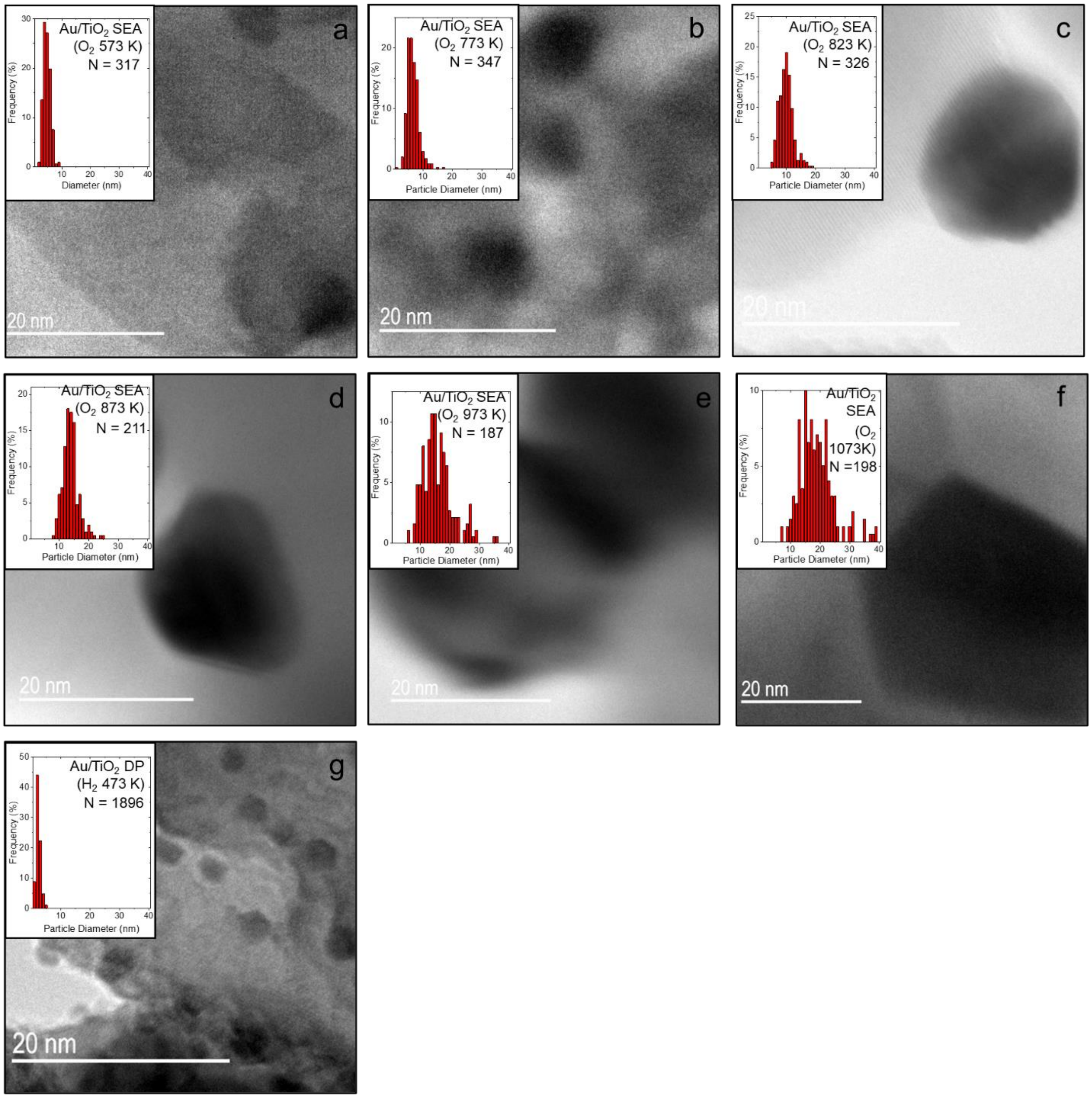

Figure S1.ii. Representative TEM image of Au-TiO_2 materials prepared by strong electrostatic adsorption (SEA) and oxidized at (a) 573 K, (b) 773 K, (c) 823 K, (d) 873 K, (e) 973 K, and (f) 1073 K or (g) prepared by deposition precipitation (DP) and reduced at 473 K. Images of Au nanoparticles contain an inset histogram of the particle size distribution. More than 100 particles were measured to calculate the values of $d_{TEM,S}$ and $d_{TEM,N}$, as reported in Table S1 as the surface area-averaged and average diameters, respectively.

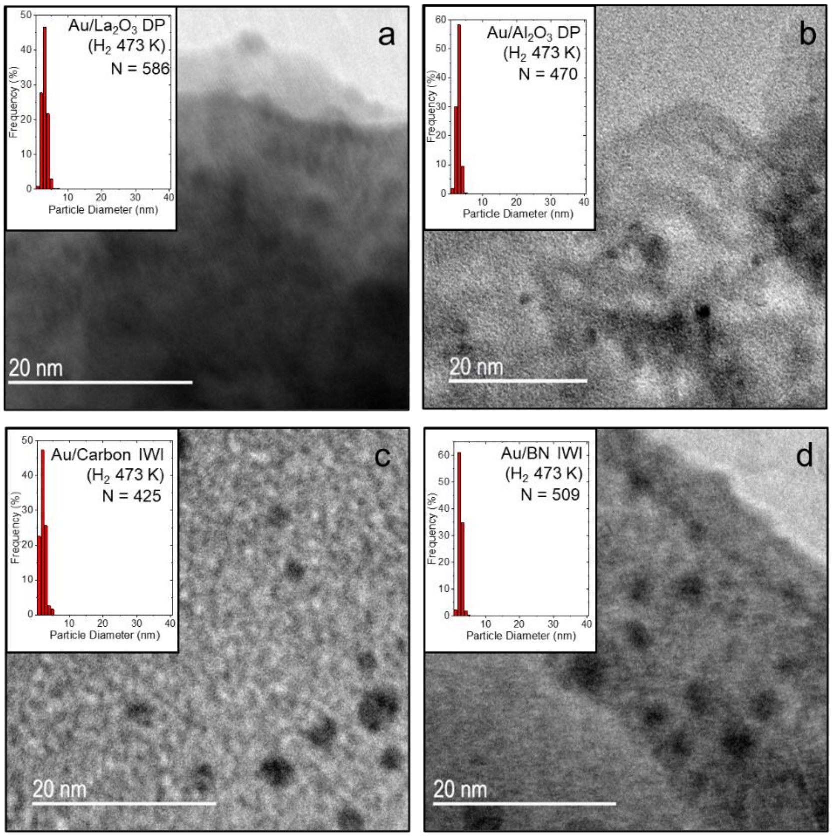

Figure S1.iii. Representative TEM image of other materials prepared by deposition precipitation (DP) and reduced at 473 K on (a) La_2O_3, (b) Al_2O_3, (c) SiO_2 or by incipient wetness impregnation (IWI) and reduced at 473 K on (d) carbon and (e) BN. Images of Au nanoparticles contain an inset histogram of the particle size distribution. More than 100 particles were measured to calculate the values of $d_{TEM,S}$ and $d_{TEM,N}$, as reported in Table S1 as the surface area-averaged and average diameters, respectively.

Figure S1 shows TEM images of the Au nanoparticle catalysts used in this study. Table S1 reports their nanoparticle diameters and other characterized properties. Generally, materials prepared by strong electrostatic adsorption gave larger-sized Au nanoparticles than those of precipitation deposition and incipient wetness impregnation. The identity of the support has a weak impact on the size of Au nanoparticles for all samples reduced at 473 K. Furthermore, as materials were calcined at increasing temperatures, they tended to increase in size and polydispersity.

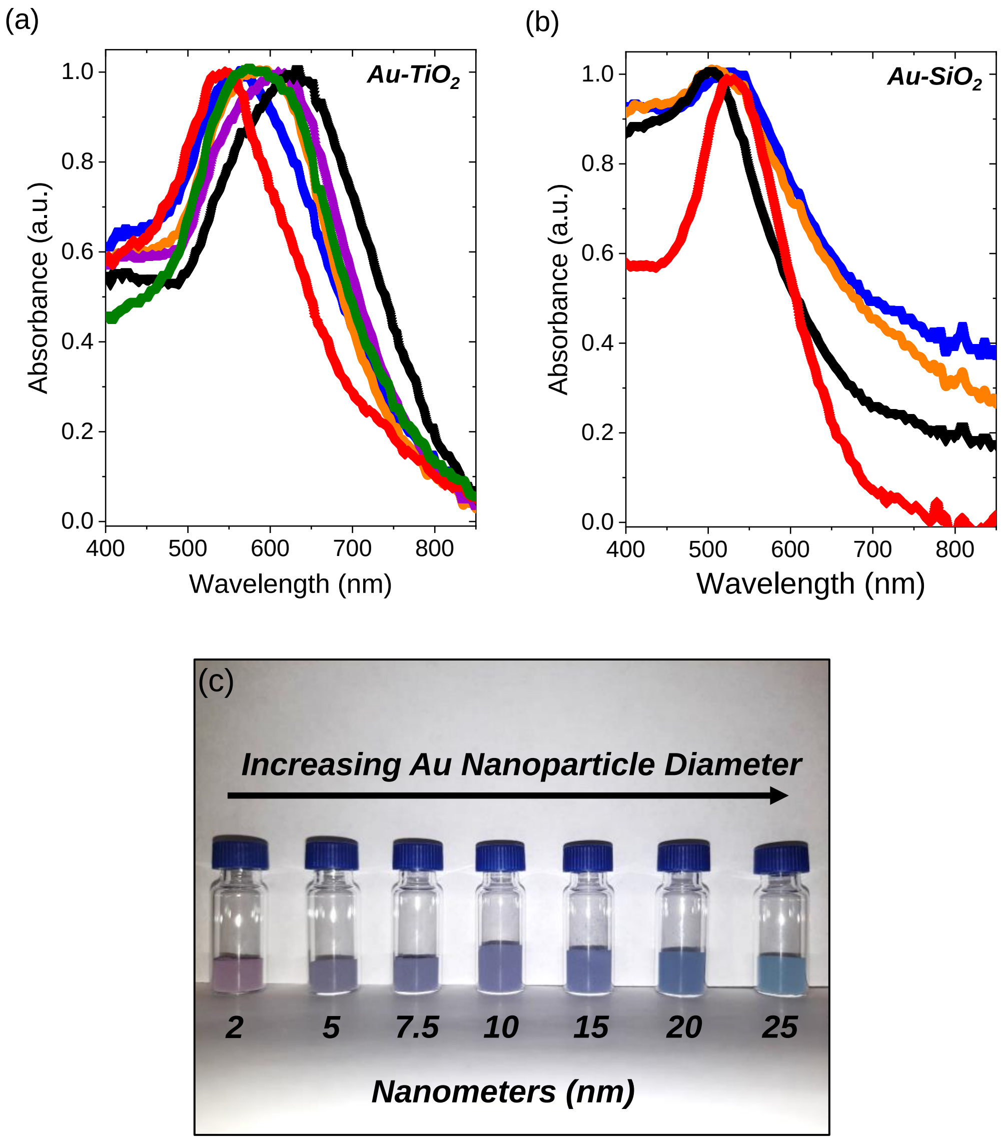

Figure S2. DRUV-vis spectra of (a) TiO_2-supported and (b) SiO_2-supported Au nanoparticles collected *ex situ* in ambient air at 298 K. Different colors indicate 2 nm (red), 5 nm (blue), 7.5 nm (brown), 10 nm (green), 15 nm (orange), 20 nm (purple), and 25 nm (black) Au nanoparticles. (c) A photo showing the color of Au-TiO_2 materials as mean nanoparticle diameters increase from left to right.

Figures S2a and S2b show diffuse reflectance UV-vis (DRUV-vis) spectra of TiO_2-supported and SiO_2-supported Au nanoparticles calcined at different temperatures. The peak intensity of Au-TiO_2 materials shifts to greater wavelengths as materials were subjected to greater calcination temperatures, consistent with larger-size Au nanoparticles. Similar trends are observed on Au-SiO_2 materials but are less pronounced. Such changes in DRUV-Vis spectra agree with qualitative changes in the color of Au-TiO_2, changing from pink to blue with larger nanoparticles.

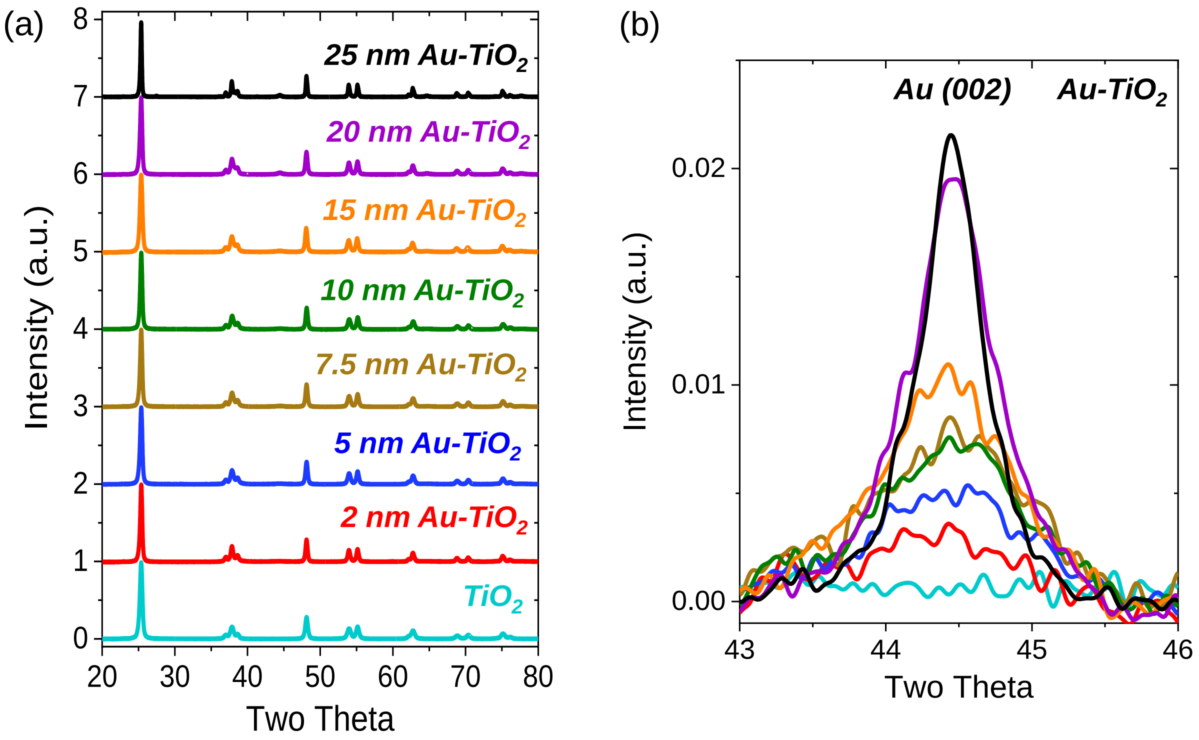

Figure S3. X-ray diffractograms of TiO_2-supported Au nanoparticles collected *ex situ* in ambient air at 298 K between (a) 2θ = 20-80° and (b) 2θ = 43-46°. Different colors indicate 2 nm (red), 5 nm (blue), 7.5 nm (brown), 10 nm (green), 15 nm (orange), 20 nm (purple), and 25 nm (black) Au nanoparticles on TiO_2 and the TiO_2 support (cyan).

Figure S3 shows X-ray diffractograms of TiO_2-supported Au nanoparticles, interpreted by equation S1.1

$$< d_{XRD} > = \frac{K\lambda}{\beta\cos(\theta)} \quad \text{(S1.1)}$$

where the average diameter of a nanoparticle crystallite ($< d_{XRD} >$) depends on the X-ray wavelength of the Cu-Kα radiation source (λ = 0.15418 nm) at ambient conditions, the full-width half maximum (FWHM) of a given peak (β), the peak's Bragg angle (θ), and the shape factor (K) for octahedral nanoparticles.[1]

Figure S3a shows that the intensity and FWHM values of diffraction peaks for TiO_2 are nearly identical to the parent TiO_2 sample, indicating that the anatase phase of TiO_2 does not change upon the addition of Au and subsequent thermal treatments. Notably, the TiO_2 likely does not change since it was already pre-treated at 873 K for 4 h before adding Au atoms. Figure S3b shows that the FWHM of the (002) facet of Au nanoparticles decreases with increasing thermal treatments, consistent with changes in the size of Au nanoparticles in Table S1 following fits of the Scherrer equation (equation S1.1). Notably, the intensity of the (111) facets also increases but overlaps with other features associated with TiO_2. Thus, the analysis of these data focused on the (002) facet.

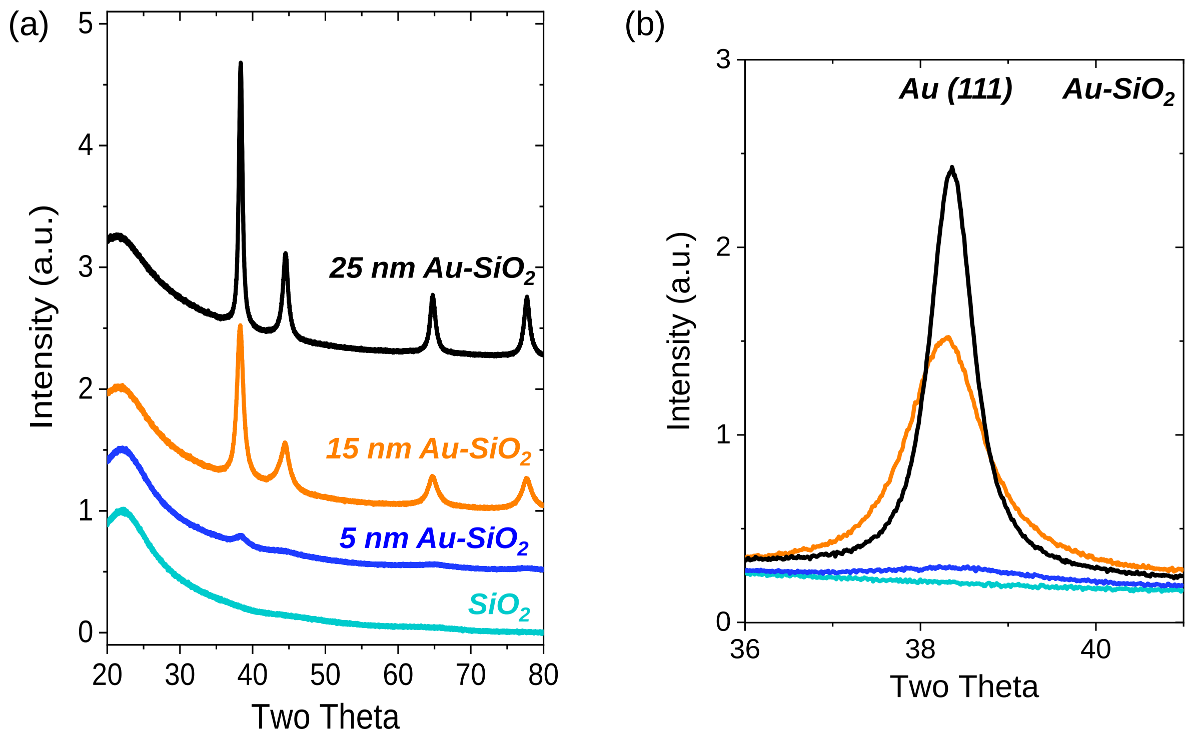

Figure S4. X-ray diffractograms of SiO_2-supported Au nanoparticles collected *ex situ* in ambient air at 298 K between (a) 2θ = 20-80° and (b) 2θ = 36-41°. Different colors indicate 5 nm (blue), 15 nm (orange), and 25 nm (black) Au nanoparticles on SiO_2 and the SiO_2 support (cyan).

Figure S4 shows X-ray diffractograms of SiO_2-supported Au nanoparticles. Figure S4a shows that the amorphous SiO_2 support is nearly identical to the parent SiO_2 sample following all thermal treatments. This lack of crystallinity of the support and greater weight loadings of Au within these samples contributes to a more distinguishable difference in diffractograms compared to the Au-TiO_2 samples in Figure S3. Moreover, the 25- and 15-nm samples of Au-SiO_2 have much greater metal loadings than the 5-nm samples, leading to even more distinguishable profiles. Figure S4b shows that the FWHM of the (111) facet of Au nanoparticles also decreases with increasing thermal treatments, consistent with changes in the size of Au nanoparticles in Table S1 following fits of the Scherrer equation (equation S1.1), as seen on Au-TiO_2.

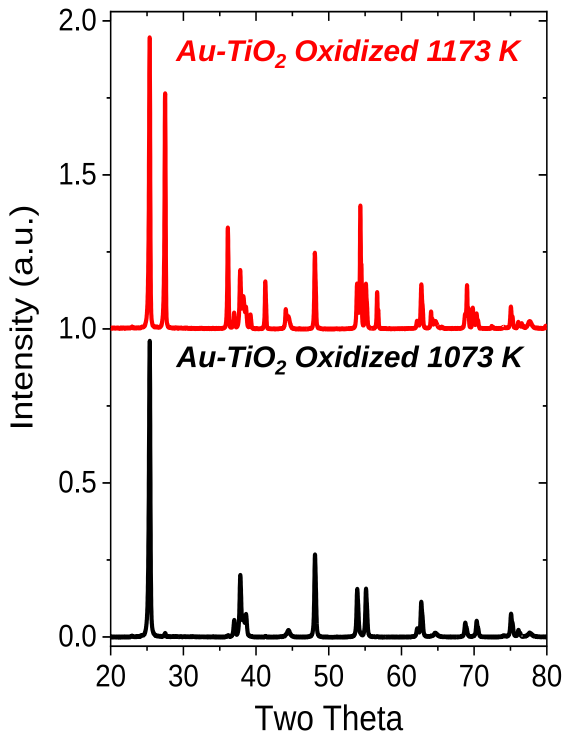

Figure S5. X-ray diffractograms of TiO_2-supported Au nanoparticles oxidized at 1078 K (black) and 1178 K (red) for 4 hr, collected *ex situ* in ambient air at 298 K between 2θ = 20-80°.

Figure S5 compares the diffractograms of TiO_2-supported Au nanoparticles when calcined at 1178 K versus 1078 K, the highest temperature treated material used for this study. The increased temperature leads to the emergence of several new peak features between 20-80°, which agree with the formation of the rutile phase of TiO_2. Therefore, samples treated at 1178 K were not used in this study to avoid convoluting the effects of using a different phase of TiO_2.

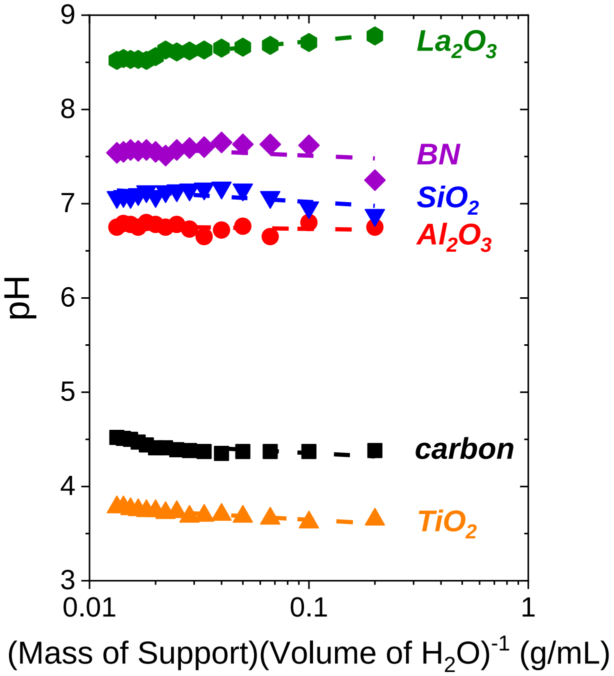

Figure S6. pH of a solution of DI H_2O as function of the mass of BN (♦), Carbon (■), SiO_2 (▼), Al_2O_3 (●), La_2O_3 (⬢), and TiO_2 (▲) added to the solution. Samples slurries were sparged in 101 kPa of He. Dashed lines represent linear fits.

Figure S6 shows the measured pH of a slurry of support material and DI H_2O sparged in He gas, in which increasing quantities of H_2O were added to the solution. These data were extrapolated to the point of incipient wetness to determine the points of zero charge for a given material.

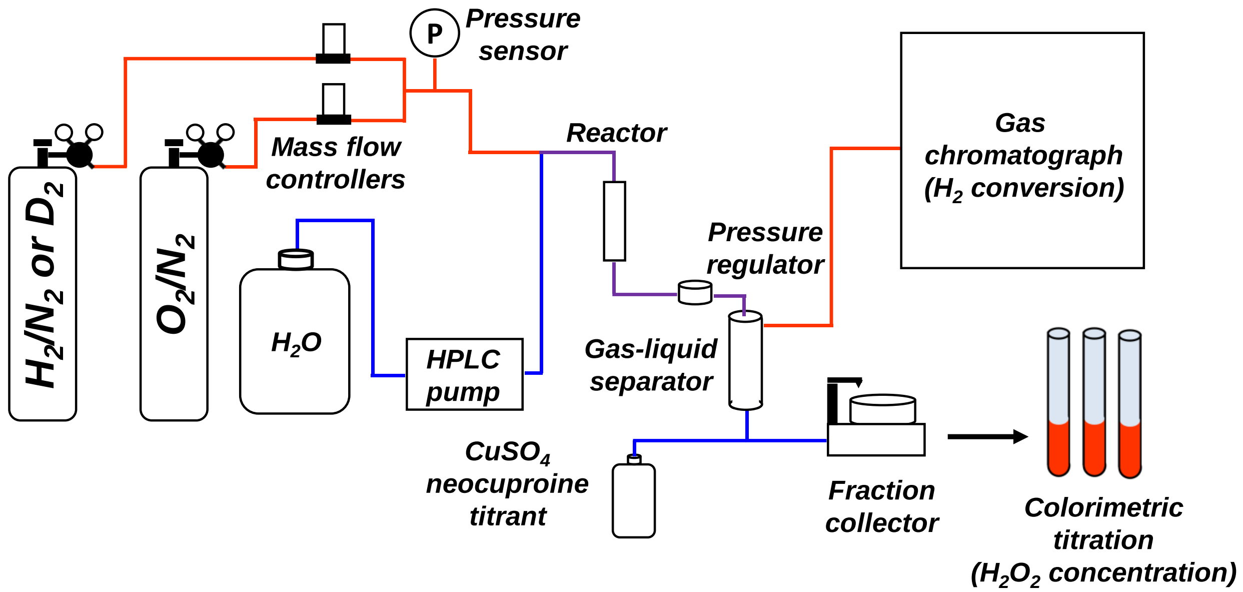

Figure S7. Process flow diagram of the trickle bed reactor used for continuous rate measurements.

Figure S7 describes the reactor system used for the steady-state rate measurements used for this study. Here, a mixture of H_2, D_2, O_2, and N_2 flow into the system from mass flow controllers, and this gas mixes with solvent pumped from the liquid carboy. This mixture was passed over the catalyst bed at the desired reactant pressure and maintained by a back-pressure regulator. The mixture was separated in a gas-liquid separator, after which the composition of each phase was analyzed to determine product compositions.

In prior work, we established that porous SiO_2 material (Davisil 646) with pellet diameters of 35-60 mesh satisfied the Madon-Boudart criterion if they maintained a rate of H_2 consumption less than $5x10^{-6}$ mol H_2 (g cat)$^{-1}$ over Pd-SiO_2 materials. However, the Au-based materials used in this study never exceeded a reactivity of $1x10^{-6}$ mol H_2 (g cat)$^{-1}$, even at the most reactive conditions (200 kPa H_2, 60 kPa O_2, 308 K). Moreover, the less reactive materials used in this study were sieved to smaller pellet diameters (80-120 mesh) than in those prior studies, indicating that the diffusion length of reacting gases is less significant in our work. Thus, the materials and conditions used here likely satisfy the Madon-Boudart criterion, provided that the diffusion coefficients reacting gasses are of a similar order of magnitude to those within Davisil 646. Such an assumption seems reasonable given that all materials used are highly porous and likely experience minimal molecular discrimination based on the specifications presented by vendors (see Section 2.1).

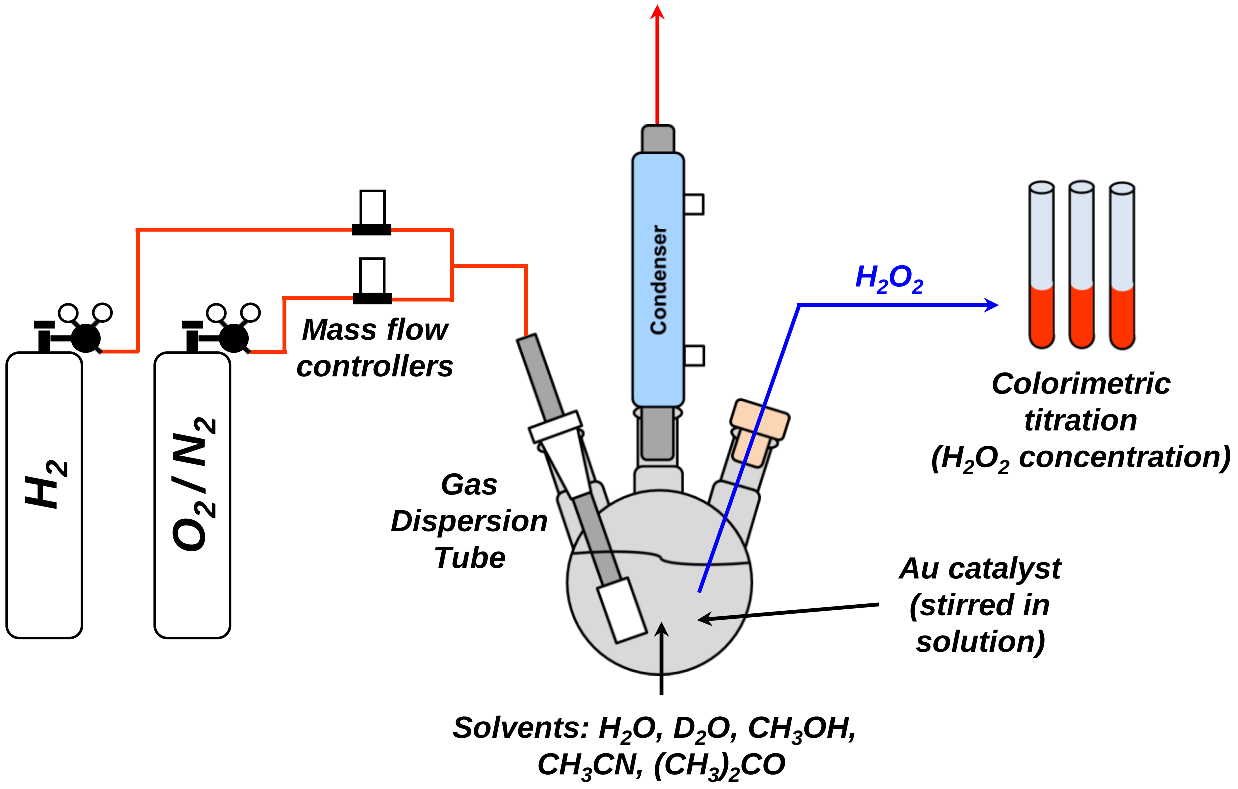

Figure S8. Process flow diagram of the semi-batch reactor set up used for the transient kinetic isotope and solvent identity measurements in this study.

Figure S8 depicts the semi-batch reactor system used to compare reaction rates within different solvents and isotopologues of water. Here, a mixture of H_2, O_2, and N_2 flow into the system from mass flow controllers and is mixed within a three-neck flask. The gas reacts with the slurry of catalyst and solvent to produce H_2O_2, which was detected by colorimetric titration.

Section S2: Supplemental Kinetic Analysis and Comparison of Supported Au Catalysts

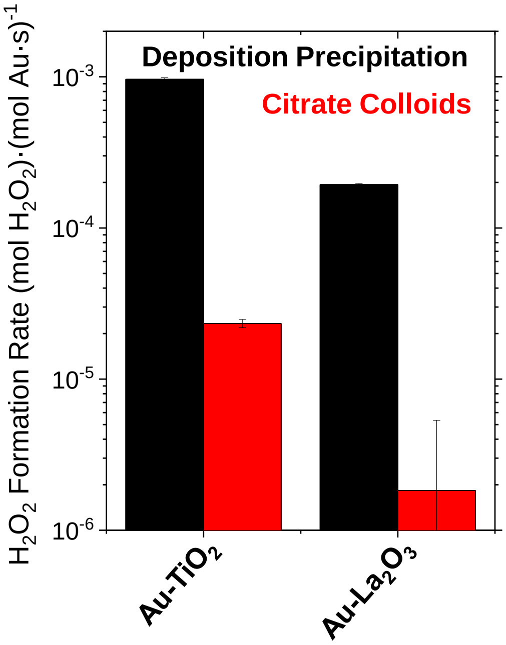

Figure S9. Comparison of steady-state H_2O_2 formation rates on ~3 nm Au nanoparticles supported on TiO_2 and La_2O_3 prepared by deposition precipitation (black) and citrate colloidal (red) techniques, as measured in a trickle-bed reactor (200 kPa H_2, 60 kPa O_2) at 278 K. Note that H_2 consumption rates were immeasurably low on colloidal samples.

Figure S9 compares the steady-state rates of H_2O_2 formation on Au-TiO_2 and Au-La_2O_3 materials prepared by deposition precipitation and colloidal techniques. The colloidal materials show 1-2 orders of magnitude lower reaction rates than those prepared by deposition precipitation, suggesting fewer catalytic active sites that activate H_2 and O_2 on the colloidal samples. The colloidal materials were calcined at 778 K, which should be sufficient to burn the associated citrate ligands and prevent poisoning. Moreover, TEM imaging of these samples suggests similar-size Au nanoparticles exist on each sample, suggesting size effects should not contribute to these differences in reactivity. Together, these findings suggest that the Au nanoparticles may not have formed a strong contact with the support, which is consistent with lower reaction rates on isolated metallic nanoparticles.

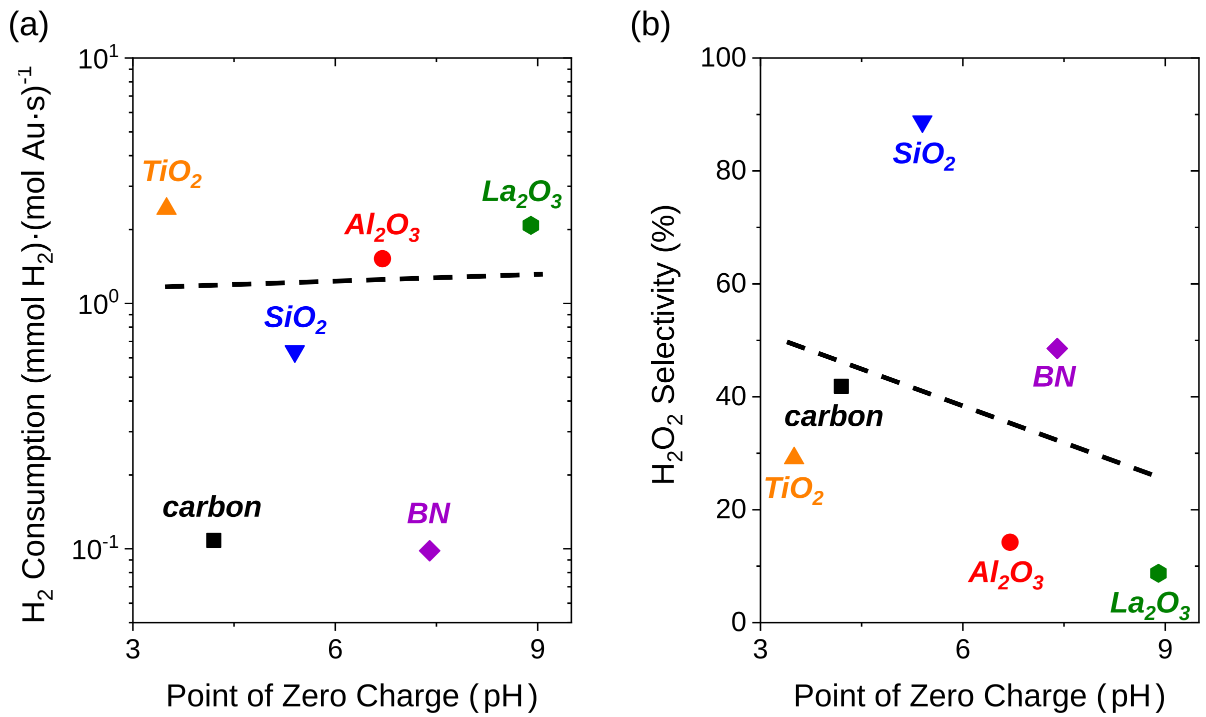

Figure S10. Steady-state (a) rates of H_2 consumption, and (b) selectivities of H_2O_2 formation over 2-3 nm Au nanoparticles supported on BN (♦), Carbon (■), SiO_2 (▼), Al_2O_3 (●), La_2O_3 (⬢), and TiO_2 (▲) in a trickle-bed reactor (200 kPa H_2, 60 kPa O_2, 278 K). Dashed lines represent linear fits.

Figure S10 shows the rates of H_2 consumption and H_2O_2 selectivity of supported Au nanoparticles as a function of the points of zero charge for support materials shown in Figure S6. Figure S10a shows that H_2 consumption does not correlate with the points of zero charge ($R^2_{r_{H_2}} = 0.0$), suggesting that local acidity has no clear impact on the activation of H–H bonds. Figure S10b shows that H_2O_2 selectivity poorly correlates with the points of zero charge ($R^2_{r_{H_2}} = 0.12$), indicating that local acidity may partially stabilize O–O bonds. Still, other descriptors of the support (e.g., oxygen vacancies, hydrophilicity) may lead to more significant differences in the apparent differences of reactivity of H_2 and O_2.

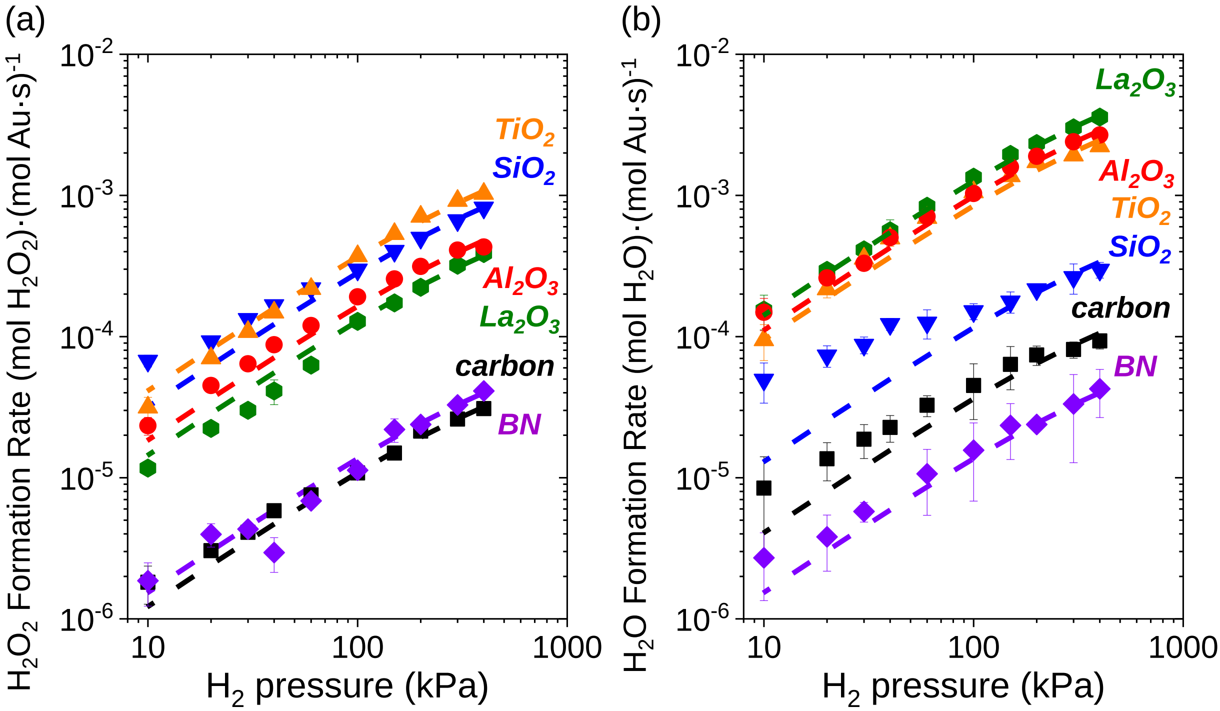

Figure S11. Steady-state H_2O_2 and H_2O formation rates on 2-3 nm Au nanoparticles supported on BN (♦), C (■), SiO_2 (▼), Al_2O_3 (●), La_2O_3 (⬢), and TiO_2 (▲) as functions of the pressure of H_2 (10-400 kPa H_2, 60 kPa O_2) and O_2 (10-400 kPa O_2, 60 kPa H_2) at 278 K. Dashed lines fitted to equations 7 and 8.

Figure S11 shows that the rates of H_2O_2 and H_2O formation increase in proportion with the pressure of H_2 on all support Au nanoparticles used for this study, suggesting a consistent mechanism. Together, these findings, combined with those of Section 3.2, support a common mechanism involving the kinetically relevant activation of H–H bonds between each Au-based catalyst.

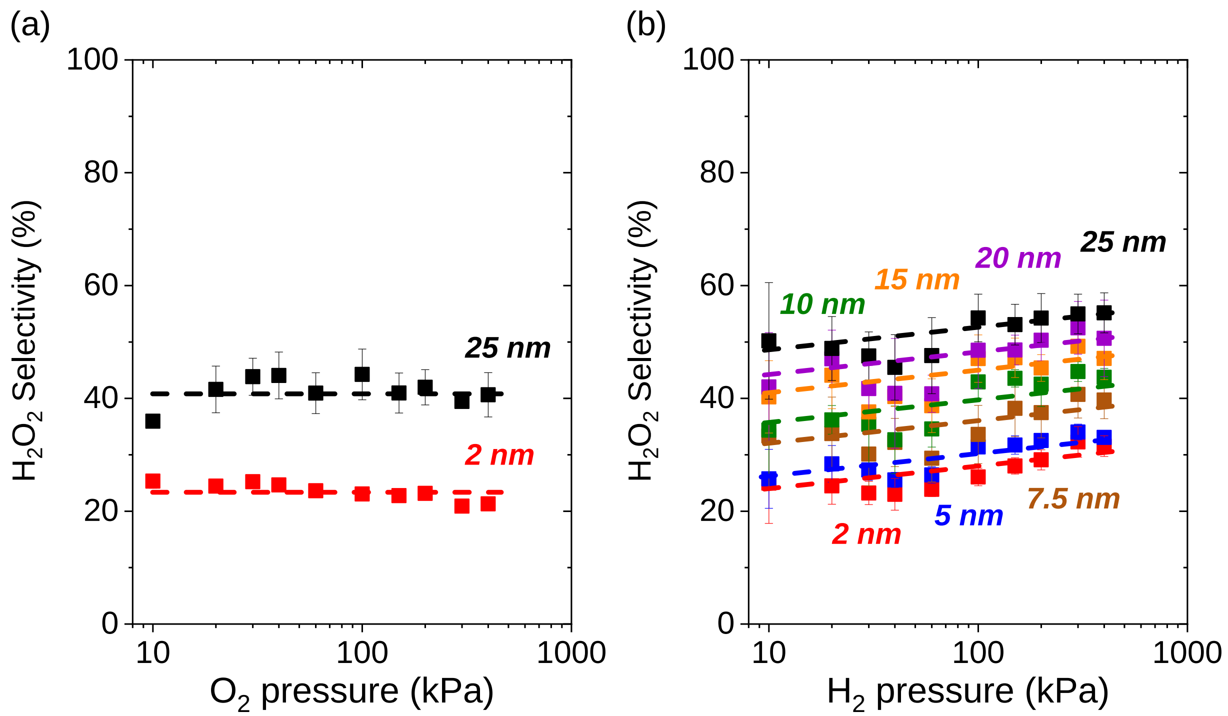

Figure S12. Steady-state selectivities for H_2O_2 formation on 2 nm (■), 5 nm (■), 7.5 nm (■), 10 nm (■), 15 nm (■), 20 nm (■), and 25 nm (■) Au nanoparticles supported on TiO_2 as functions of the pressure of (a) O_2 (10-400 kPa H_2, 60 kPa O_2), and (b) H_2 (10-400 kPa O_2, 60 kPa H_2) at 278 K. Dashed lines intended to guide the eyes.

Figure S12 shows that the selectivities of H_2O_2 formation do not depend on the pressure of O_2 but increase slightly as a function of H_2 pressure on Au-TiO_2 materials. Such findings suggest that H_2-rich conditions may lead to changes in the coverage of H_2-derived species that stabilize O–O bonds and favor the formation of H_2O_2. Figure S12 also shows that larger-size Au nanoparticles tend to show greater selectivities of H_2O_2 formation as nanoparticles increase in size. The result indicates an increase in the relative fraction of sites that stabilize O–O bonds on larger-sized nanoparticles, as discussed in greater detail in Sections 3.1 and 3.4.

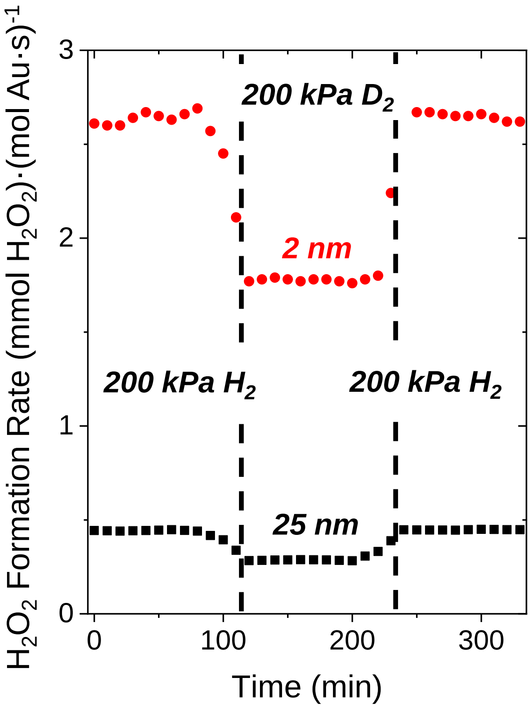

Figure. S13. H_2O_2 formation rates as a function of time in water on 2 nm (●) and 25 nm (■) Au nanoparticles supported on TiO_2 in a trickle-bed reactor (200 kPa H_2 or 200 kPa D_2 and 60 kPa O_2 at 278 K).

Figure S13 shows rates of H_2O_2 formation as a function of time within H_2-rich conditions (200 kPa H_2, 60 kPa O_2) and in response to changes in the identity of the hydrogen isotope. Here, gaseous H_2 is replaced with D_2 (200 kPa D_2, 60 kPa O_2) at ~ 110 minutes and subsequently returned to H_2 at ~ 230 minutes. Peroxide formation rates with H_2 as the reactant are a factor of 1.5 greater than those with D_2, which agrees with the conclusion that H-H (or D-D) bond dissociation proceeds in a kinetically relevant fashion.

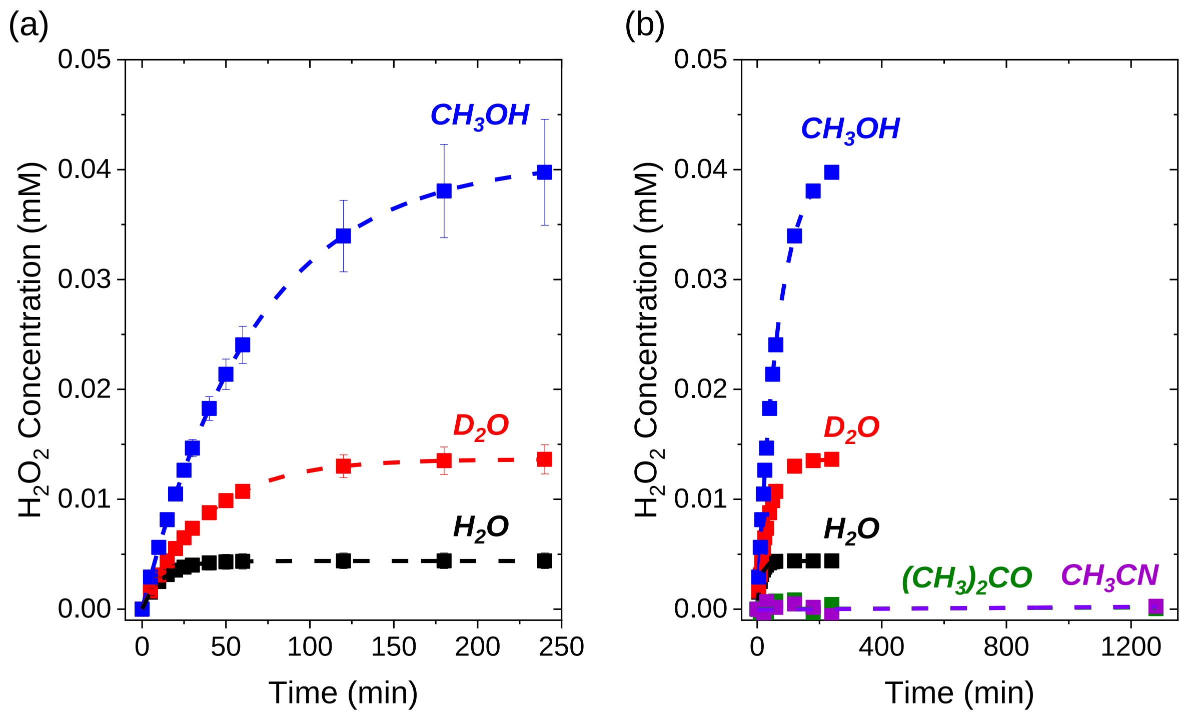

Figure S14. The cumulative concentration of H_2O_2 created over a period of (a) 4 hours and (b) 20 hours during direct synthesis when reacting H_2 with O_2 in methanol (■), water (■), heavy water (■), acetonitrile (■), or acetone (■) solvents (4.8 kPa H_2, 4.8 kPa O_2, 80 cm^3 solvent, 200 mg of 2 nm Au/TiO_2, 298 K). Dashed lines fitted to equations and analyzed using methods reported in our prior work.[2]

Figure S14 shows the transient concentration profiles of peroxide formation within protic (H_2O, CH_3OH), aprotic ($(CH_3)_2CO$, CH_3CN), and deuterated (D_2O) solvents. Concentration profiles for the formation of peroxides were fitted to equation S2.1 (derived in our prior work),[2] as shown by

$$[H_2O_2] = \frac{k_1}{k_2}(1 - e^{-k_2 t}) \tag{S2.1}$$

where the concentration of hydrogen peroxide ($[H_2O_2]$) depends on the primary rate constant of H_2O_2 formation (k_1) and secondary rate constants of H_2O_2 decomposition (k_2) as a function of time (t). Rates of H_2O_2 formation are immeasurable in aprotic solvents, which implicates proton transfer in the formation of H_2O_2. Comparisons of rate profiles within H_2O and D_2O solvents show similar formation rates of peroxide at early time intervals, but that peroxide accumulates to greater concentrations in D_2O, indicating rates of peroxide formation are similar within perhydrogenated and perdeuterated water, but peroxide decomposition rates differ between these solvents. Together, these findings suggest that proton transfer steps are not kinetically relevant for the primary formation of peroxide, consistent with kinetically relevant electron transfer steps. However, proton transfer steps seem kinetically relevant for the secondary decomposition peroxides during H_2O formation. Thus, water molecules may assist O–O dissociation steps of bound peroxide species.

Section S3: Full Mechanistic Derivation of Reactions of H_2 and O_2 at Au-Support Interface

In this section, we derive rate expressions describing the heterolytic reactions of H_2 and O_2 over supported Au nanoparticles, consistent with isotopic measurements and the functional dependence of H_2O_2 and H_2O formation rates with varying pressures of H_2 and O_2. Scheme S1 collectively refers to sites that bind hydrogen as # and sites that bind oxygen species as *.[3 4-9] Thus, this multi-site model distinguishes the site preference of reacting species and invokes proton-electron pairs observed previously by spectroscopic measurements of H_2 adsorption on supported Au materials,[10, 11] which simplifies the mathematics that derive H_2O_2 and H_2O turnover rates.[10, 12-16] The full details of these derivations follow.

$$
\begin{array}{rcll}
H_2 + \# + H_2O & \longrightarrow & H^{\#} + e^- + H_3O^+ & (1a) \\
H^{\#} + H_2O & \longrightarrow & \# + e^- + H_3O^+ & (1b) \\
O_2 + * & \rightleftharpoons & O_2* & (2) \\
O_2* + e^- + H^+ & \longrightarrow & OOH* & (3) \\
OOH* + e^- + H^+ & \longrightarrow & H_2O_2* & (4) \\
OOH* + e^- + H^+ & \longrightarrow & O* + H_2O & (5) \\
O* + e^- + H^+ & \longrightarrow & OH* & (6) \\
OH* + e^- + H^+ & \longrightarrow & H_2O* & (7) \\
H_2O_2* + e^- + H^+ & \longrightarrow & OH* + H_2O & (8) \\
H_2O_2* & \rightleftharpoons & H_2O_2 + * & (9) \\
H_2O* & \rightleftharpoons & H_2O + * & (10)
\end{array}
$$

Scheme S1. Proposed elementary steps for H_2O_2 and H_2O formation during reactions of H_2 and O_2 on supported Au nanoparticles, which couple electrochemical hydrogen oxidation and oxygen reduction reactions. Sites that bind hydrogen and oxygen-derived species are denoted as # and *, respectively. The symbols $\rightleftharpoons$ and $\longrightarrow$ indicate that elementary steps are quasi-equilibrated or irreversible, respectively.

The kinetically relevant dissociation of H_2 (step 1a) proceeds with the assistance of water molecules to generate chemisorbed $H^{\#}$ atoms, which then oxidize (step 1b) to form hydronium ions and electrons. These steps resemble the electrochemical Heyrovosky and Volmer reactions,[5, 17] present lower barriers than homolytic dissociation of H_2 (i.e., Tafel reaction),[18-20] and occur irreversibly (Figure 6). The resulting protons (H^+) may react with water in the solution to form H_3O^+ or with hydroxyl groups (M–OH) on the support to form $M–OH_2^+$, depending on the isoelectric point of the support.[21] Simultaneously, quasi-equilibrated adsorption of O_2 (step 2) forms O_2*,[3] which then reduces to form OOH* through proton and electron transfer (step 3).[2, 5, 10, 11, 22] Irreversible H_2 activation and oxidation (steps 1a and 1b) processes reflect rapid and irreversible PET steps that form OOH*, which agrees with prior DFT calculations indicating that PET occurs with low barriers and exothermically.[10, 11] Subsequently, the resulting OOH* reduces

to H_2O_2* (step 4) or dissociates into H_2O and O* (step 5), which can reduce further and form OH* (steps 6 and 8).[5, 23] The dissociation of dioxygen intermediates (steps 5 and 8) is highly exothermic, and isotopic labeling studies that implemented mixtures of $^{18}O_2$ and $^{16}O_2$ as reagents showed that O–O bonds do not reform once cleaved.[5, 24] Moreover, this treatment assumes that dioxygen species should directly form water (e.g., OOH* + e^- + H^+ → O* + H_2O) rather than dissociate across contiguous Au-atoms (e.g., OOH* + * → O* + OH*). Ultimately, the O* and OH* species reduce to water (H_2O*) by step 7. The bound H_2O_2* and H_2O* then undergo quasi-equilibrated desorption into the solution (steps 9 and 10) to yield H_2O and H_2O_2.

Scheme S1 and the dependence of rates on reactant pressures suggest that kinetically relevant hydrogen consumption ($-r_{H_2}$) limits the total rate of oxygen reduction ($-r_{O_2}$) on all supported Au nanoparticle catalysts examined. Thus, the reaction rate depends mainly on water-assisted activation of H_2 (step 1a) during the formation of H_2O_2 and H_2O, as shown by

$$-r_{H_2} = k_{1a}[H_2][H_2O][\#] = r_{H_2O} + r_{H_2O_2} \tag{S3.1}$$

where k_x is the rate constant of step x, and the total rate is a function of the number of unoccupied sites that bind H_2 ($[\#]$) and the activities of H_2 and H_2O ($[H_2]$ and $[H_2O]$, respectively). Here, the intrinsic rate constants k_x reflect the weighted reactivity of all active sites. Note that M–OH functions at the Au-support interface can also activate H–H bonds instead of H_2O, as suggested elsewhere.[29, 30] Scheme S1 also shows the stoichiometric rate of oxygen reduction as

$$-r_{O_2} = r_3 = \frac{1}{2}r_{H_2O} + r_{H_2O_2} \tag{S3.2}$$

where $-r_{O_2}$ depends on step 3 and determines the H_2O_2 and H_2O formation rates ($r_{H_2O_2}$ and r_{H_2O}).

Equations S3.1 and S3.2 can be further subdivided into

$$r_{H_2O_2} = r_4 = k_4[OOH^*][H^+][e^-] \tag{S3.3}$$

$$r_{H_2O} = 2r_5 = 2k_5[OOH^*][H^+][e^-] \tag{S3.4}$$

where the rates of H_2O_2 and H_2O formation depend on the activity of electrons ($[e^-]$) generated from hydrogen activation and the coverage of adsorbed peroxy intermediates ($[OOH^*]$). These species then react with protons ($[H^+]$) transferred either from hydronium ions (H_3O^+) or protonated hydroxyl species on the support (M–OH_2^+). These steps occur by either direct protonation (r_4, k_4) or dissociative reduction (r_5, k_5), yielding H_2O_2 and H_2O, respectively.

Thus, these expressions can be further analyzed by applying the pseudo-steady state hypothesis

$$\frac{d[OOH^*]}{dt} = 0 = (k_3[O_2^*] - (k_4 + k_5)[OOH^*])[H^+][e^-] \tag{S3.5}$$

and

$$[OOH^*] = \frac{k_3}{(k_4 + k_5)}[O_2^*] \tag{S3.6}$$

which depends on the relative rate that adsorbed dioxygen ($[O_2^*]$) reduces to $[OOH^*]$ by proton-electron transfer (k_3) versus the rate that $[OOH^*]$ reduces further to either H_2O_2 (k_4) or H_2O (k_5).

Combining equations S3.3, S3.4, and S3.6 yields the product formation rates

$$r_{H_2O_2} = \left(\frac{k_4 k_3}{k_4 + k_5}\right)[O_2^*][H^+][e^-] = \left(\frac{k_4}{k_4 + k_5}\right) r_3 \tag{S3.7}$$

and

$$r_{H_2O} = \left(\frac{2k_5 k_3}{k_4 + k_5}\right)[O_2^*][H^+][e^-] = \left(\frac{2k_5}{k_4 + k_5}\right) r_3 \tag{S3.8}$$

where equations S3.7 and S3.8 suggest that the average rate of oxygen reduction reflects the ratio of the rates of OOH* hydrogenation (k_4) to dissociation (k_5), which determines the selectivity of H_2O_2 formation. Steps 8 and 9 also influence H_2O_2 selectivity since H_2O_2 adsorption and desorption events compete with O–O dissociation reactions that form H_2O. Such steps become significant when H_2O_2 concentrations rise with increasing reactant conversion. However, these rate measurements were conducted at low conversions of H_2 (< 5%) and high volumetric flow rates to suppress contributions from the secondary decomposition of H_2O_2. Consequently, the steady-state rate measurements obtained provide data useful for inspecting derived rates expressions for primary product formation pathways (i.e., steps 4 and 5).

Combining equations S3.1, S3.7, S3.8 yields

$$k_{1a}[H_2][H_2O][\#] = \left(\frac{k_4 + 2k_5}{k_4 + k_5}\right) r_3 \tag{S3.9}$$

and

$$r_3 = \left(\frac{k_4 + k_5}{k_4 + 2k_5}\right) k_{1a}[H_2][H_2O][\#] \tag{S3.10}$$

where the rate of oxygen reduction directly relates to the rate of hydrogen oxidation weighted by the average numbers of electrons transmitted ($(2k_4 + 4k_5)/\,(k_4 + k_5)$), in which 4 electrons transfer during the formation of H_2O and 2 electrons transfer during the formation of H_2O_2.

Subsequently, substituting equation S3.10 into equations S3.7 and S3.8 yields

$$r_{H_2O_2} = \left(\frac{k_4}{k_4 + 2k_5}\right) k_{1a}[H_2][H_2O][\#] \tag{S3.11}$$

and

$$r_{H_2O} = \left(\frac{2k_5}{k_4 + 2k_5}\right) k_{1a}[H_2][H_2O][\#] \tag{S3.12}$$

where the rates of H_2O_2 and H_2O formation depend directly on the activity of H_2. Here, the total H_2O_2 selectivity reflects the average H_2O_2 selectivity ($k_4/(k_4 + 2k_5)$) of all catalytic active sites that perform oxygen reduction.

The coverage of sites that bind H_2 can be further analyzed by the pseudo-steady state hypothesis

$$\frac{d[H^{\#}]}{dt} = 0 = k_{1a}[H_2][H_2O][\#] - k_{1b}[H^{\#}][H_2O] \tag{S3.13}$$

and

$$[H^{\#}] = \frac{k_{1a}}{k_{1b}} [H_2][\#] \tag{S3.14}$$

where the coverage of hydrogen ($[H^{\#}]$) depends on the rate that it heterolytically adsorbs to the surface of the catalyst (k_{1a}) versus the rate that it is consumed by subsequent reactions (k_{1b}).

Consequently, the total site balance reflects

$$[L_{\#}] = [\#] + [H^{\#}] \tag{S3.15}$$

and

$$[L_{\#}] = [\#]\left(1 + \frac{k_{1a}}{k_{1b}} [H_2]\right) \tag{S3.16}$$

where the total number of sites that bind hydrogen ($[L_{\#}]$) depend on the sum of unoccupied sites ($[\#]$) and sites occupied by hydrogen atoms ($[H^{\#}]$). Thus, combining equations S3.11 and S3.12 with equation S3.16 yields the complete rate expressions

$$\frac{r_{H_2O_2}}{[L_{\#}]} = \left(\frac{k_4}{k_4 + 2k_5}\right)\left(\frac{k_{1a}[H_2][H_2O]}{1 + \frac{k_{1a}}{k_{1b}}[H_2]}\right) \tag{S3.17}$$

and

$$\frac{r_{H_2O}}{[L_{\#}]} = \left(\frac{2k_5}{k_4 + 2k_5}\right)\left(\frac{k_{1a}[H_2][H_2O]}{1 + \frac{k_{1a}}{k_{1b}}[H_2]}\right) \tag{S3.18}$$

where the H_2O_2 and H_2O turnover rates depend on the rate of hydrogen consumption multiplied by the selectivity to either product.

Equations S3.17 and S3.18 predict that H_2O_2 and H_2O turnover rates should increase in proportion with $[H_2]$ and approach constant values as $[H^{\#}]$ saturates the surface at the greatest pressures. This conclusion agrees with Figures 4 and 5 where rates increase linearly with H_2 pressures, which suggests $H^{\#}$ species exist at low fractional coverages across this range of conditions (10-400 kPa H_2, 60 kPa O_2, 278 K). The low coverages of $H^{\#}$ on Au surfaces agree with expectations based on the kinetic relevance of H_2 dissociation, the rapid consumption of $H^{\#}$ by heterolytic oxidation, and the weak binding of H-atoms to supported Au catalysts.[18-20] Moreover, such expressions suggest rates do not depend on the pressure of O_2, which is consistent with Figures 4 and 5 (10-400 kPa O_2, 60 kPa H_2, 278 K). Thus, oxygen-derived species represent the most abundant reactive intermediates (MARI) on active sites across these conditions. Therefore, equations S3.17 and S3.18 take the form

$$\frac{r_{H_2O_2}}{[L_{\#}]} = \frac{k_{1a}k_4}{k_4 + 2k_5}[H_2][H_2O] \tag{S3.19}$$

and

$$\frac{r_{H_2O}}{[L_{\#}]} = \frac{2k_{1a}k_5}{k_4 + 2k_5}[H_2][H_2O] \tag{S3.20}$$

where turnover rates depend directly on the pressure of H_2 at the conditions used in this study. Indeed, equations S3.19 and S3.20 also explain the similar H_2/D_2 kinetic isotope effects (Table 3) for H_2O_2 and H_2O formation since the isotopic label should only affect the value of k_{1a} independent of the ORR reactivity on the metallic or interfacial sites (i.e., kinetic isotope effects do not change with Au nanoparticle diameter).

Together, these findings suggest that solid-liquid-support interfaces catalyze proton-electron transfer reactions of H_2, O_2, and H_2O, limited by H–H activation steps, as shown by Scheme S2. Moreover, the kinetic behavior and reaction mechanisms proposed here for reducing O_2 with H_2 over supported Au nanoparticles in water resemble aspects of prior studies on Au catalysis, particularly publications by Chandler et al. We observe similar importance of oxygen functions at the Au-support interface during H–H activation, proportional dependencies on H_2 pressure (3-20 kPa H_2, 10 kPa O_2, 333 K), and a strong influence of H_2O on catalysis.[10, 12] The presence of liquid water leads to significant formation rates of H_2O_2, which suggest H_2O molecules stabilize the O–O bonds of these reactive species more effectively at solid-liquid interfaces than under dry or humid conditions. Furthermore, our findings show that the support presents interfacial moieties that impact the stability of these O–O bonds and change the relative proportion of H_2O_2 and H_2O formation.

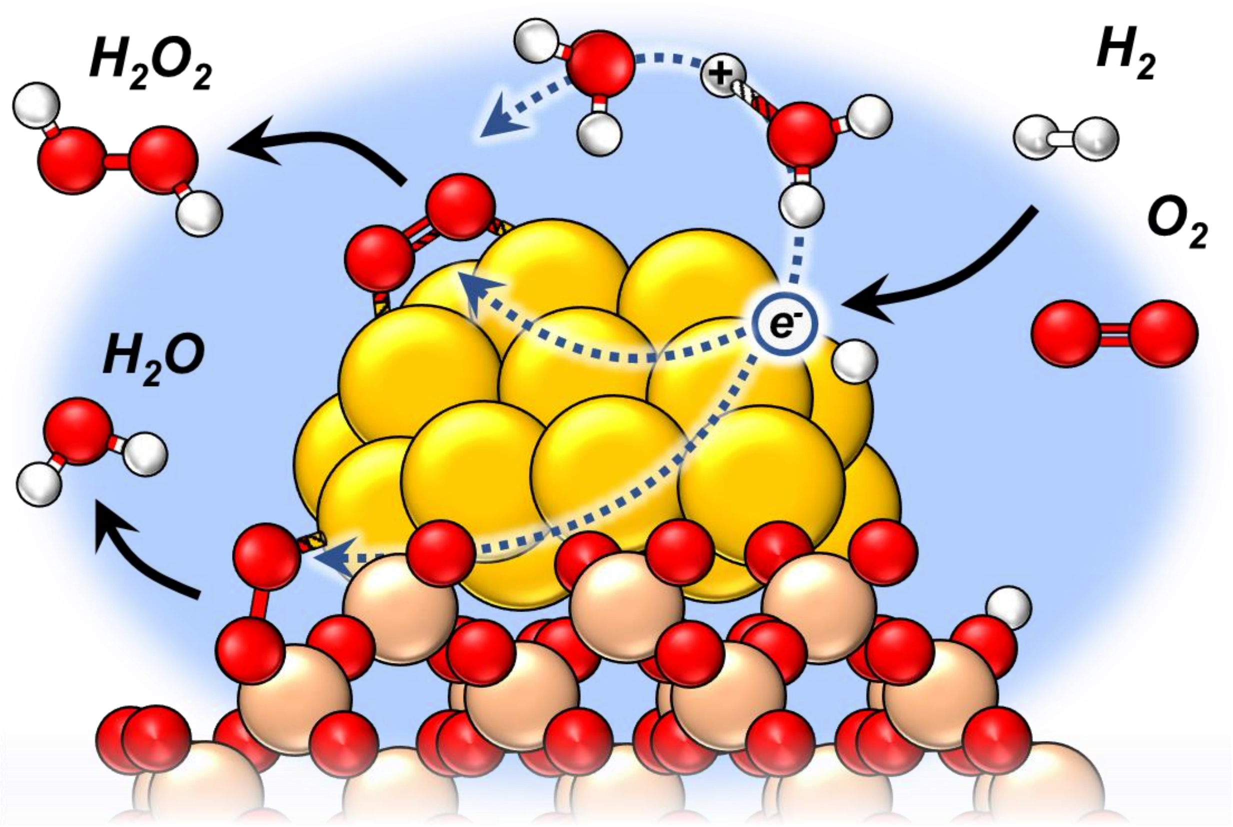

Scheme S2. Scheme of the proposed mechanism involving kinetically relevant activation of H_2 coupled to O_2 reduction on metallic and interfacial Au atoms on a catalytic Au nanoparticle.

Section S4: Analysis of Infrared Spectra of CO Adsorbed on Titania-Supported Au Nanoparticles

In this section, we interpret the spectra of CO adsorbed onto catalytic materials to elucidate the role of different surface sites on reaction kinetics. Figure S15a-e shows steady state spectra of CO adsorbed on TiO_2 and Au nanoparticles supported on TiO_2 at many pressures of CO (0.005 -1 kPa CO, 303 K). These spectra show features agreeing with CO adsorbed linearly atop Au nanoparticles (2119 cm^{-1}) and TiO_2 (2194 cm^{-1}),[25] carbonate species (bidentate [1470-1530 cm^{-1}]; mondentate [1300-1400 cm^{-1}]),[25-27] and water (2500-3600 cm^{-1}; 1430-1630cm^{-1}).[25, 28] At the highest pressures of CO, there are significant contributions of gas phase CO (2000-2250 cm^{-1}) that convolute the spectra of adsorbed species. Generally, the intensity of linear CO attenuated on all samples as the pressure of CO decreases, consistent with a decrease in the coverage of CO on Au nanoparticles and TiO_2. The intensity of these features were fit to the Langmuir equation

$$\theta_{CO} = \frac{P}{P_{max}} = \frac{K_{CO}[CO]}{1 + K_{CO}[CO]} \tag{S4.1}$$

Where the coverage of CO (θ_{CO}) reflects the ratio of peak intensity (P) to the maximum peak intensity (P_{max}), which depends on the equilibrium constant of CO adsorption (K_{CO}) and the pressure of CO ($[CO]$). The resulting isotherms for each material are shown in Figure S15f (deconvoluted from gas-phase CO features) and fits for K_{CO} are reported in Table S2. These data show that TiO_2 binds CO weaker than any of the supported Au nanoparticles. Moreover, each Au catalyst binds CO with similar strength with the exception of the 25 nm Au particles. This discrepancy may result from some intrinsic change in the structure of the largest Au nanoparticles or could reflect the poor signal-to-noise of this specific measurement. Regardless, these findings suggest that the linearly adsorbed CO does not bind with significantly different energies as Au nanoparticles increase in size across this range of diameters (5 – 25 nm) on TiO_2.

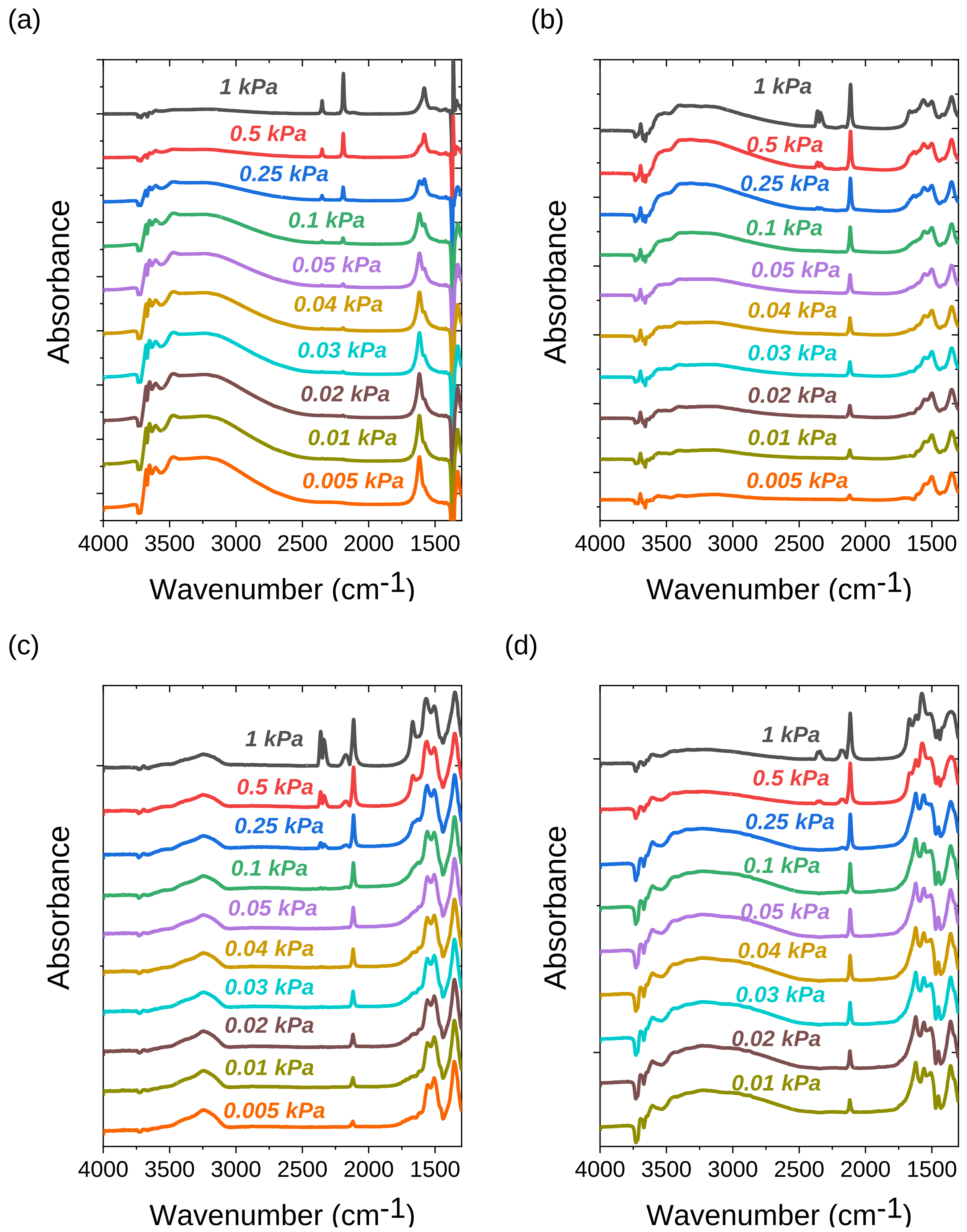

(a)
1 kPa
0.5 kPa
0.25 kPa
0.1 kPa
0.05 kPa
0.04 kPa
0.03 kPa
0.02 kPa
0.01 kPa
0.005 kPa
Absorbance
4000 3500 3000 2500 2000 1500
Wavenumber (cm-1)
(b)
1 kPa
0.5 kPa
0.25 kPa
0.1 kPa
0.05 kPa
0.04 kPa
0.03 kPa
0.02 kPa
0.01 kPa
0.005 kPa
Absorbance
4000 3500 3000 2500 2000 1500
Wavenumber (cm-1)
(c)
1 kPa
0.5 kPa
0.25 kPa
0.1 kPa
0.05 kPa
0.04 kPa
0.03 kPa
0.02 kPa
0.01 kPa
0.005 kPa
Absorbance
4000 3500 3000 2500 2000 1500
Wavenumber (cm-1)
(d)
1 kPa
0.5 kPa
0.25 kPa
0.1 kPa
0.05 kPa
0.04 kPa
0.03 kPa
0.02 kPa
0.01 kPa
Absorbance
4000 3500 3000 2500 2000 1500
Wavenumber (cm-1)

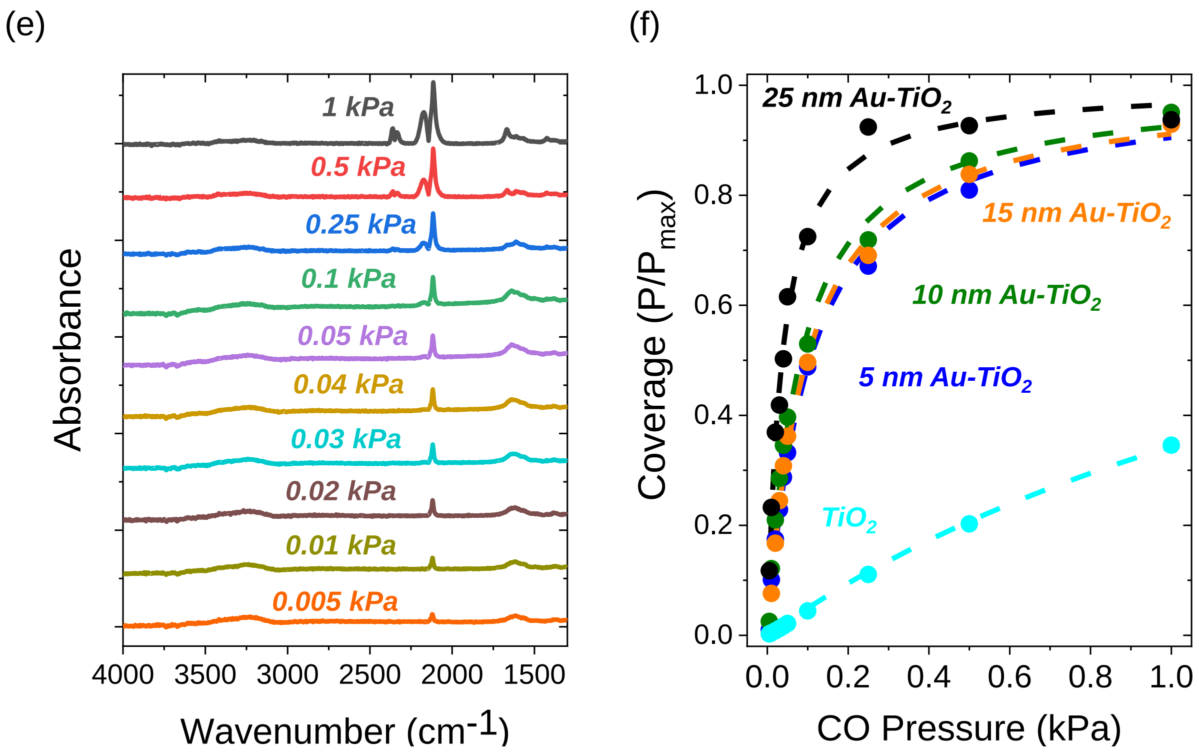

Figure S15. Ex situ infrared spectra were collected at varying pressures of CO (0.005-1 kPa CO, 100-101 kPa He, 303 K) upon (a) TiO_2 and (b) 5 nm, (c) 10 nm, (d) 15 nm, and (e) 25 nm Au nanoparticles supported on TiO_2. (b) Langmuir isotherms showing fractional coverage of CO on TiO_2 and Au nanoparticles supported on TiO_2 are fitted to equation S4.1. Measurements were conducted upon catalytic materials (3 wt% Au) following an oxidative treatment (20 kPa O_2, 573 K).

Table S2. Table of fit equilibrium constants for CO adsorption on TiO_2 and Au nanoparticles supported on TiO_2 using date reported in Figure S15f.

Material	K_{CO} (kPa^{-1})
TiO_2	0.5
5 nm Au-TiO_2	9.5
10 nm Au-TiO_2	12.4
15 nm Au-TiO_2	10.3
25 nm Au-TiO_2	27.8

Figures S15a-e also show that samples achieve a steady state coverage of carbonates at 1 kPa of CO, but these features remain persistent even when approaching low pressures of CO (e.g., 0.01 kPa). These observations suggest these species are bound to the material irreversibly under these conditions. Such conclusions agree with prior studies, demonstrating that carbonate species poison CO oxidation catalysis by blocking interfacial sites at the perimeter of Au nanoparticles.[29, 30] Spectra of adsorbed CO on TiO_2 and Au-TiO_2 show features consistent with η^1-$CO_3{*}_{Ti}$ [ν_s(C–O) = 1300-1420 cm^{-1}] and η^2-$CO_3{*}_{Ti}$ [ν_a(C–O) = 1550-1610 cm^{-1}] on each material. Still, these

features are far more monodisperse on pure TiO_2. By comparison, Au-TiO_2 shows many similar features and the emergence of distinct CO-derived features that form only in the presence of the Au-support interface. Thus, we hypothesize that the relative peak area of linear CO (A_{CO-Au}) and carbonates (A_{CO_3}) should serve as a probe for the ratio of metallic Au atoms relative to sites at the Au-support interface of TiO_2. Consequently, we compare the integrated peak area of linear CO to that for carbonates at low CO pressures (0.01 kPa CO) to avoid contributions from gas-phase CO. Next, we aim to relate these FTIR measurements of adsorbed CO to empirical predictions of site ratios. Specifically, we estimated this fraction using geometric correlations empirically reported by Ribeiro[31] and equivalently derived by Hereveld and Heretog[32] for a cuboctahedral geometry.

$$\theta_t = 0.9 \cdot \left(< d_{TEM,S} >\right)^{-0.7} \tag{S4.2}$$

$$\theta_p = 0.46 \cdot \left(< d_{TEM,S} >\right)^{-1.8} \tag{S4.3}$$

$$\frac{\theta_t}{\theta_p} = 1.96 \cdot \left(< d_{TEM,S} >\right)^{1.1} \tag{S4.4}$$

Where the fraction of total Au atoms located at the surface of Au nanoparticles (θ_t) and the fraction of Au atoms that reside at the perimeter of nanoparticles (θ_p) (i.e., at the Au-support interface) depend on the mean diameter of the Au nanoparticles $< d_{TEM,S} >$. We expect that this site ratio should directly relate to the ratio of integrated peak areas associated with CO bound linearly to metallic Au surfaces (A_{CO-Au}) versus that of carbonate features formed from the reaction of CO and TiO_2 at the Au-support interface (A_{CO_3}). We quantified the contributions from the FTIR spectra as

$$A_{CO-Au} = \int_{2000\ cm^{-1}}^{2250\ cm^{-1}} P(\nu)\, d\nu \tag{S4.5}$$

$$A_{CO_3} = \int_{1300\ cm^{-1}}^{1800\ cm^{-1}} P(\nu)\, d\nu \tag{S4.6}$$

Where A_{CO-Au} and A_{CO_3} are integrated from the spectral data (P) with respect to wavenumbers (ν) between 2000-2250 cm^{-1} and 1300-1800 cm^{-1}, respectively.

While we suspect that these site ratios are directly related (i.e., $A_{CO-Au}/A_{CO_3} \propto \theta_t/\theta_p$), they have different bases. Thus, it is necessary to normalize these parameters by an internally consistent site ratio (e.g., $\left(\theta_t/\theta_p\right)_{x\ nm}$). Thus, it was convenient to normalize these relative site fractions for a standard nanoparticle size of 5 nm, giving

$$\frac{\left(\theta_t/\theta_p\right)_{<d_{TEM,S}>}}{\left(\theta_t/\theta_p\right)_{5\ nm}} = \frac{\left(< d_{TEM,S} >\right)^{1.1}}{5.87} \tag{S4.7}$$

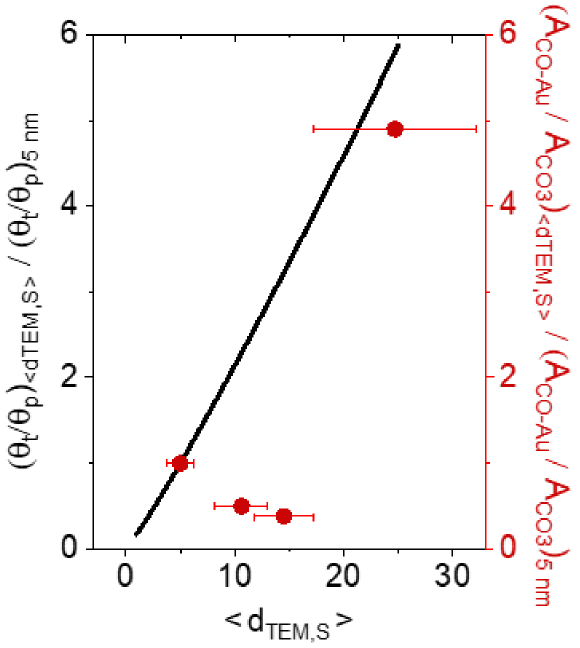

Figure S16. Comparisons of $(\theta_t/\theta_p)_{<d_{TEM,S}>}/(\theta_t/\theta_p)_{5\ nm})$ from equation S4.5 and equivalent FTIR-derived ratios of $(A_{CO-Au}/A_{CO_3})_{<d_{TEM,S}>}/(A_{CO-Au}/A_{CO_3})_{5\ nm}$, ploted as a function of the diameter of Au nanoparticles supported on TiO_2.

Figure S16 shows direct comparisons of $(\theta_t/\theta_p)_{<d_{TEM,S}>}/(\theta_t/\theta_p)_{5\ nm})$ and equation S4.7 as a probe for the relative quantity of perimeter sites versus total surface Au atoms, normalized relative to 5 nm Au nanoparticles. Generally, the empirically calculated values agree with empirical correlations, showing the best agreement with 25 nm nanoparticles. Unexpectedly, the values for the 10 and 15 nm Au nanoparticles deviate from the expected trend, possibly due to the convolution of CO_3 features formed on the support far from the Au-TiO_2 interface. Still, this analysis provides evidence that FTIR spectra of adsorbed CO can be used as a descriptor for estimating fractions of sites present on Au nanoparticles versus the perimeter of the Au-support interface. Regardless, plotting the rate ratios of H_2O_2 and H_2O formation versus equation S4.4 (Figure 8b) shows a strong correlation (R^2 = 0.991), suggesting that sites at the Au-support interface favor the formation of H_2O. In contrast, the remaining surface Au atoms favor the formation of H_2O_2.

Section S5: Interpretation of Apparent Activation Barriers and Site Requirements

In this section, we mathematically interpret the temperature dependence of rate expressions derived in Section S3. All elementary reactions can be broken down using the Eyring equation

$$k_x = \frac{k_b T}{h} e^{-\frac{\Delta G_x^\ddagger}{RT}} = \frac{k_b T}{h} e^{\frac{\Delta S_x^\ddagger}{R}} e^{-\frac{\Delta H_x^\ddagger}{RT}} \tag{S5.1}$$

where elementary rate constant (k_x) depends on the Boltzmann constant (k_b), Planck's constant (h), universal gas constant (R), temperature (T), and the activation Gibbs free energy ($\Delta G_x^\ddagger$) of initiating the transition state of step x. This expression further subdivides into the activation enthalpy ($\Delta H_x^\ddagger$) and entropy ($\Delta S_x^\ddagger$) of the same elementary reaction.

Combing equation S5.1 with equations S3.19 and S3.20 yields

$$k_{H_2O_2} = \frac{k_{1a} k_4}{k_4 + 2k_5} = \frac{k_b T}{h} \frac{e^{-\frac{\left(\Delta G_{1a}^\ddagger + \Delta G_4^\ddagger\right)}{RT}}}{e^{-\frac{\Delta G_4^\ddagger}{RT}} + 2e^{-\frac{\Delta G_5^\ddagger}{RT}}} \tag{S5.2}$$

and

$$k_{H_2O} = \frac{2k_{1a} k_5}{k_4 + 2k_5} = \frac{k_b T}{h} \frac{2e^{-\frac{\left(\Delta G_{1a}^\ddagger + \Delta G_5^\ddagger\right)}{RT}}}{e^{-\frac{\Delta G_4^\ddagger}{RT}} + 2e^{-\frac{\Delta G_5^\ddagger}{RT}}} \tag{S5.3}$$

where the apparent rate constants of forming H_2O_2 ($k_{H_2O_2}$) and H_2O (k_{H_2O}) include terms related to the activation of H_2 and the reduction of OOH* species, described by the activation Gibbs free energies of steps 1a ($\Delta G_{1a}^\ddagger$), 4 ($\Delta G_4^\ddagger$), and 5 ($\Delta G_5^\ddagger$).

Summing equations S5.2 and S5.3 yields

$$k_{H_2} = k_{1a} = \frac{k_b T}{h} e^{-\frac{\Delta G_{1a}^\ddagger}{RT}} = e^{\frac{\Delta S_{1a}^\ddagger}{R}} e^{-\frac{\Delta H_{1a}^\ddagger}{RT}} \tag{S5.4}$$

where the apparent rate constant of H_2 activation (k_{H_2}) depends on step 1 (k_{1a}) and the activation Gibb's free energy ($\Delta G_{1a}^\ddagger$), entropy ($\Delta S_{1a}^\ddagger$), and enthalpy ($\Delta H_{1a}^\ddagger$) of H_2 consumption.

More explicit derivations of the apparent barriers are derived from the Van't Hoff equation

$$\Delta H_{app}^\ddagger = RT^2 \frac{dln\left(\frac{h}{k_b T} k_{app}\right)}{dT} \tag{S5.5}$$

where the experimentally measured barriers of product formation should directly relate to the derivative of the apparent rate constant with respect to temperature.

Combining equations S5.4 with equation S5.5 yields

$$\Delta H^{\ddagger}_{H_2} = \Delta H^{\ddagger}_{1a} \tag{S5.6}$$

where the apparent barrier of H_2 activation ($\Delta H^{\ddagger}_{H_2}$) is equal to $\Delta H^{\ddagger}_{1a}$, which directly relates to the energetics of activating an H–H bond.

As such, combining equations S5.2 and S5.3 with equation S5.6 yields

$$\Delta H^{\ddagger}_{H_2O_2} = \Delta H^{\ddagger}_{1a} - \frac{2\left(\Delta H^{\ddagger}_5 - \Delta H^{\ddagger}_4\right)e^{-\frac{\Delta G^{\ddagger}_5}{RT}}}{e^{-\frac{\Delta G^{\ddagger}_4}{RT}} + 2e^{-\frac{\Delta G^{\ddagger}_5}{RT}}} \tag{S5.7}$$

and

$$\Delta H^{\ddagger}_{H_2O} = \Delta H^{\ddagger}_{1a} + \frac{\left(\Delta H^{\ddagger}_5 - \Delta H^{\ddagger}_4\right)e^{-\frac{\Delta G^{\ddagger}_4}{RT}}}{e^{-\frac{\Delta G^{\ddagger}_4}{RT}} + 2e^{-\frac{\Delta G^{\ddagger}_5}{RT}}} \tag{S5.8}$$

where the apparent barriers of forming H_2O_2 ($\Delta H^{\ddagger}_{H_2O_2}$) and H_2O ($\Delta H^{\ddagger}_{H_2O}$) depend directly on $\Delta H^{\ddagger}_{1a}$ and the difference in apparent barriers of H_2O_2 and H_2O formation. However, this difference in barriers is weighted by the relative contribution of the H_2O_2 and H_2O formation pathways, determined by $\Delta G^{\ddagger}_4$ and $\Delta G^{\ddagger}_5$.

While equations S5.7 and S5.8 explicitly describe the measured barriers, it is challenging to relate these expressions to experimental data directly. Taking the difference between the apparent activation enthalpies yields a more interpretable set of quantities

$$\Delta\Delta H^{\ddagger} = \Delta H^{\ddagger}_{H_2O} - \Delta H^{\ddagger}_{H_2O_2} = \Delta H^{\ddagger}_5 - \Delta H^{\ddagger}_4 \tag{S5.9}$$

where the difference in apparent barriers of H_2O_2 and H_2O formation ($\Delta\Delta H^{\ddagger}$) can compare directly to experimental measurements and reveal the differences between intrinsic activation enthalpies for steps 4 and 5.

An equivalent simplification is derived from the ratio of equations S5.2 and S5.3

$$\frac{r_{H_2O}}{r_{H_2O_2}} = \frac{k_5}{k_4} = \frac{e^{-\frac{\Delta G^{\ddagger}_5}{RT}}}{e^{-\frac{\Delta G^{\ddagger}_4}{RT}}} = e^{\frac{\Delta S^{\ddagger}_5 - \Delta S^{\ddagger}_4}{R}} \cdot e^{-\frac{\Delta H^{\ddagger}_5 - \Delta H^{\ddagger}_4}{RT}} \tag{S5.10}$$

where the differences between Gibbs free energies of steps 4 ($\Delta G^{\ddagger}_4$) and 5 ($\Delta G^{\ddagger}_5$) reflect the difference in apparent barriers of H_2O_2 and H_2O formation (i.e., $\Delta\Delta H^{\ddagger} = \Delta H^{\ddagger}_{H_2O} - \Delta H^{\ddagger}_{H_2O_2} = \Delta H^{\ddagger}_5 - \Delta H^{\ddagger}_4$) and their entropic contributions ($\Delta S^{\ddagger}_5, \Delta S^{\ddagger}_4$). Collectively, these expressions are used to interpret the energetic data presented throughout the rest of this section.

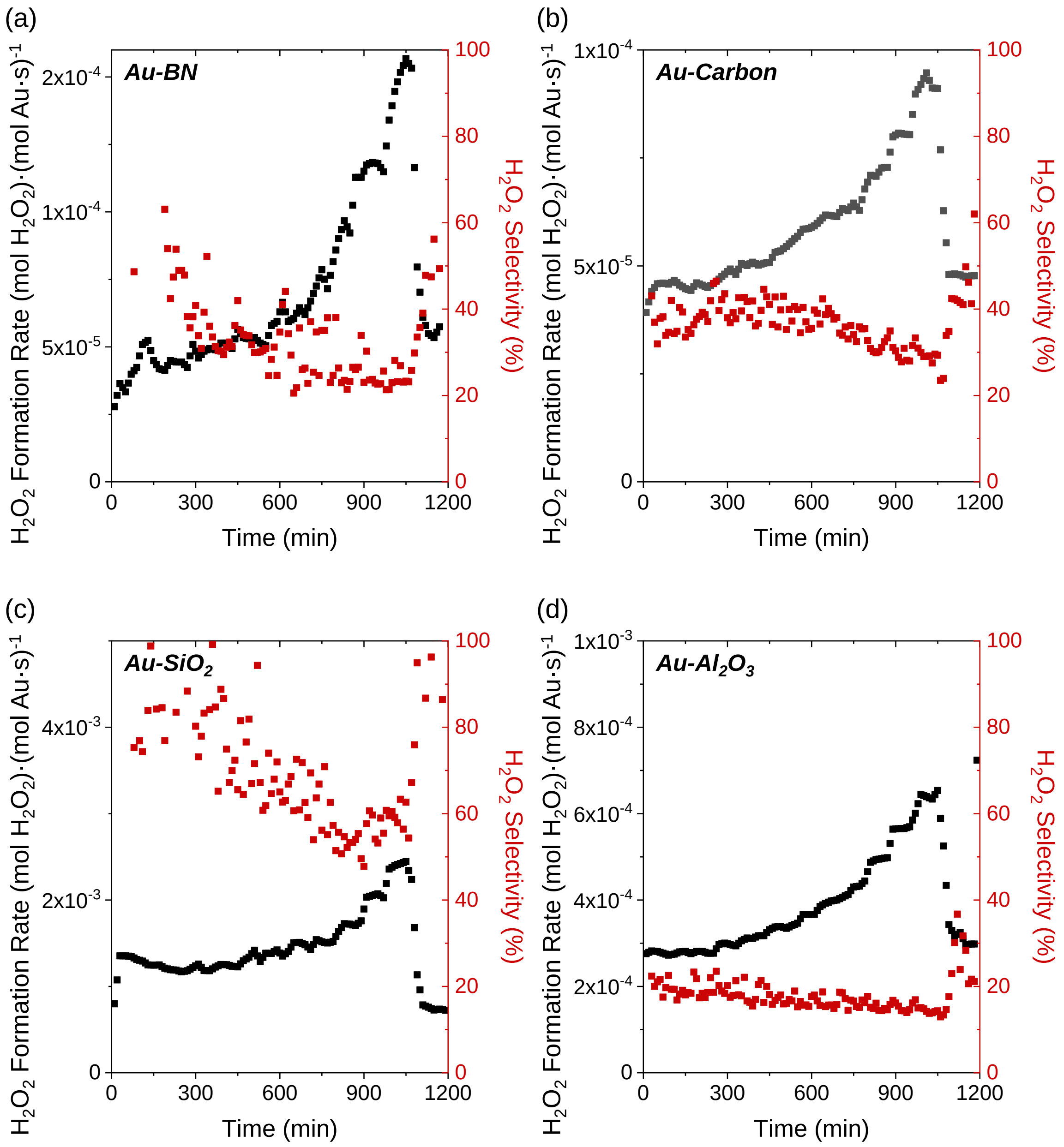

(a)
Au-BN
H_2O_2 Formation Rate (mol H_2O_2)·(mol Au·s)$^{-1}$
H_2O_2 Selectivity (%)
Time (min)
(b)
Au-Carbon
H_2O_2 Formation Rate (mol H_2O_2)·(mol Au·s)$^{-1}$
H_2O_2 Selectivity (%)
Time (min)
(c)
Au-SiO$_2$
H_2O_2 Formation Rate (mol H_2O_2)·(mol Au·s)$^{-1}$
H_2O_2 Selectivity (%)
Time (min)
(d)
Au-Al$_2$O$_3$
H_2O_2 Formation Rate (mol H_2O_2)·(mol Au·s)$^{-1}$
H_2O_2 Selectivity (%)
Time (min)

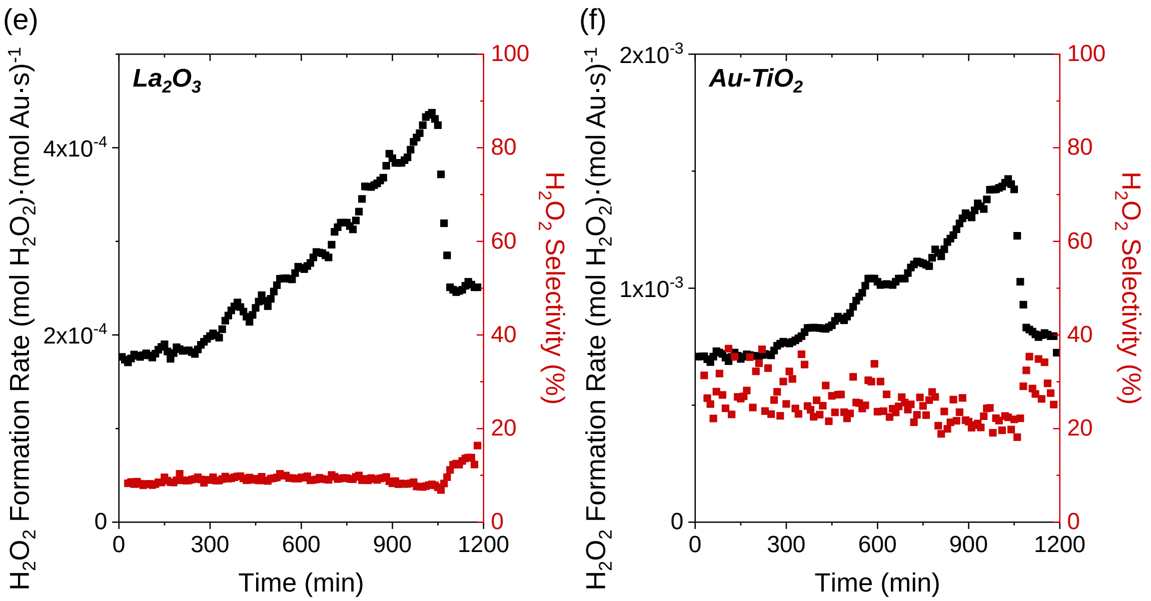

Figure S17. Representative time-on-stream measurements of transient rates and selectivities of H_2O_2 formation on 2-3 nm Au nanoparticles supported on (a) BN, (b) Carbon, (c) SiO_2, (d) Al_2O_3, (e) La_2O_3, and (e) TiO_2 at standard conditions (200 kPa H_2, 60 kPa O_2) over varying temperatures (278-308 K). Data from these plots were corrected for deactivation and other measurements, conducted in triplicate, were used to formulate plots in Figure S23.

Figure S17 shows the transient measurements of rate and selectivities of H_2O_2 formation on Au nanoparticles supported on BN, Carbon, SiO_2, Al_2O_3, La_2O_3, and TiO_2. Measurements began at 278 K for 270 minutes, after which the temperature was increased in intervals of 90 minutes until achieving 308K before finally returning to the initial temperature of 278 K after 1080 minutes on stream and held for an additional 180 minutes. Generally, rates and selectivities changed little on most materials, and changes were typically reversible between subsequent measurements. Deactivation on Au-SiO_2 materials, however, was significant and required the most correction for deactivation. In all cases, deactivation was accounted for by assuming that the number of active sites decreased by some exponential function between the beginning (~180-270 min) and the end of the measurement period (~1100-1190 min). Rates were corrected such that the average rate of H_2O_2 formation in the initial period at 278K was equal to the average rate following the return to this temperature at the end of the measurement.

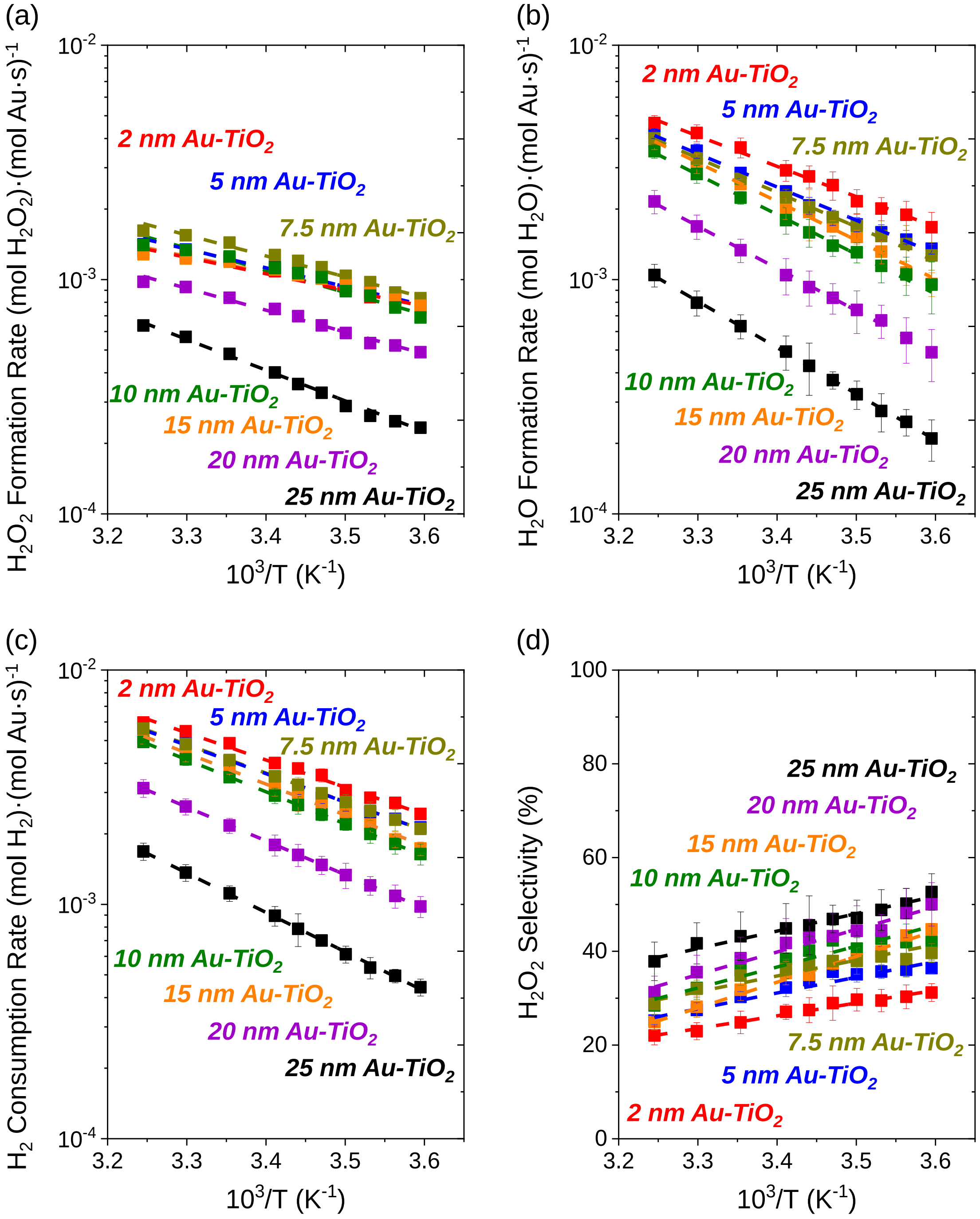

Figure S18. Steady-state rates of (a) H_2 consumption, (b) H_2O_2 formation, (c) H_2O formation, and (d) H_2O_2 selectivity as a function of inverse temperature (278-308 K) on 2 nm (■), 5 nm (■), 7.5 nm (■), 10 nm (■), 15 nm (■), 20 nm (■), and 25 nm (■) Au nanoparticles supported on TiO_2 at standard conditions (200 kPa H_2, 60 kPa O_2). The dashed lines represent fits to equation S5.1 and give apparent activation enthalpies shown in Figure 9.

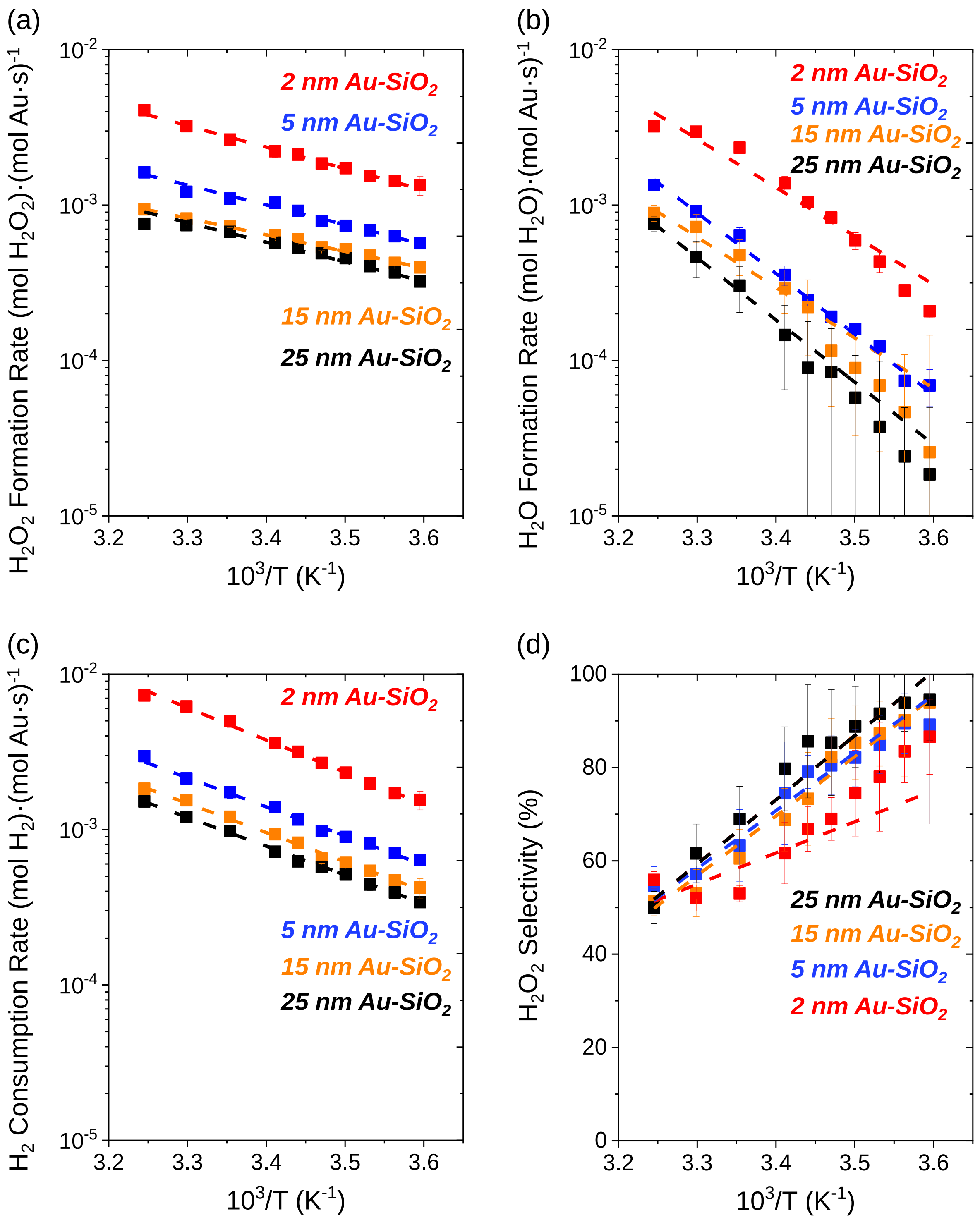

Figure S19. Steady-state rates of (a) H_2 consumption, (b) H_2O_2 formation, (c) H_2O formation, and (d) H_2O_2 selectivity as a function of inverse temperature (278-308 K) on 2 nm (■), 5 nm (■), 15 nm (■), and 25 nm (■) Au nanoparticles supported on SiO_2 at standard conditions (200 kPa H_2, 60 kPa O_2). The dashed lines represent fits to equation S5.1 and give apparent activation enthalpies shown in Figure 9.

Figures S18 and S19 show the temperature dependence of rates for H_2 consumption, H_2O_2 formation, H_2O formation, and for H_2O_2 selectivity (278-308 K) over Au nanoparticles of different mean diameters (2 – 25 nm) supported on TiO_2 and SiO_2, respectively. Fits of these profiles using the Eyring equation (equation S5.1) yield values for the apparent activation enthalpies of H_2 consumption, H_2O_2 formation, and H_2O formation. Note that the fits for the 2 nm Au-SiO_2 were evaluated in the linear region of this Arrhenius plot and were evaluated between 278-298K for this data set, equivalently shown in Figure S23. Further analysis of the difference in apparent activation barriers of H_2 consumption and H_2O_2 and H_2O formation lead to the definitions of $\Delta H^{\ddagger}_{H_2}$ and $\Delta\Delta H^{\ddagger}$shown by equations S5.6 and S5.9, respectively. These data agree with trends in H_2 consumption and H_2O_2 selectivity shown in Figure 2. Further interpretation of these data is reported in Section 3.4 and Figure 9.

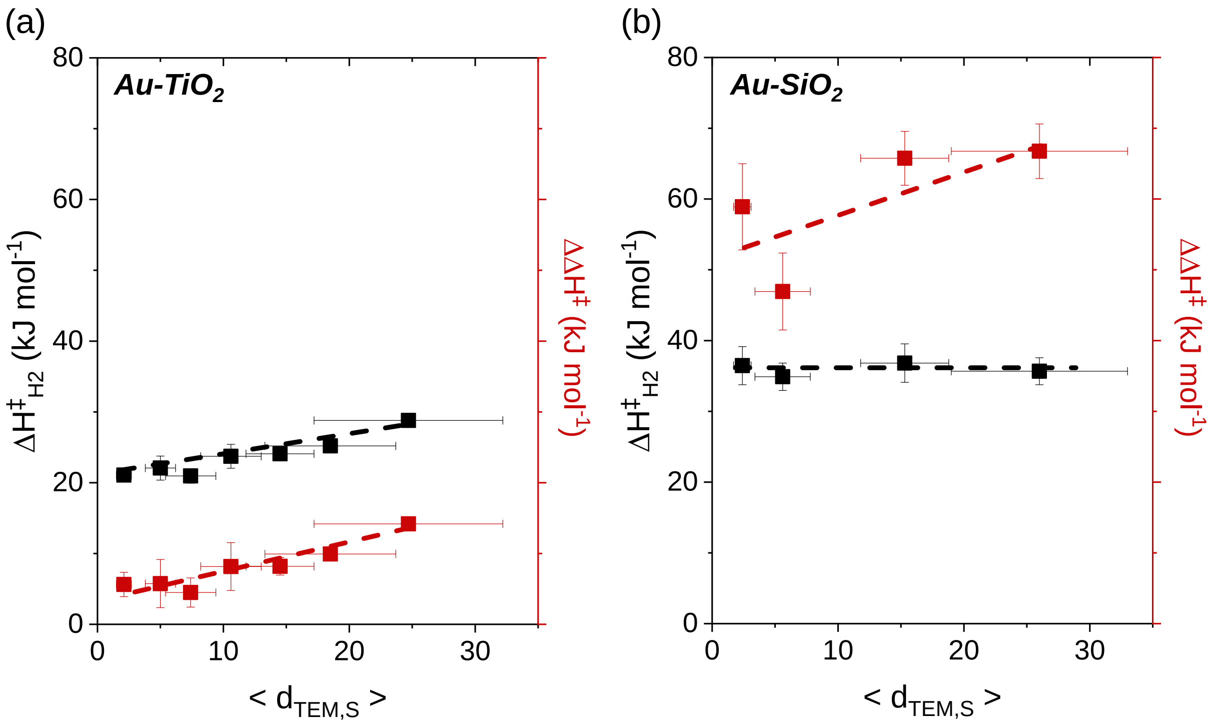

Figure S20. Apparent activation enthalpies of hydrogen consumption ($\Delta H^{\ddagger}_{H_2}$; ■) and differences in activation enthalpies of H_2O_2 and H_2O formation ($\Delta\Delta H^{\ddagger}$; ■) on (a) Au-$TiO_2$ and (b) Au-SiO_2 materials (200 kPa H_2, 60 kPa O_2, 278-308 K). Figures S18 and S19 show corresponding steady-state rate measurements as a function of temperature. Dashed lines intended to guide the eyes.

Figure S20 shows that the apparent barriers of H_2 consumption and the difference in barriers of H_2O and H_2O_2 formation increase with the size of Au nanoparticles on both Au-TiO_2 and Au-SiO_2, which are fitted from the data shown in Figures S18 and S19. Such observations suggest that a greater fraction of sites present greater barriers of activating H–H and O–O bonds as Au nanoparticles increase in size on Au-TiO_2.

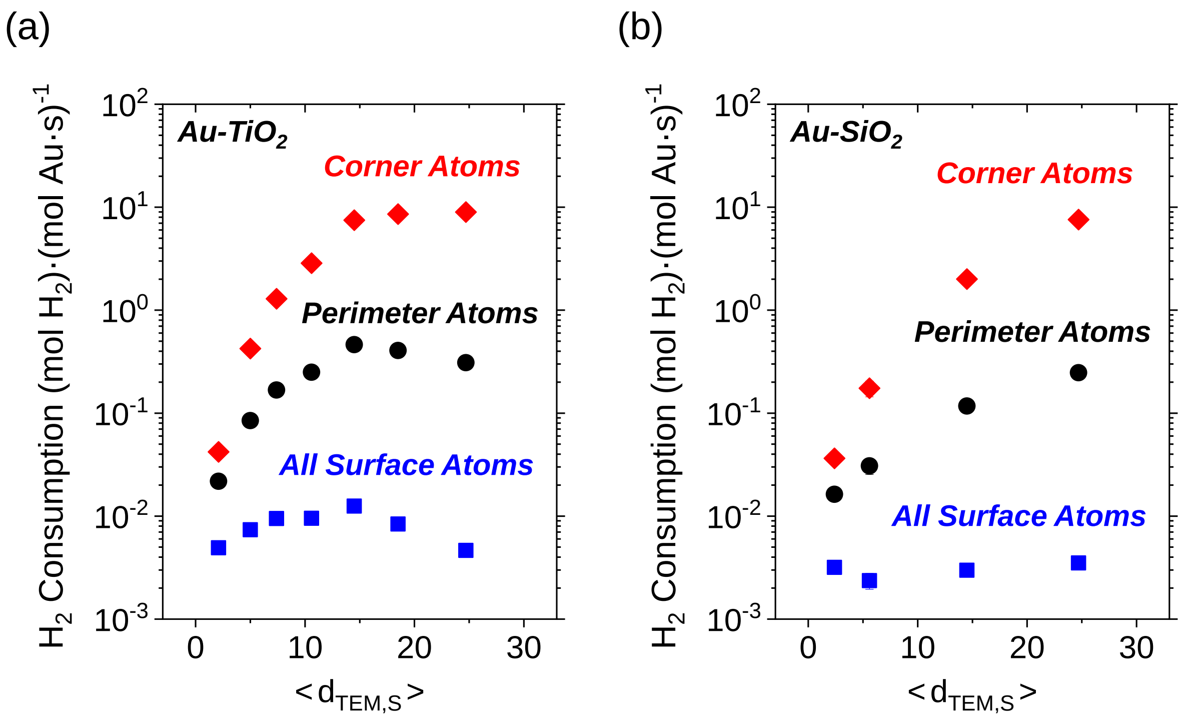

Figure S21. Steady-state rates of H_2 consumption as a function of the surface-area normalized Au nanoparticle diameter on (a) Au-TiO_2 and (b) Au-SiO_2 materials (200 kPa H_2, 60 kPa O_2, 278 K). Rates are normalized by the total number of surface atoms (■), perimeter atoms (●), and corner atoms (◆), estimated using correlations reported in equations S5.11-13.[31, 32] Note that this figure is identically reported as Figure 10 in the main text.

Figure S21 shows the rates of H_2 consumption as a function of the size of Au nanoparticles, normalized by the fraction of total surface atoms, perimeter atoms, and corner atoms using correlations for supported Au nanoparticles. These correlations were empirically reported by Ribeiro[31] and equivalently derived by Hereveld and Heretog,[32] assuming a truncated cuboctahedra geometry for the nanoparticles, as shown by

$$\theta_t = 0.9 \cdot (< d_{TEM,S} >)^{-0.7} \tag{S5.11}$$

$$\theta_p = 0.46 \cdot (< d_{TEM,S} >)^{-1.8} \tag{S5.12}$$

$$\theta_c = 0.54 \cdot (< d_{TEM,S} >)^{-2.9} \tag{S5.13}$$

where θ_t is the total fraction of Au atoms that exist on surfaces of nanoparticles, θ_p represents the fraction of Au atoms that reside at the perimeters of nanoparticles in contact with the support (i.e., exposed at the interface with the support), and θ_c signifies the fraction of Au atoms located at the corners of nanoparticles that contact the support. All values are calculated from the average nanoparticle diameters ($< d_{TEM,S} >$).

These calculations consider multiple possible active sites. If perimeter or corner atoms are the primary active site for H_2 activation and consumption, then the intrinsic reactivity of these sites would need to increase by orders of magnitude to agree with experimental measurements (Figure S21), and these differences in rates would likely reflect decreasing apparent activation enthalpies. Figure S20, however, shows that values of $\Delta H^{\ddagger}_{H_2}$ either increase (Au-TiO_2) or remain constant (Au-SiO_2) with increasing mean diameters for Au nanoparticles. In contrast, rates normalized by the total number of Au atoms exposed at surfaces show a slight dependence upon Au nanoparticle size on Au-TiO_2 (spanning rates that differ by a factor of ~ 2) and remain nearly constant on Au-SiO_2. These near invariance of H_2 consumption rates normalized by the total number of Au atoms appears most consistent with the size dependence of $\Delta H^{\ddagger}_{H_2}$ values (Figure S20). Consequently, comparisons among normalized rates for H_2 consumption and $\Delta H^{\ddagger}_{H_2}$ values suggest that all exposed Au atoms contribute to kinetically relevant processes for H_2 activation and consumption. Thus, multiple types of sites (i.e., interfacial, corner, and highly-coordinated sites) may contribute to catalysis on Au-TiO_2.

Overall, the findings reported in Sections 3.1 and 3.4 show the complex site motifs at play during the activation of small molecules. Specifically, H_2 and O_2 may bind directly to Au nanoparticles or to interfacial Au atoms at the perimeter of Au nanoparticles and the support. Sites at this metal-support interface strongly affect the barrier of dissociating O–O bonds and seem to act as the primary site of H_2O formation. However, the remaining surface sites on the Au nanoparticles may bind dioxygen much more weakly and act as the primary sites of forming H_2O_2. Still, these sites only seem to form appreciable H_2O_2 when liquid water molecules solvate them. By comparison, H_2 seems to activate on all surface Au atoms with similar barriers; however, the presence of OH groups appears crucial to enabling high rates of dissociating H–H bonds. Here, nucleophilic or Bronsted base functions seem to influence catalysis far from the metal-support interface. Thus, the solvation of these catalytic sites by liquid H_2O may extend the range of catalysis beyond just the perimeter of Au nanoparticles.

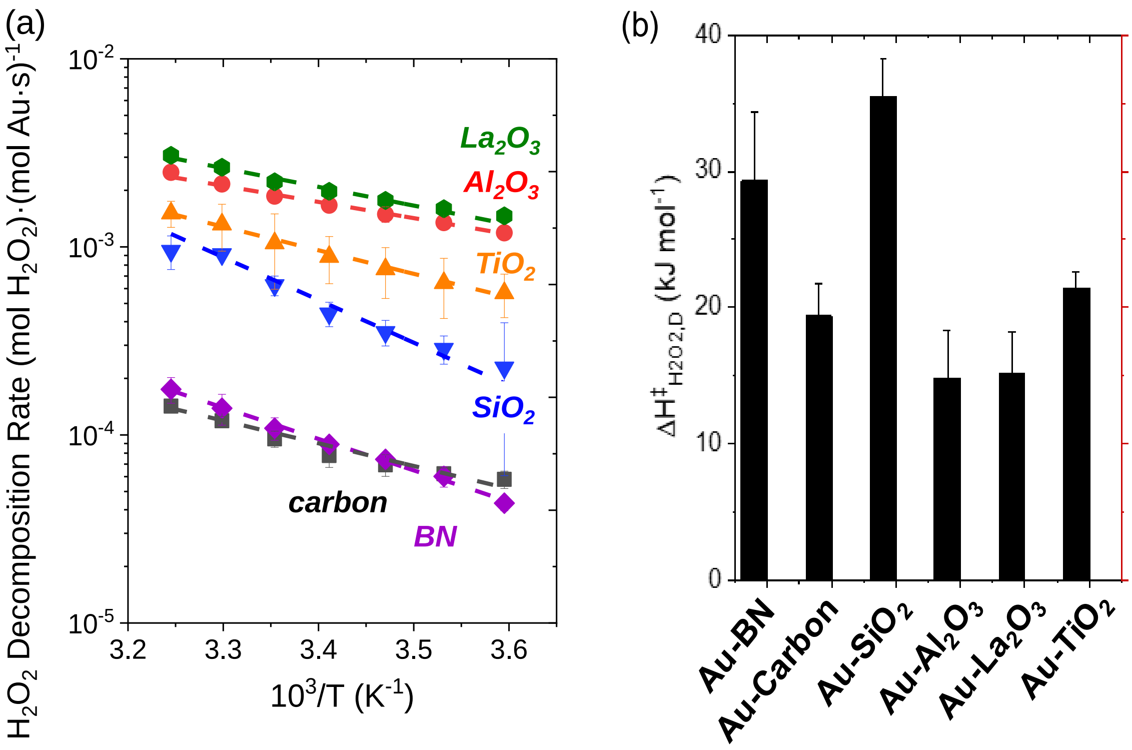

Figure S22. Steady-state rates of (a) H_2O_2 decomposition as a function of inverse temperature (278-308 K), and (b) calculated activation enthalpies ($\Delta H^{\ddagger}_{H_2O_2,D}$) on 2-3 nm Au nanoparticles supported on BN (♦), Carbon (■), SiO_2 (▼), Al_2O_3 (●), La_2O_3 (⬢), and TiO_2 (▲) at standard conditions (200 kPa H_2, 0.2 mM H_2O_2). The dashed lines represent fits to equation S5.1 and give apparent activation enthalpies.

Figure S22a shows temperature dependence profiles of secondary H_2O_2 decomposition over Au nanoparticles supported on BN, Carbon, SiO_2, Al_2O_3, La_2O_3, and TiO_2 (200 kPa H_2, 0.2 M H_2O_2, 278-308 K). Fits of these profiles using the Eyring equation (equation S5.1) yields values for the activation enthalpies of H_2O_2 dissociation ($\Delta H^{\ddagger}_{H_2O_2,D}$, *vide supra*), which correlate with the values of $\Delta\Delta H^{\ddagger}$ shown in Figures S23 and 10 on the same materials. Again, the data suggest that more refractory Au-support interfaces (e.g., Au-SiO_2) weakly activate O–O bonds compared to Au-nanoparticles bound to more reducible supports (e.g., Au-La_2O_3). Similarly, the higher barriers of O–O dissociation data agree with the higher H_2O_2 selectivity on refractory materials, as shown in Figure 3. Further interpretation of these data is reported in Section 3.4 and Figure 10.

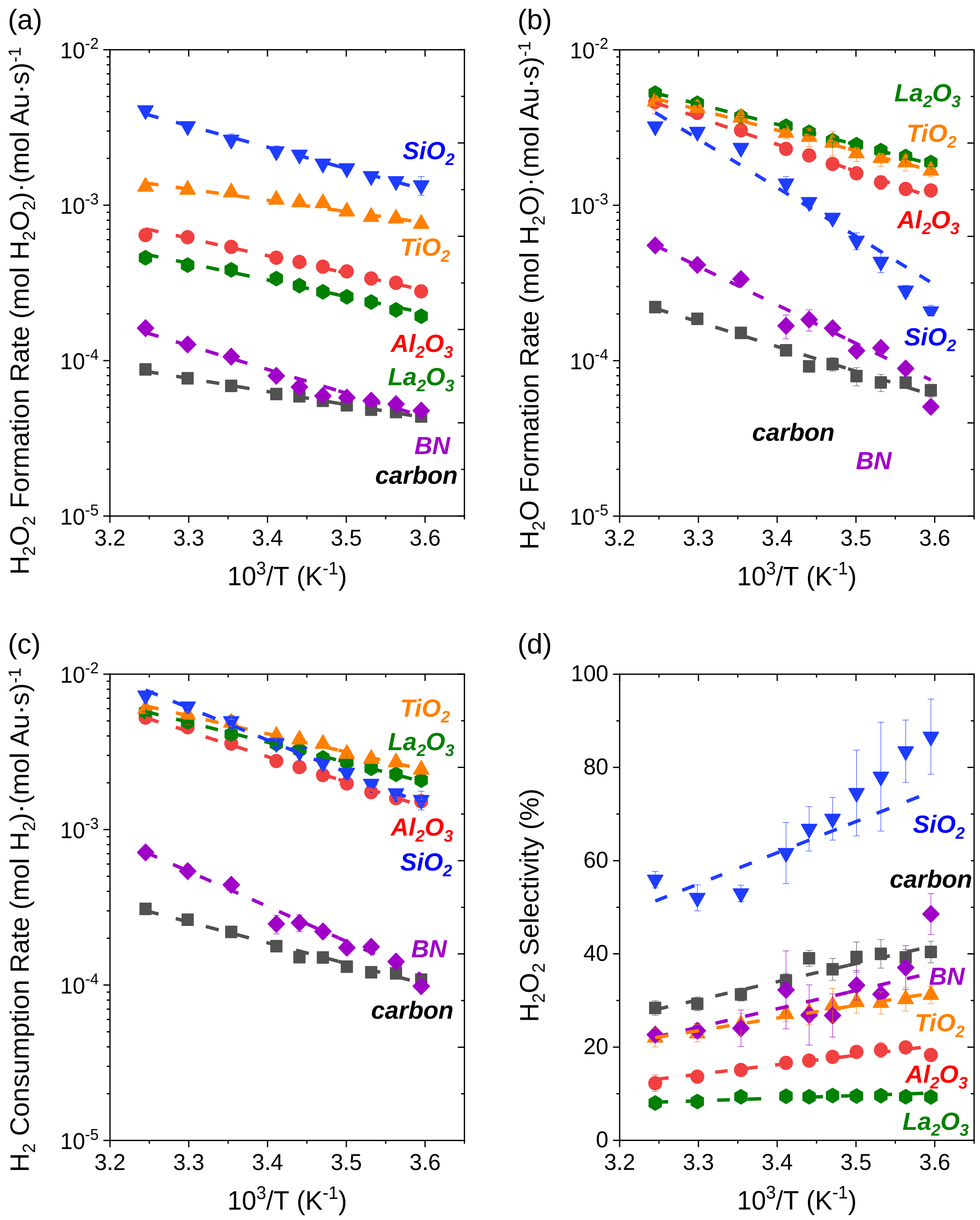

Figure S23. Steady-state rates of (a) H_2O_2 formation, (b) H_2O formation, (c) H_2 consumption, and (d) H_2O_2 selectivity as a function of inverse temperature (278-308 K) on 2-3 nm Au nanoparticles supported on BN (♦), Carbon (■), SiO_2 (▼), Al_2O_3 (●), La_2O_3 (⬢), and TiO_2 (▲) at standard conditions (200 kPa H_2, 60 kPa O_2). The dashed lines represent fits to equation S5.1 and give apparent activation enthalpies shown in Figure 10.

Figure S23 shows the temperature dependence of H_2 consumption, H_2O_2 formation, H_2O formation, and H_2O_2 selectivity over Au nanoparticles supported on BN, Carbon, SiO_2, Al_2O_3, La_2O_3, and TiO_2 (278-308 K). Fits of these profiles using the Eyring equation (equation S5.1) yield values for the apparent activation enthalpies of H_2 consumption, H_2O_2 formation, and H_2O formation (*vide supra*). Note that the fits for Au-SiO_2 were evaluated in the linear region of this Arrhenius plot and were evaluated between 278-298K for this data set, equivalently shown in Figure S19. Further analysis of the difference in apparent activation barriers of H_2 consumption and H_2O_2 and H_2O formation lead to the definitions of $\Delta H^{\ddagger}_{H_2}$ and $\Delta\Delta H^{\ddagger}$shown by equations S5.6 and S5.9, respectively. These data agree with trends in H_2 consumption and H_2O_2 selectivity shown in Figure 3 and the apparent barriers of H_2O_2 decomposition shown in Figure S22, which show that more basic and reducible supports enable high rates of H_2 activation and favor H_2O formation. In contrast, more refractory supports like SiO_2 favor H_2O_2 formation.

Section S6: Supplemental Analysis of the Kinetic Consequences of Alloying Au with Pd

Elucidating the role of Au-support interfaces on the activation of H–H and O–O bonds provide useful insights for intentionally catalyzing the formation of H_2O_2 from H_2 and O_2. Such systems typically involve alloys of noble metal nanoparticles (e.g., $PdAu_x$ and $PtAu_x$) in protic solvents,[2, 5, 33-37, 24, 38, 39] which favor H_2O_2 formation compared to monometallic Pd or Pt. [5, 18, 20, 34, 36, 40-45] However, these studies typically do not disentangle the effects of alloy composition from that of the support identity.[42, 46] For example, recent work from our group suggests SiO_2-supported $PdAu_x$ nanoparticles achieve selectivities that can exceed 90% with increasing dilution of Pd on Au nanoparticles.[5] In contrast, prior investigations of TiO_2-supported $PdAu_x$ alloys show non-monotonic increases in H_2O_2 selectivities as the ratio of Au to Pd increases,[40-42] which were interpreted as evidence that sites at Au-TiO_2 interfaces favor the dissociation of O–O bonds.[42, 47-50] Thus, the Au materials of this study were alloyed with Pd to understand the kinetic consequences of alloying supported Au nanoparticles.

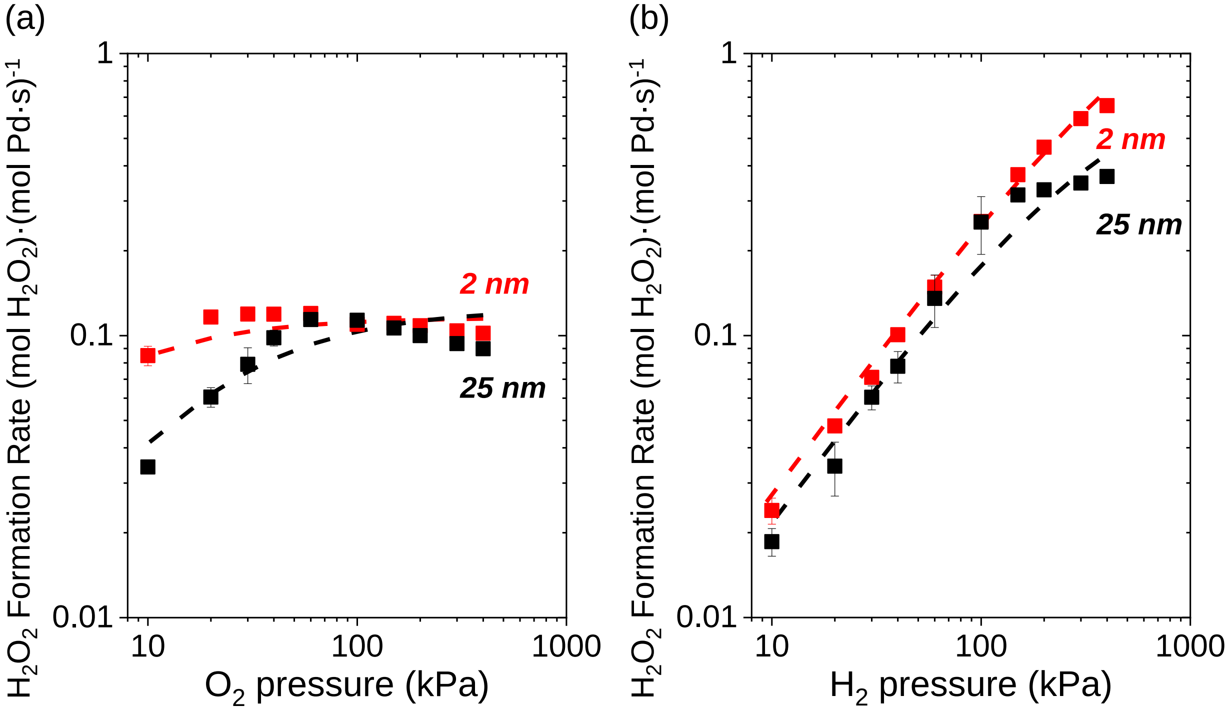

Figure S24. Steady-state rates of H_2O_2 formation on 2 nm (red) and 25 nm (black) Pd_1Au_{100} nanoparticles supported on TiO_2 as a function of the pressure of (a) O_2 (10-400 kPa H_2, 60 kPa O_2) and (b) H_2 (10-400 kPa O_2, 60 kPa H_2) at 278 K. Dashed lines fitted to equation 7 for H_2 dependence or to guide the eyes for O_2 dependence.

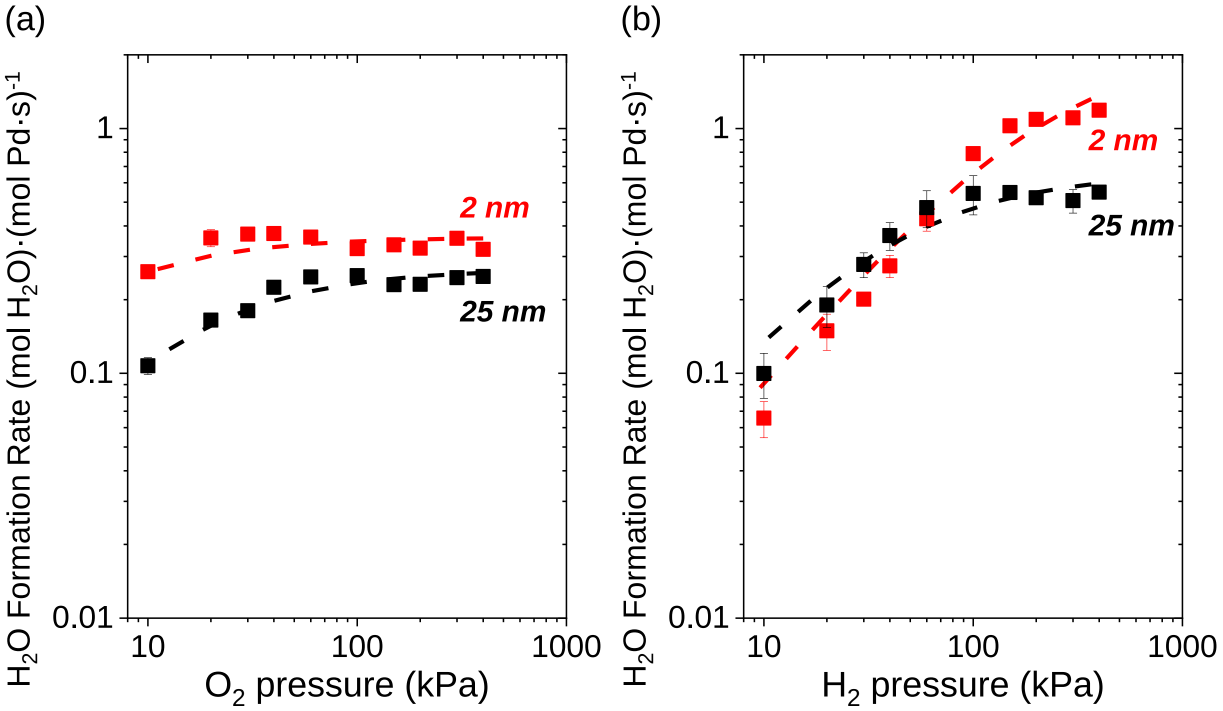

Figure S25. Steady-state rates of H_2O formation on (a) 2 nm (■) and (b) 25 nm (■) $PdAu_{100}$ nanoparticles supported on TiO_2 as a function of the pressure of H_2 (10-400 kPa H_2, 60 kPa O_2) and O_2 (10-400 kPa O_2, 60 kPa H_2) at 278 K. Dashed lines fitted to equation 7 for H_2 dependence or to guide the eyes for O_2 dependence.

Figures S24 and S25 show the steady-state rates of H_2O_2 and H_2O formation (normalized by moles of alloyed Pd) as functions of H_2 (10-400 kPa) and O_2 (10-400 kPa) pressures on TiO_2-supported Pd_1Au_{100} nanoparticles (2-25 nm) at 278 K. Here, rates increase sublinearly (10-60 kPa O_2) before approaching a constant value (60-400 kPa O_2) on $PdAu_{100}$ alloys (Figures S24a and S25a). In contrast, monometallic Au materials show little dependence on the pressure of O_2 (Figures 4a and 5a). These differences suggest oxygen-derived intermediates saturate the active sites of Au nanoparticles; however, Pd moieties require a greater chemical potential of O_2 to achieve full coverage. By comparison, rates increase in proportion to the pressure of H_2 on both the $PdAu_{100}$ (Figures S24b and S25b) and Au catalysts (Figures 4b and 5b) but approach a constant value on the $PdAu_{100}$ at the greatest pressures (>150 kPa H_2). These observations suggest hydrogen adatoms saturate the surface of $PdAu_{100}$ as pressures of H_2 increase and imply H-atoms bind more strongly to $PdAu_{100}$ than monometallic Au. Moreover, this saturation behavior becomes more pronounced on the larger-sized particles of $PdAu_{100}$, suggesting that a greater fraction of surface Pd on such materials may lead to stronger binding of H_2-derived species on these catalysts. These findings agree with mechanisms involving the simultaneous activation of $H^{\#}$ and O_2^* on analogous $PdAu_x$ catalysts.[5] The $PdAu_{100}$ materials show H_2O_2 selectivities less than or equal to those for monometallic Au catalysts (~25-55%) at identical conditions (Figure S26), which indicates alloyed Pd atoms may destabilize O–O bonds.

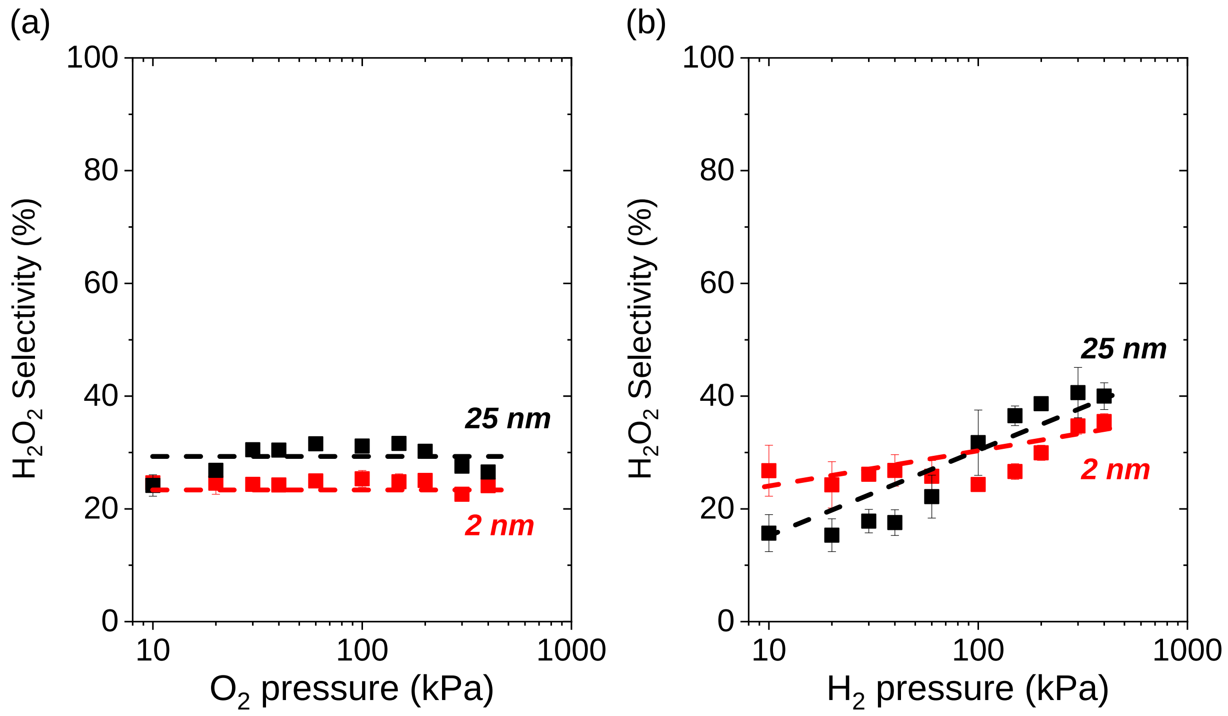

Figure. S26. Steady-state H_2O_2 selectivities on $PdAu_{100}$ nanoparticles with mean diameters of (a) 2 nm (■) and (b) 25 nm (■) supported on TiO_2 as a function of the pressure of H_2 (10-400 kPa H_2, 60 kPa O_2) and O_2 (10-400 kPa O_2, 60 kPa H_2) at 278 K. Dashed lines intended to guide the eyes.

Figure S26 shows selectivities of H_2O_2 formation as a function of the pressure of H_2 and O_2 on $PdAu_{100}$ nanoparticles of varying sizes (2 or 25 nm) supported on TiO_2. Selectivities are mostly independent of the pressure of O_2 but increase with the pressure of H_2, resembling the trends observed on monometallic Au nanoparticles. Such findings suggest that high coverages of H_2-derived species increase the stability of O–O bonds, as discussed in Section 3.2.

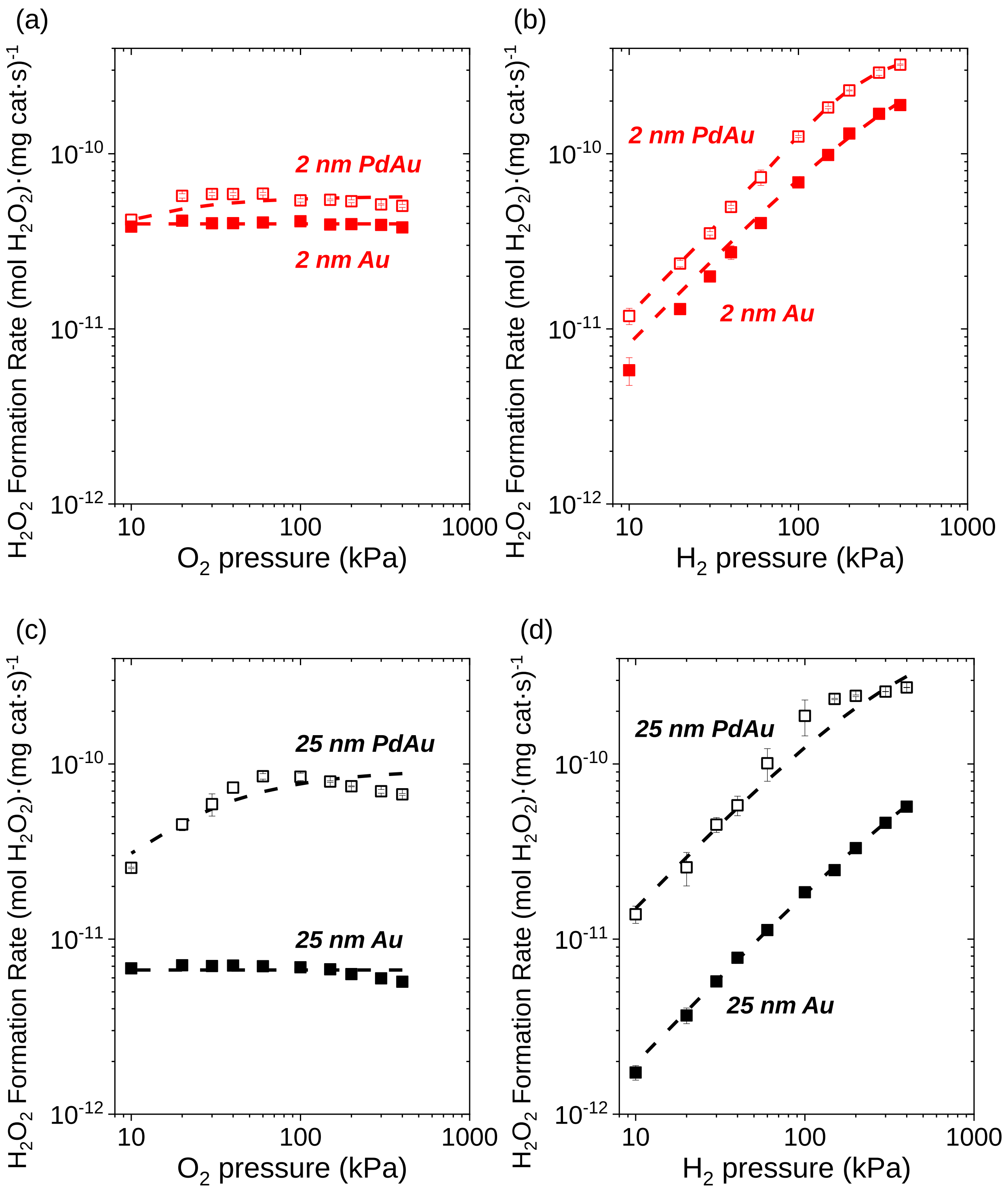

Figure S27. Steady-state H_2O_2 formation rates as functions of the pressure of (a,c) O_2 (10-400 kPa O_2, 60 kPa H_2), and (b,d) H_2 (10-400 kPa H_2, 60 kPa O_2) at 278 K. Rates were obtained from 2 nm Au (■), 2 nm $PdAu_{100}$ (□), 25 nm Au (■), and 25 nm $PdAu_{100}$ (□) nanoparticles supported on TiO_2. Dashed lines fitted to equation 7 for H_2 dependence or to guide the eyes for O_2 dependence.

Figure S27 shows rates of H_2O_2 formation as a function of the pressure of H_2 and O_2 on Au and $PdAu_{100}$ nanoparticles of varying sizes (2 or 25 nm) supported on TiO_2. Notably, the addition of Pd leads to a 3-10 fold increase in reactivity relative to the mother materials, consistent with a significant increase in the reactivity of H_2 on the alloyed materials. For comparison, rates for $PdAu_{100}$ catalysts are 100 to 1000 times greater than for monometallic Au when normalized by moles of Pd (Figure 12) versus moles of Au (Figures 4 and 10). These observations agree with past work showing $PdAu_x$ nanoparticles present much greater rates of H_2O_2 formation than monometallic Au catalysts.[5, 34, 36, 40-42, 51-53] Moreover, the presence of Pd leads to a change in the functional dependence on H_2 and O_2, indicating that the surface Pd atom changes the free energy of binding these reactants, as discussed in Section 3.5.

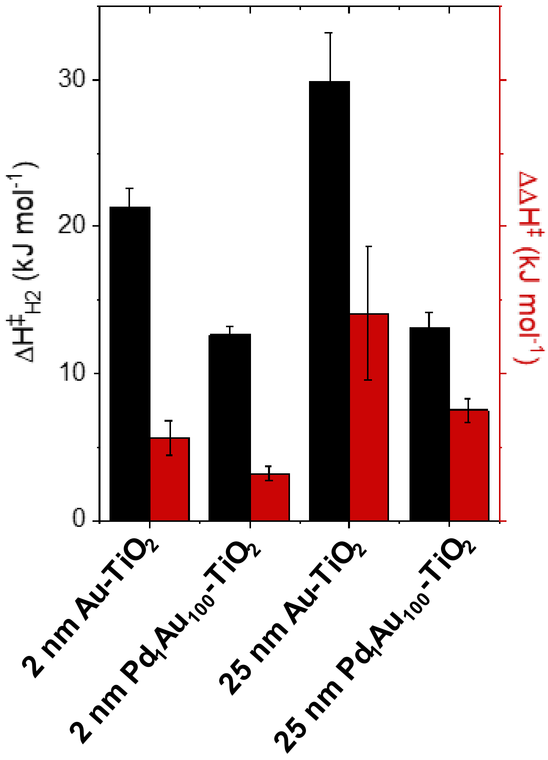

Figure S28. Apparent activation enthalpies of hydrogen consumption ($\Delta H^{\ddagger}_{H_2}$; black, ■) and differences in activation enthalpies of H_2O_2 and H_2O formation ($\Delta\Delta H^{\ddagger}$; red) on Au and Pd_1Au_{100} nanoparticles (2 nm and 25 nm) supported on TiO_2 (200 kPa H_2, 60 kPa O_2, 278-308 K).

The identity of the support significantly impacts rates and selectivities for reactions among H_2 and O_2 upon dilute $PdAu_x$ alloy nanoparticles. For example, $PdAu_x$ nanoparticles show much greater selectivities for H_2O_2 formation when supported on SiO_2 (25-95%)[5] compared to those of similar size and composition on TiO_2 (~25-55%; Figure S26). These differences stem from the influence of both Pd addition and support identity on values for $\Delta H^{\ddagger}_{H_2}$ and $\Delta\Delta H^{\ddagger}$ (Figure S28). Here, the addition of Pd atoms to Au-TiO_2 decreases values of $\Delta H^{\ddagger}_{H_2}$ by 10-17 kJ mol^{-1} while only slightly reducing $\Delta\Delta H^{\ddagger}$ values (by 2-7 kJ mol^{-1}). In comparison, SiO_2-supported catalysts achieve much greater values of $\Delta\Delta H^{\ddagger}$ (17-55 kJ mol^{-1})[5] than TiO_2-supported materials (3-15 kJ mol^{-1}). However, this strategy is most effective when isolating Pd atoms on the surface of Au nanoparticles since

H_2O_2 selectivities and values of $\Delta\Delta H^{\ddagger}$ decrease significantly as the coverage of Pd increases.[5] This understanding presents opportunities to design even more selective and reactive catalysts by exploiting the high reactivity of Pd towards H_2 activation and the high stability of O–O bonds on mostly inert metal-support interfaces (e.g., Au-SiO_2).

References

1. Patterson, A. L., The Scherrer Formula for X-Ray Particle Size Determination. *Physical Review* **1939,** *56* (10), 978.
2. Adams, J. S.; Chemburkar, A.; Priyadarshini, P.; Ricciardulli, T.; Lu, Y.; Maliekkal, V.; Sampath, A.; Winikoff, S.; Karim, A. M.; Neurock, M.; Flaherty, D. W., Solvent molecules form surface redox mediators in situ and cocatalyze O2 reduction on Pd. *Science* **2021,** *371*, 626-632.
3. Stiehl, J. D.; Kim, T. S.; McClure, S. M.; Mullins, C. B., Evidence for Molecularly Chemisorbed Oxygen on TiO2 Supported Gold Nanoclusters and Au(111). *Journal of the American Chemical Society* **2004,** *126* (6), 1606-1607.
4. Sanchez-Sanchez, C. M.; Bard, A. J., Hydrogen peroxide production in the oxygen reduction reaction at different electrocatalysts as quantified by scanning electrochemical microscopy. *Analytical Chemistry* **2009,** *81* (19), 8094-8100.
5. Ricciardulli, T.; Gorthy, S.; Adams, J. S.; Thompson, C.; Karim, A. M.; Neurock, M.; Flaherty, D. W., Effect of Pd Coordination and Isolation on the Catalytic Reduction of O_2 to H_2O_2 over PdAu Bimetallic Nanoparticles. *Journal of the American Chemical Society* **2021,** *143*, 5445-5464.
6. Podlibner, B. A.; Nekrasov, L. N., *Electrokhimiya* **1960,** *5*, 340.
7. Zurilla, R. W.; Sen, R. K.; Yeager, E., The Kinetics of the Oxygen Reduction Reaction on Gold in Alkaline Solution. *Journal of the Electrochemical Society* **1978,** *125*, 1103– 1109.
8. Strobl, J. R.; Scherson, D., On the Mechanism of the Oxygen Reduction Reaction on Au(poly) in Aqueous Alkaline Electrolytes: A Critical Reassessment. *Journal of Physical Chemistry C* **2021** *125* (25), 13862-13870.
9. Mei, D.; He, Z. D.; Zheng, Y. L.; Jiang, D. C.; Chen, Y., Mechanistic and kinetic implications on the ORR on a Au(100) electrode: pH, temperature and H–D kinetic isotope effects. *Physical Chemistry Chemical Physics* **2014,** *16* (27), 13762-13772.
10. Whittaker, T.; Kumar, K. B. S.; Peterson, C.; Pollock, M. N.; Grabow, L. C.; Chandler, B. D., H_2 Oxidation over Supported Au Nanoparticle Catalysts: Evidence for Heterolytic H2 Activation at the Metal–Support Interface. *Journal of the American Chemical Society* **2018,** *140* (48), 16469-16487.
11. Kumar, K. B. S.; Whittaker, T.; Peterson, C.; Grabow, L. C.; Chandler, B. D., Water Poisons H_2 Activation at the Au–TiO_2 Interface by Slowing Proton and Electron Transfer between Au and Titania. *Journal of the American Chemical Society* **2020,** *142* (12), 5760-5772.
12. Mahdavi-Shakib, A.; Kumar, K. B. S.; Whittaker, T.; Xie, T.; Grabow, L. C.; Rioux, M. R.; Chandler, B. D., Kinetics of H_2 Adsorption at the Metal–Support Interface of Au/TiO_2 Catalysts Probed by Broad Background IR Absorbance. *Angewandte Chemie-International Edition* **2021,** *60* (14), 7735-7743.
13. Wan, W.; Nie, X.; Janik, M. J.; Song, C.; Guo, X., Adsorption, Dissociation, and Spillover of Hydrogen over Au/TiO2 Catalysts: The Effects of Cluster Size and Metal–Support Interaction from DFT. **2018,** *122* (31), 17895-17916.
14. Panayotov, D. A.; Burrows, S. P.; Yates, J. T.; Morris, J. R., Mechanistic Studies of Hydrogen Dissociation and Spillover on Au/TiO_2: IR Spectroscopy of Coadsorbed CO and H-Donated Electrons. *Journal of Physical Chemistry C* **2011,** *115* (45), 22400-22408.
15. Panayotov, D. A.; Yates, J. T., n-Type doping of TiO2 with atomic hydrogen-observation of the production of conduction band electrons by infrared spectroscopy. *Chemical Physics Letters* **2007,** *436* (1-3), 204-208.

16. Panayotov, D. A.; Yates, J. T., Spectroscopic Detection of Hydrogen Atom Spillover from Au Nanoparticles Supported on TiO2: Use of Conduction Band Electrons. *Journal of Physical Chemistry C* **2007,** *111* (7), 2959-2964.
17. Davydova, E. S.; Mukerjee, S.; Jaouen, F.; Dekel, D. R., Electrocatalysts for hydrogen oxidation reaction in alkaline electrolytes. *Acs Catalysis* **2018,** *8* (7), 6665-6690.
18. Bond, G. C.; Thompson, D. T., Catalysis by Gold. *Catalysis Reviews* **1999,** *41* (3-4), 319-388.
19. Zhao, J.; Ge, L.; Yuan, H.; Liu, Y.; Gui, Y.; Zhang, B.; Zhou, L.; Fang, S., Heterogeneous gold catalysts for selective hydrogenation: from nanoparticles to atomically precise nanoclusters. *Nanoscale* **2019,** *11* (24), 11429-11436.
20. Hammer, B.; Norskov, J. K., Why gold is the noblest of all the metals *Nature* **1995,** *376*, 238-240.
21. Parks, G. A., The isoelectric points of solid oxides, solid hydroxides, and aqueous hydroxo complex systems. *Chemical Reviews* **1964,** *65* (2), 177-195.
22. Tse, E. C. M.; Varnell, J. A.; Hoang, T. T. H.; Gewirth, A. A., Elucidating proton involvement in the rate-determining step for Pt/Pd-based and non-precious-metal oxygen reduction reaction catalysts using the kinetic isotope effect. *Journal of Physical Chemistry Letters* **2016,** *7* (18), 3542-3547.
23. Sivadinarayana, C.; Choudhary, T. V.; Daemen, L. L.; Eckert, J.; Goodman, D. W., The Nature of the Surface Species Formed on Au/TiO2 during the Reaction of H2 and O2: An Inelastic Neutron Scattering Study. *Journal of the American Chemical Society* **2004,** *126* (1), 38-39.
24. Dissanayake, D.; Lunsford, J. H., The direct formation of H_2O_2 from H_2 and O_2 over colloidal palladium. *Journal of Catalysis* **2003,** *214*, 113-120.
25. Powell, C. D.; Daigh, A. W.; Pollock, M. N.; Chandler, B. D.; Pursell, C. J., CO Adsorption on Au/TiO2 Catalysts: Obervations, Quantification, and Explanation of Broad-band Infrared Signal. *Journal of physical Chemistry C* **2017,** *121* (44), 24541-24547.
26. Clack, J. C.; Dai, S.; Overbury, S. H., Operando studies of desorption, reaction and carbonate formation during CO oxidation by Au/TiO2 catalysts. *Catalysis Today* **2007,** *126* (1-2), 135-142.
27. Chang, B.; Jang, B. W.; Dai, S.; Overbury, S. H., Transient studies of the mechanisms of CO oxidation over Au/TiO2 using time-resolved FTIR spectroscopy and product analysis. *Journal of Catalysis* **2005,** *236*, 392-400.
28. Litke, A.; Su, Y.; Tranca, I.; Weber, T.; Hensen, E. J. M.; Hofmann, J. P., Role of Adsorbed Water on Charge Carrier Dynamics in Photoexcited TiO2. *Journal of physical Chemistry C* **2017,** *121* (13), 7514-7524.
29. Schumacher, B.; Plzak, V.; Kinne, M.; Behm, R. J., Highly active Au/TiO2 catalyst for low-temperature CO oxidation: Preparation, conditioning and stability. *Catalysis Letters* **2003,** *89* (1-2), 109-114.
30. Denkwitz, Y.; Zhao, Z.; Hormann, U.; Kaiser, U.; Plzak, V.; Behm, R. J., Stability and deactivation of unconditioned Au/TiO2 catalysts during CO oxidation in a near-stoichiometric and O2-rich reaction atmosphere. *Journal of Catalysis* **2007,** *251*, 363-373.
31. Shekhar, M.; Wang, J.; Lee, W.; Williams, W. D.; Kim, S. M.; Stach, E. A.; Miller, J. T.; Delgass, W. N.; Ribeiro, F. H., Size and Support Effects for the Water–Gas Shift Catalysis over Gold Nanoparticles Supported on Model Al2O3 and TiO2. *Journal of the American Chemical Society* **2012,** *134* (10), 4700-4708.

32. Van Hardeveld, R.; Hartog, F., The Statistics of Surface Atoms and Surface Sites on Metal Crystals. *Surface Science* **1969,** *15* (2), 189-230.
33. Wilson, N. M.; Flaherty, D. W., Mechanism for the Direct Synthesis of H2O2 on Pd Clusters: Heterolytic Reaction Pathways at the Liquid-Solid Interface. *Journal of the American Chemical Society* **2016,** *138* (2), 574-586.
34. Wilson, N. M.; Priyadarshini, P.; Kunz, S.; Flaherty, D. W., Direct synthesis of H_2O_2 on Pd and Au_xPd_1 clusters: Understanding the effects of alloying Pd with Au. *Journal of Catalysis* **2018,** *357*, 163-175.
35. Wilson, N. M.; Schroder, J.; Priyadarshini, P.; Bregante, D. T.; Kunz, S.; Flaherty, D. W., Direct synthesis of H_2O_2 on PdZn nanoparticles: The impact of electronic modifications and heterogeneity of active sites. *Journal of Catalysis* **2018,** *368*, 261-274.
36. Agarwal, N.; Thomas, L.; Nasrallah, A.; Sainna, M. A.; Freakley, S. J.; Edwards, J. K.; Catlow, R. A.; Hutchings, G. J.; Taylor, S. H.; Willock, D., The direct synthesis of hydrogen peroxide over Au and Pd nanoparticles: A DFT study. *Catalysis Today* **2020,** *381*, 76-85.
37. Kim, M.; Han, S. S., Electrochemically Modeling a Nonelectrochemical System: Hydrogen Peroxide Direct Synthesis on Palladium Catalysts. *Journal of Physical Chemistry Letters* **2021,** *12* (19), 4490-4495.
38. Ford, D. C.; Nilekar, A. U.; Xu, Y.; Mavrikakis, M., Partial and complete reduction of O2 by hydrogen on transition metal surfaces. *Surface Science* **2010,** *604* ((19-20)), 1565-1575.
39. Potts, D. S.; Bregante, D. T.; Adams, J. S.; Torres, C.; Flaherty, D. W., Influence of Solvent Structure and Hydrogen Bonding on Catalysis at Solid-Liquid Interfaces. *Chemical Society Reviews* **2021,** *50*, 12308-12337.
40. Edwards, J. K.; Solsona, B. E.; Landon, P.; Carley, A. F.; Herzing, A. A.; Kiely, C. J.; Hutchings, G. J., Direct synthesis of hydrogen peroxide from H2 and O2 using TiO2-supported Au–Pd catalysts. *Journal of Catalysis* **2005,** *236* (1), 69-79.
41. Edwards, J. K.; Ntainjua, E.; Carley, A. F.; Herzing, A. A.; Kiely, C. J.; Hutchings, G. J., Direct Synthesis of H_2O_2 from H_2 and O_2 over Gold, Palladium, and Gold–Palladium Catalysts Supported on Acid-Pretreated TiO_2. *Angewandte Chemie-International Edition* **2009,** *48* (45), 8512-8515.
42. Ouyang, L.; Da, G.; Tian, P.; Chen, T.; Liang, G.; Xu, J.; Han, Y. F., Insight into active sites of Pd–Au/TiO_2 catalysts in hydrogen peroxide synthesis directly from H_2 and O_2. *Journal of Catalysis* **2014,** *311*, 129-136.
43. Forde, M. M.; Armstrong, R. D.; Hammond, C.; He, Q.; Jenkins, R. L.; Kondrat, S. A.; Dimitratos, N.; Lopez-Sanchez, J. A.; Taylor, S. H.; Willock, D.; Kiely, C. J.; Hutchings, G. J., Partial oxidation of ethane to oxygenates using Fe- and Cu-Containing ZSM-5. *Journal of the American Chemical Society* **2013,** *135* (30), 11087-11099.
44. Zhao, Y.; Adams, J. S.; Baby, A.; Kromer, M.; Flaherty, D. W.; Rodriguez, J., Electrochemical Screening of Au/Pt Catalysts for the Thermocatalytic Synthesis of Hydrogen Peroxide Based on Their Oxygen Reduction and Hydrogen Oxidation Activity Probed via Voltammetric Scanning Electrochemical Microscopy. *ACS Sustainable Chemical Engineering* **2022,** *10* (51), 17207-17220.
45. Priyadarshini, P.; Ricciardulli, T.; Adams, J. S.; Yun, Y. S.; Flaherty, D. W., Effects of Bromide Adsorption on the Direct Synthesis of H2O2 on Pd Nanoparticles: Formation Rates, Selectivities, and Apparent Barriers at Steady-State. *Journal of Catalysis* **2021,** *399*, 24-40.

46. Edwin, N. N.; Edwards, J. K.; Carley, A. F.; Lopez-Sanchez, J. A.; Moulijn, J. A.; Herzing, A. A.; Kiely, C. J.; Hutchings, G. J., The role of the support in achieving high selectivity in the direct formation of hydrogen peroxide. *Green Chemistry* **2008,** *10* (11), 1162-1169.
47. Green, I. X.; Tang, W.; Neurock, M.; Yates, J. T., Insights into Catalytic Oxidation at the Au/TiO2 Dual Perimeter Sites. *Accounts of Chemical Research* **2014,** *47* (3), 805-815.
48. Green, I. X.; Tang, W.; Neurock, M.; Yates, J. T., Spectroscopic Observation of Dual Catalytic Sites During Oxidation of CO on a Au/TiO2 Catalyst. *Science* **2011,** *333* (6043), 736-739.
49. Green, I. X.; Tang, W.; McEntee, M.; Neurock, M.; Yates, J. T., Inhibition at Perimeter Sites of Au/TiO_2 Oxidation Catalyst by Reactant Oxygen. *Journal of the American Chemical Society* **2012,** *134* (30), 12717-12723.
50. Green, I. X.; Tang, w.; Neurock, M.; Yates, J. T., Low-Temperature Catalytic H_2 Oxidation over Au Nanoparticle/TiO_2 Dual Perimeter Sites. *Angewandte Chemie-International Edition* **2011,** *50* (43), 10186-10189.
51. Piccinini, M.; Ntainjua, E.; Edwards, J. K.; Carley, A. F.; Moulijn, J. A.; Hutchings, G. J., Effect of the reaction conditions on the performance of Au-Pd/TiO2 catalyst for the direct synthesis of hydrogen peroxide. *Physical Chemistry Chemical Physics* **2010,** *12* (10), 2488-2492.
52. Ham, H. C.; Stephens, J. A.; Hwang, G. S.; Han, J.; Nam, S. W.; Lim, T. H., Pd ensemble effects on oxygen hydrogenation in AuPd alloys: A combined density functional theory and Monte Carlo study. *Catalysis Today* **2011,** *165*, 138-144.
53. Kulkarni, A.; Siahrostami, S.; Patel, A.; Norskov, J. K., Understanding Catalytic Activity Trends in the Oxygen Reduction Reaction. *Chemical Reviews* **2018,** *118* (5), 2302-2312.
